# Internal transcribed spacer 2 (ITS2) molecular morphometric analysis based species delimitation of foliar endophytic fungi from *Aglaia elaeagnoidea*, *Flacourtia inermis* and *Premna serratifolia*

**DOI:** 10.1371/journal.pone.0215024

**Published:** 2019-04-09

**Authors:** Natesan Sundaresan, Enthai Ganeshan Jagan, GokulRaj Kathamuthu, Mohan Pandi

**Affiliations:** Department of Molecular Microbiology, School of Biotechnology, Madurai Kamaraj University, Madurai, Tamil Nadu, India; University of Szeged, HUNGARY

## Abstract

Molecular morphometrics is an emerging third dimensional aspect of fungal species delimitation. They have been demonstrated to be more informative than conventional barcoding methods. Hence in this study, foliar endophytic fungal (FEF) assemblages in three Magnoliopsida plants were delimited using nuclear ribosomal internal transcribed spacer 2 (ITS2) sequence—secondary structural features based phylogenetic analysis, also known as molecular morphometrics. A total of 392 FEF isolates were obtained from the *Aglaia elaeagnoidea*, *Flacourtia inermis*, and *Premna serratifolia* leaves and grouped into 98 morphotypes. Among these host plants, *P*. *serratifolia* showed the maximum percentage of colonization frequency. Representatives of each morphotype was sequenced and subjected to further molecular characterization. The results revealed that morphotypes were belonged to the phylum of Ascomycota, distributed over two classes (Sordariomycetes (68.59%) and Dothideomycetes (31.41%)), 6 orders and 19 genera. Based on compensatory base changes (CBC) analysis and absolute identity of ITS2 structure, 21, 20 and 23 species were recognized from *A*. *elaeagnoidea*, *F*. *inermis*, and *P*. *serratifolia* respectively. Diversity indices were higher in *A*. *elaeagnoidea*, despite it accounted for a modest 16.8% of total isolates recorded in this study. The genus *Colletotrichum* was predominant in *A*. *elaeagnoidea* (39%) and *P*. *serratifolia* (48%). Similarly, *Diaporthe* (43%) was dominant in *F*. *inermis*. Several host-specific species were also observed. This study concludes that these plants host diverse species of Ascomycota. To the best of our knowledge, this is the first detailed report on FEF diversity from these plants. Also, the inclusion of ITS2 secondary structure information along with the sequence provides a further dimension to resolve the inherent problems in identification of fungal species.

## Introduction

Foliar fungal endophytes are asymptomatic inhabitants of healthy plant leaves for all or part of their life cycle [1–3]. Such co-evolution of endophytic fungi and host likely existed when plants colonized land (since 400MYr), thus contributing a long and significant role in steering the evolution of fungal life on earth [4,5]. Endophytes, transmitted from one generation to another through infected host tissues (seeds, vegetative propagules) or fungal spores [5,6], may confer the host with physiological/fitness benefits over its competitors [7]. They have been proven as a fascinating source of structurally novel and biologically active secondary metabolites [8–12]. Also, the fungal endophytes have been catalogued to produce plant bioactive compounds and their analogs, thus making them a promising source of novel compounds with great potentials in medicinal, agricultural and industrial arenas [12,13]. Scientific communities also have renewed their interests in bio-prospecting these microorganisms to avoid large-scale harvesting of medicinal plants [12,14].

Almost all plant species serve as a repository for one or more endophytic organisms [15–17]. Investigation of endophytic assemblages in a plant over space and time is a laborious process which in fact is complicated with the intricacies of fungal systematics and phylogenetics [18]. The morphological and reproductive characters are predominantly subjective and fall short of providing unequivocal species delimitations. Hence, the molecular data are embraced in fungal systematics and phylogenetics [19]. Several genetic markers are in use for molecular identification of fungi. Among the molecular markers used in fungal identification, nuclear ribosomal internal transcribed spacer (ITS) region has been the most widely used marker and is the formal fungal DNA barcode. The majority of the earlier reports reveal that ITS outperforms other genetic markers in phylogenetic reconstruction of diverse eukaryotes [20–23]. It provides better discrimination of closely related species, besides offering higher success rates in PCR and sequencing experiments [19,24]. The molecular identification is often achieved through similarity searches among archived sequence data from public databases; however, caution has to be excised to avoid false identification [15,25,26]. The erroneous—incomplete entries in public databases and lack of reliable public reference data set have been the major setbacks in molecular identification of fungi [27,28]. Recent progresses in addressing the above issues look promising and have resulted in well-curated databases like UNITE (https://unite.ut.ee/search.php#fndtn-panel2) and Q-Bank (http://www.q-bank.eu/fungi/) [29,30]. However, developing a reference database encompassing taxonomically well-distinguished sequences of all reported fungi is yet to achieve.

In recent years, a substantial number of studies have demonstrated the ITS2 molecular morphometrics approach to produce significant vital phylogenetic information for delimiting the eukaryotes at different taxonomic levels, especially at genus and species ranks [31–35]. ITS2, a sub-region of ITS genetic marker, lies between the 5.8S and 28S genes of tandemly repeated rRNA genes. The ITS2 region is more conserved than the ITS1 region and hence, the ITS2 secondary structure information has been widely employed in species diagnosis [20,36–38]. The amalgamation of the ITS2 sequence and secondary structure information has been shown to enhance the robustness of the phylogenetic inference [39]. The use of ITS2 sequence—secondary structural features (structural pattern, CBCs, insertion and deletion events (INDELs) and transition (Ts) / transversion (Tv)) for species delimitation is known as molecular morphometrics [31]. These features provide further dimension in the delimitation of organisms at the species level. For instance, compensatory base changes (CBCs, nucleotide substitutional events occurring at either side of a base pair) in ITS2 secondary structure has been presented as a possible diagnostic marker to predict the minimal number of different species present in datasets [37,40].

In this present study, we intended to investigate the FEF assemblages from three Magnoliopsida plants (*Aglaia elaeagnoidea* (A. Juss.) Benth., *Flacourtia inermis* Roxb., and *Premna serratifolia* L.) based on ITS2 molecular morphometrics analysis. FEF assemblages in these plants were not reported erstwhile and hence these medicinal plants were chosen. The foliar endophytic fungal isolates were delimited based on morphological characteristics along with their ITS sequence based phylogenetic analysis. Further, species identity was evaluated using ITS2 molecular morphometric analysis.

## Materials and methods

### Sample collection

**Fig 1:**
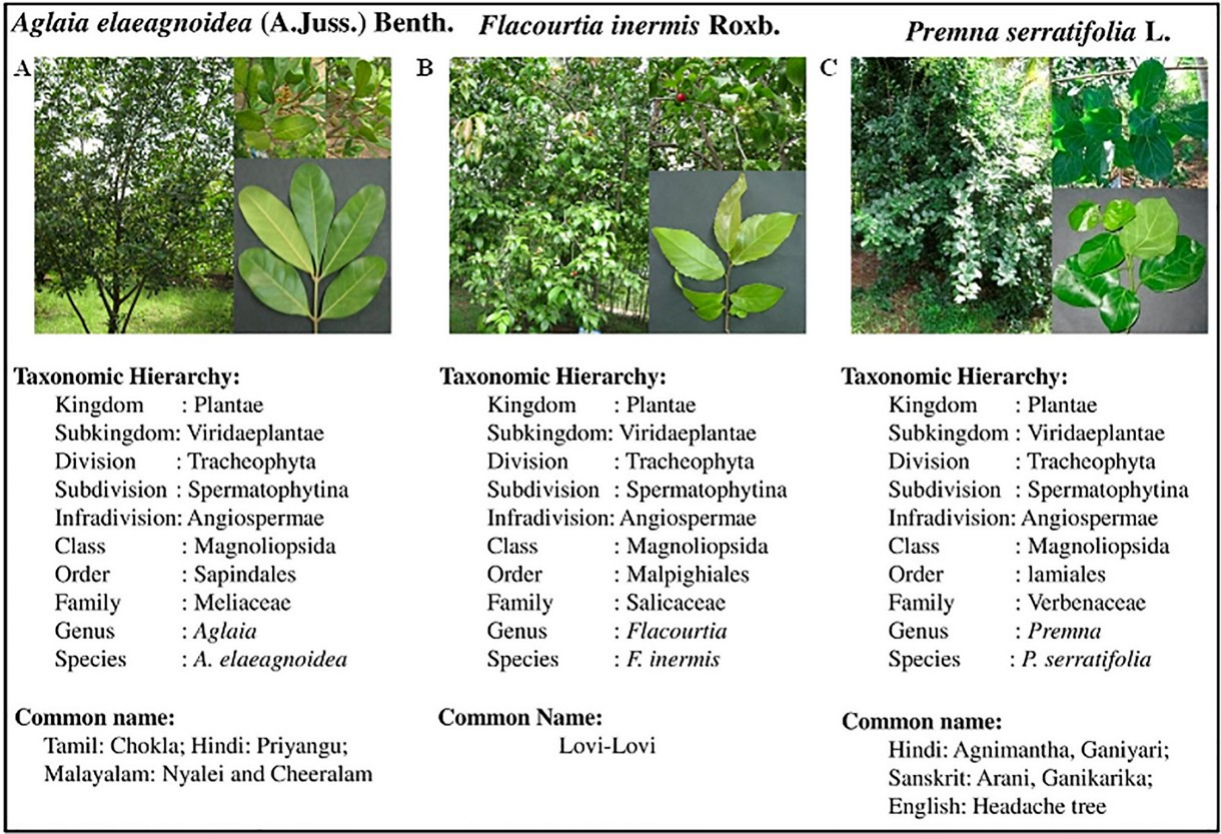
Taxonomical hierarchy of host plants: (a) *A*. *elaeagnoidea*, (b) *F*. *inermis* and (c) *P*. *serratifolia*.

Leaf samples were collected twice during the period of November 2014 to February 2015 from the plants growing in the ABS Botanical Garden, located in Kaaripatti, Salem, Tamil Nadu, India (Altitude: 366 meters above MSL, Longitude: 77.5E and Latitude: 12N) with appropriate permission (Auth. No.: AUT/MKU/067). Sixty healthy, mature leaves were collected from five plants of each hosts namely, *Aglaia elaeagnoidea* (A.Juss.) Benth., *Flacourtia inermis* Roxb. and *Premna serratifolia* L. (Fig 1). Then, the leaves were kept in sterile polythene bags and brought to the laboratory in an icebox. The leaves were stored at 4°C and processed within 24 hours of collection.

The collected leaves were washed thoroughly in running tap water and air dried. Subsequently, they were washed twice with sterile distilled water and were surface sterilized by sequential washes (5 seconds in 70% ethanol, 90 seconds in 4% sodium hypochlorite and then washed thrice in sterile distilled water (1 minute for each)) to remove the epiphytic microbes. After surface sterilization, the leaves were blotted on sterile tissue paper under laminar airflow and cut into smaller segments (0.5 X 0.5 cm^2^) with a sterile surgical blade and plated on culture medium to isolate the foliar endophytic fungi. The efficacy of the surface sterilization was tested by imprinting the sterilized leaf segments on the surface of potato dextrose agar (PDA) medium [41].

### Isolation and morphotyping

Fungal endophytes were isolated from 300 sterile leaf segments (consists of upper, middle and lower portion of the midrib and lamina region) of each host plant species. The leaf segments were placed on Petri plates (5 segments plate^-1^) containing PDA medium supplemented with streptomycin (250 mg L^-1^). The plates were incubated at 25 ± 2°C for 21 days under the light regimen of 12-hours white fluorescent light: 12-hours of dark cycle. The plant segments were monitored daily and emerging fungal hyphae were transferred to fresh PDA plates and incubated for a week. The rapidly grown isolates were removed with sterile scalpel following isolation, to avoid the inhibition of slow growing isolates. The pure cultures were transferred to PDA slants and maintained at 4°C [42,43]. The leaf imprint tests showed no growth, thereby validates the efficacy of the surface sterilization process employed.

The cultural characteristics such as colony growth rate, surface texture, color (adaxial and abaxial) and margin of the isolates were examined on the PDA plate. The isolates from each host were grouped into distinct morphotypes according to their morphological characters. The morphotypes obtained from the leaves of *A*. *elaeagnoidea*, *F*. *inermis* and *P*. *serratifolia* were labeled as Ae-01 to Ae-29, Fi-01 to Fi-32 and Ps-01 to Ps-37 respectively. One representative isolate of each morphotype was chosen for molecular characterization.

### Molecular characterization

Genomic DNA was extracted from 1 g of fungal mycelium scrapped out of 7 days old culture grown on PDA. The scrapped mycelium was pulverized with 4 ml of extraction buffer (400mM Tris-HCl (pH 8.0), 60mM EDTA, 150mM NaCl and 1% sodium dodecyl sulfate) using mortar and pestle [44]. 300 μl of 3M sodium acetate (pH 5.2) was added to the 600 μl of the pulverized sample and incubated at -20°C for 10 minutes. Subsequently centrifuged at 12,000 rpm for 5 minutes and to the supernatant, an equal volume of isopropanol was added and incubated at room temperature for 15 minutes to precipitate DNA and pelleted by centrifugation at 12,000 rpm for 3 minutes. The pelleted DNA was washed with 70% ice-cold ethanol by centrifuging at 12,000 rpm for 2 minutes [45]. Further, the pellet was air dried and re-suspended in sterile milli-Q water. The extracted DNA was checked by gel electrophoresis and stored at -20°C until further use.

The internal transcribed spacer (ITS) region of the nuclear ribosomal RNA gene was amplified with primers ITS1 (5'-TCCGTAGGTGAACCTGCGG-3') and ITS4 (5'–TCCTCCGCTTATTGATATGC-3’) [46]. The amplification was performed in a 25 μl reaction volume which consists, 2.5 μl of template DNA (10 ng/μl), 1.25 μl of each primer (10 pmol/μl), 12.5 μl of Taq DNA Polymerase Master Mix RED-Ampliqon (2X) and 7.5 μl of sterile milli-Q water. The amplification cycling program was as follows: 94°C for 4 minutes of initial denaturation, followed by 30 cycles of denaturation at 94°C for 30 seconds, primer annealing at 58°C for 1 minute and extension at 72°C for 2 minutes, and a final 7 minute extension at 72°C. A negative control was included in each amplification run (using sterile milli-Q water instead of template DNA). The amplified products were purified and sequenced (Sanger’s method) bi-directionally, at Eurofins genomics India Pvt Ltd., Bangalore. The ITS1 forward and ITS4 reverse complement sequences were merged using EMBOSS merger (http://emboss.bioinformatics.nl/cgi-bin/emboss/merger) to get full length of ITS region.

A total of 98 ITS sequences generated in this study, were blast searched against the sequences deposited in NCBI database. BLAST hits of closely related sequences were examined for greater query coverage, sequence similarity and lower e-value and were picked for further phylogenetic analysis. The identities of our isolates were analyzed through phylogenetic analysis, using sequences of authenticated cultures available in culture collection (CBS, NRRL, ATCC, and CPC) and/or curated sequences published in UNITE database, corresponding to the BLAST hit. The phylogenetic analysis was performed using Maximum Likelihood and Bayesian approaches. Multiple sequence alignment of the sequences was performed using the Clustal W program included in Mega 7 (https://www.megasoftware.net/) and the alignment was manually adjusted where necessary. The aligned sequences were exported and used for phylogenetic reconstruction.

RAxML v7.4.2 was employed for Maximum likelihood (ML) analysis with rapid bootstrap (BP) for 1,000 replicates using the GTR model [47]. The bootstrap support values from the ML analysis were presented in the tree. For the Bayesian analysis (BI), MrBayes 3.2.2 was run with 1,000,000 generations starting with a random tree. Every 100^th^ tree was saved into a file. The first 100,000 generations were deleted as the “burn-in” of the chain. The program Tracer v1.6 was applied to compare splits frequencies in the different runs and to plot cumulative split frequencies to ensure that stationarity was reached. Of the remaining trees, a majority rule consensus tree with average branch lengths was calculated using the sumt option of MrBayes [48]. Posterior Probabilities (PP) obtained for each clade was represented in the tree. The reconstructed trees were visualized using the TreeGraph ver.2 (http://treegraph.bioinfweb.info/Download).

### Molecular morphometric analysis

The ITS2 regions were extracted from ITS sequence by using Fungal ITS extractor. It is available at http://www.emerencia.org/FungalITSextractor.html [49]. Then, 20 bases of 5.8S and 28S rDNA nucleotide bases were added respectively to the 5’ and 3’ end of the extracted ITS2 region [50]. The folding of ITS2 region was performed using the Mfold ver. 3.1 web server (http://unafold.rna.albany.edu/?q=mfold/rna-folding-form) with default condition (Folding temperature: 37°C, ionic conditions: 1M NaCl (no divalent ions), percent sub-optimality number: 5, upper bound on the number of computed folding: 50, maximum interior/bulge loop size: 30, maximum asymmetry of an interior/bulge loop: 30 and maximum distance between paired bases: no limit) [51]. Among all the possible predicted structures, the one with minimal free energy and conserved helix pattern of eukaryotic ITS2 was chosen. The selected secondary structures were downloaded in Vienna format (containing sequence and secondary structure information) for constructing sequence and secondary structure based alignment.

The nuclear ribosomal ITS2 sequences were aligned along with their secondary structures using 4SALE 1.7. The alignment (sequence with secondary structure information and single letter amino acid codes of sequence secondary structure information were exported for NJ and ML analyses respectively) was exported for sequence–secondary structure based phylogenetic analysis. The CBCs were analyzed using CBCAnalyzer implemented in 4SALE. 4SALE was also used to visualize the consensus structure with sequence and secondary structure alignment information [37,52].

The alignment file obtained from 4SALE was imported to construct sequence-secondary structure based phylogenetic tree. The Neighbour-joining (NJ) tree was constructed using general time reversible (GTR) substitution model and an ITS2-specific scoring matrix as implemented in ProfDists with 1,000 pseudo-replicates [53]. The maximum likelihood tree was calculated using phangorn with 1,000 pseudo-replicates as implemented in the statistical framework R 3.4.1. The R script was available from the 4SALE homepage at http://4sale.bioapps.biozentrum.uni-wuerzburg.de [54].

### Analysis of diversity indices

The foliar endophytic fungal isolates from each host plant were analyzed on the basis of colonization frequency (CF): the number of species isolated were divided by number of segments screened x 100; relative percentage of occurrence (RPO) of different groups of fungi: density of colonization of one group divided by total density of colonization x 100 and endophytic infection rate (EIR): the number of infected segments divided by the total number of segments screened x 100 [55,56]. FUNGuild analysis was performed using online guilds application (http://www.stbates.org/guilds/app.php) to assess the functional roles of the species obtained in this study [57]. The species accumulation curves and diversity indices (Species richness (ACE and Chao 1), Species evenness (Simpson Inverse and Alpha), Shared species indices (Chao shared, Jaccard, Chao-Jaccard, and Bry-Curtis) and Shannon’s diversity index) were inferred with EstimateS 9.1.0 [58]. The data were represented using GraphPad PRISM 6.

## Results

**Fig 2:**
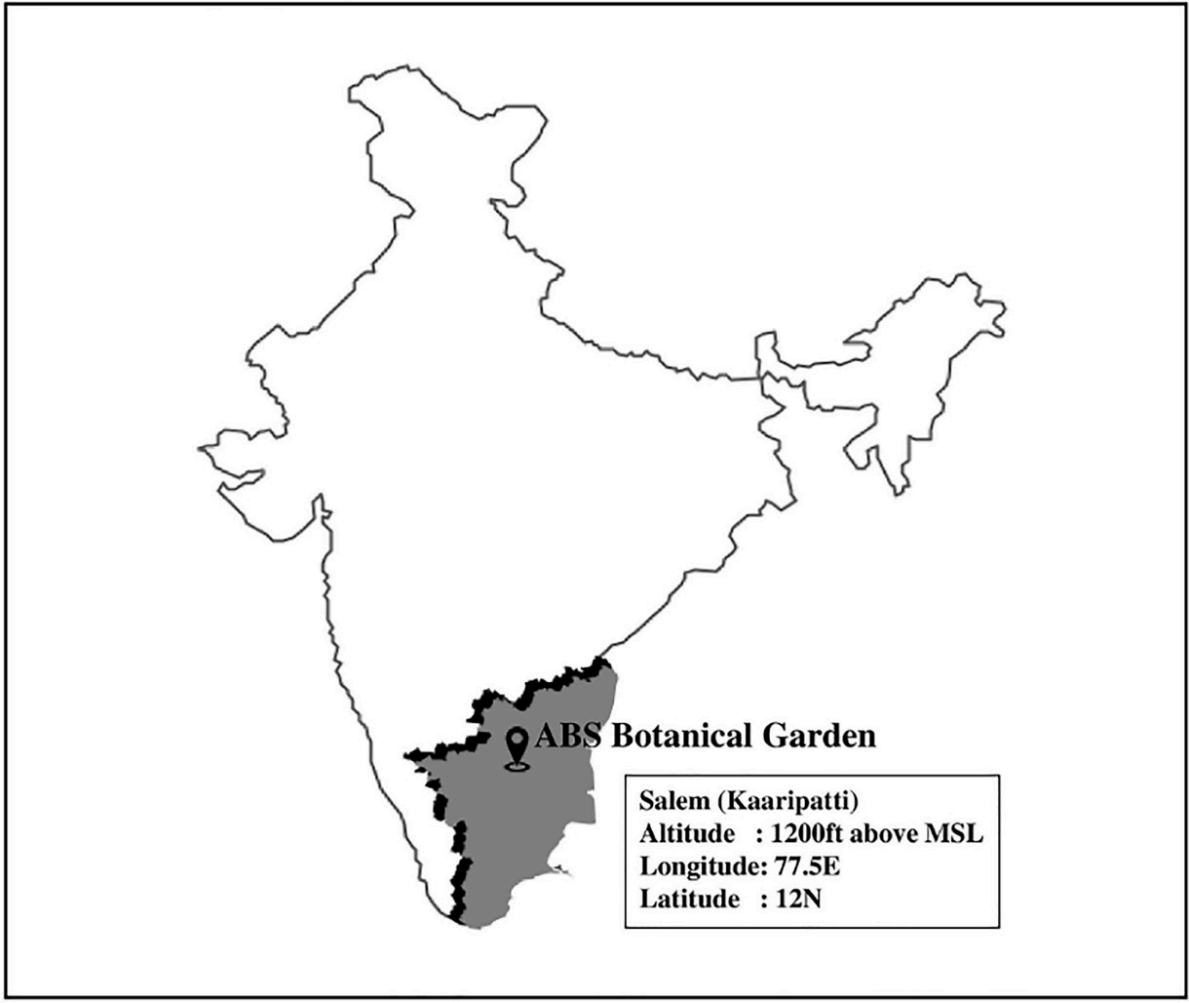
Map showing the geographical location of the sampling site.

A total of 392 foliar endophytic fungi were isolated and characterized from 900 segments of healthy leaf tissues of *Aglaia elaeagnoidea*, *Flacourtia inermis* and *Premna serratifolia* found in the ABS Botanical garden. These hosts and this geographical location (Fig 2) had not been explored erstwhile for FEF diversity.

### FEF distribution in three Magnoliopsida plant leaves

**Fig 3:**
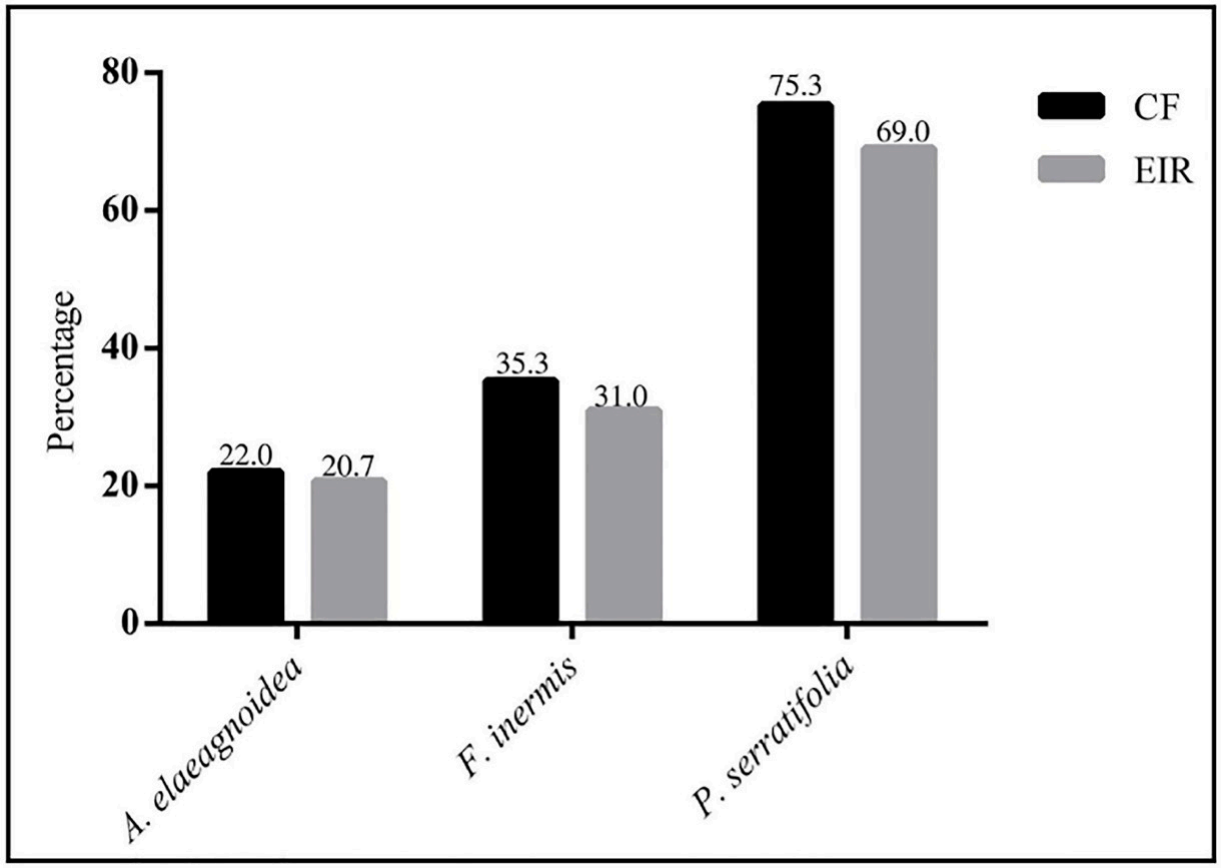
Colonization frequency (CF) and endophytic infection rate (EIR) of foliar endophytic fungi from three Magnoliopsida plants.

Out of 392 isolates recovered, 66 isolates were obtained from *A*. *elaeagnoidea*, while 105 and 221 isolates were recovered from *F*. *inermis* and *P*. *serratifolia* respectively. The sampling efforts and isolates recovered from the three Magnoliopsida plants are presented in Table 1. In the three plants investigated, midrib regions of the leaves harbored 0.64, 1.00 and 0.43 fold more isolates than the isolates recovered from the lamina of *A*. *elaeagnoidea*, *F*. *inermis* and *P*. *serratifolia* respectively. Out of the 300 leaf segments screened from each plant species, interestingly no endophyte was isolated from 236, 208 and 101 leaf segments of *A*. *elaeagnoidea*, *F*. *inermis* and *P*. *serratifolia* respectively. The EIR of *A*. *elaeagnoidea*, *F*. *inermis* and *P*. *serratifolia* leaves were 21.3%, 30.7%, and 63.3% respectively. This implies that the leaves of *P*. *serratifolia* were highly colonized (CF 73.7%) while the *A*. *elaeagnoidea* were the least colonized (CF 22.0%) among the three plants investigated and *F*. *inermis* having a CF of 35.0%. The EIR and CF of these host plants are shown in Fig 3.

**Table 1:** Sampling effort and number of foliar endophytic fungi isolated from *A*. *elaeagnoidea*, *F*. *inermis*, and *P*. *serratifolia*.

Plant	Sampling events	No. of leaves	Leaf region	No. of segments	No. of segments infected	No. of isolates	Total No. of isolates
*Aglaia elaeagnoidea* (A.Juss.) Benth.	I	30	Mid rib	75	15	17	66
			Lamina	75	08	08	
	II	30	Mid rib	75	24	24	
			Lamina	75	17	17	
*Flacourtia inermis* Roxb.	I	30	Mid rib	75	24	31	105
			Lamina	75	12	13	
	II	30	Mid rib	75	34	39	
			Lamina	75	22	22	
*Premna serratifolia* L.	I	30	Mid rib	75	61	69	221
			Lamina	75	42	44	
	II	30	Mid rib	75	51	61	
			Lamina	75	45	47	

### Morphotyping of FEF

Based on the cultural characteristics such as growth rate, colony morphology, hyphal characters, spores morphology and pigmentation, the fungal isolates were grouped into distinct morphotypes. In the present study, large numbers of sterile forms (66%) were obtained and they were categorized predominantly based on cultural characteristics (i.e. colony color, surface texture, growth pattern, margin and growth rate). Consequently, 29, 32 and 37 distinct morphotypes were recognized from *A*. *elaeagnoidea*, *F*. *inermis* and *P*. *serratifolia* respectively (Fig A in S1 File). Totally, 98 morphotypes were recognized in these plants, of which 66.3% were plurals (represented by more than one isolate), and the remaining morphotypes were singletons (represented by a single isolate). In *A*. *elaeagnoidea* and *P*. *serratifolia*, 72.41% and 78.38% of the morphotypes were plurals respectively, while in *F*. *inermis* only 46.88% of morphotypes belongs to plurals. Further, these morphotypes were delineated based on ITS2 molecular morphometrics.

### Taxonomic placement of FEF

**Fig 4:**
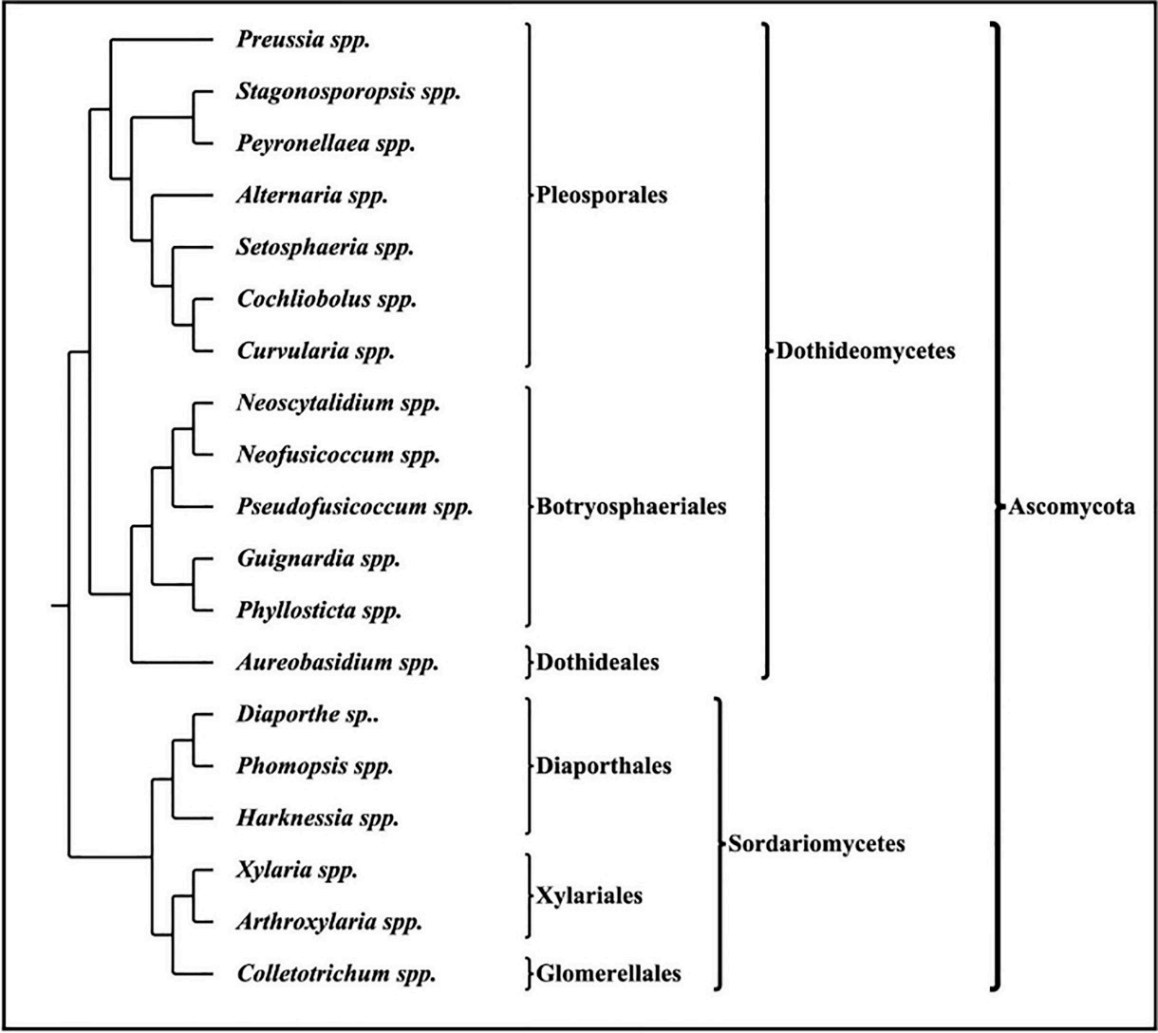
Taxonomical hierarchy of foliar endophytic fungi isolated from *A*. *elaeagnoidea*, *F*. *inermis* and *P*. *serratifolia*.

The identity of the representative isolates of each morphotype was ascertained based on the phylogenetic analysis of their ITS sequence with their closest and related reference sequences from global repositories. Phylogenetic analyses were performed using both Maximum Likelihood and Bayesian approaches for the entire dataset. Similar topologies were observed in both approaches, hence to avoid redundancy, ML tree is shown with BP and PP support values (Figs B–J in S1 File). Inferring from the phylogenetic results, all the isolates were found to stem from two classes (Soradariomycetes and Dothideomycetes) of the Ascomycota. Soradariomycetes accounted for 68.37% of isolates and were represented by 3 orders (Diaporthales, Xylariales and Glomerellales), 4 families and 6 genera. Dothideomycetes accounted for 31.63% of isolates and were represented by three orders, namely Botryosphaeriales, Dothideales and Pleosporales comprising 13 genera, belonging to 5 families (Fig 4). Interestingly greater diversity was recorded in Dothidiomycetes despite accounting for a lesser percentage of isolates.

A total of 19 genera namely, *Colletotrichum* (26 morphotypes), *Xylaria* (2 morphotypes), *Arthroxylaria* (1 morphotype), *Diaporthe* (28 morphotypes), *Phomopsis* (9 morphotypes), *Harknessia* (1 morphotype), *Setosphaeria* (4 morphotypes), *Alternaria* (10 morphotypes), *Guignardia* (2 morphotypes), *Aureobasidium* (1 morphotype), *Phyllosticta* (4 morphotypes), *Pseudofusicoccum* (1 morphotype), *Stagonosporopsis* (1 morphotype), *Preussia* (1 morphotype), *Neoscytalidium* (1 morphotype), *Peyronellaea* (1 morphotype), *Cochliobolus* (1 morphotype), *Curvularia* (1 morphotype), and *Neofusicoccum* (3 morphotypes) accounted for the 98 morphotypes recognized in this study. Interestingly in *A*. *elaeagnoidea*, 12 genera were recognized out of 29 morphotypes. However, in *F*. *inermis* and *P*. *serratifolia* with higher morphotypes (32 and 37), only 10 and 8 genera were recognized respectively.

FUNGuild analysis revealed that the isolates, belongs to five main trophic guilds (symbiotrophs–pathotrophs—saprotrophs, symbiotrophs—pathotrophs, symbiotrophs—saprotrophs, pathotrophs and sparotrophs). *Colletotrichum*, *Setosphaeria* and *Diaporthe* isolates were categorized as symbiotrophs—pathotrophs (74.0%). *Alternaria*, *Harknessia*, *Aureobasidium* and *Phyllosticta* isolates were belong to symbiotrophs–pathotrophs–saprotrophs (9.7%). *Guignardia*, Neofusicoccum, *Stagonosporopsis*, *Pseudofusicoccum*, *Neoscytalidium*, *Curvularia* and *Cochliobolus* were cetegorised as Pathotrophs (14.4%). *Arthroxylaria*, *Peyronellaea* and *Preussia* were categorised as saprotrophs (1.6%). Xylaria was assigned to saprotrophs–symbiotrophs (0.3%). The relative abundance of distribution of the isolates belonging to trophic guilds varied among the three hosts investigated in this study and are represented in Fig K in S1 File.

### Secondary structure prediction and analysis

**Fig 5:**
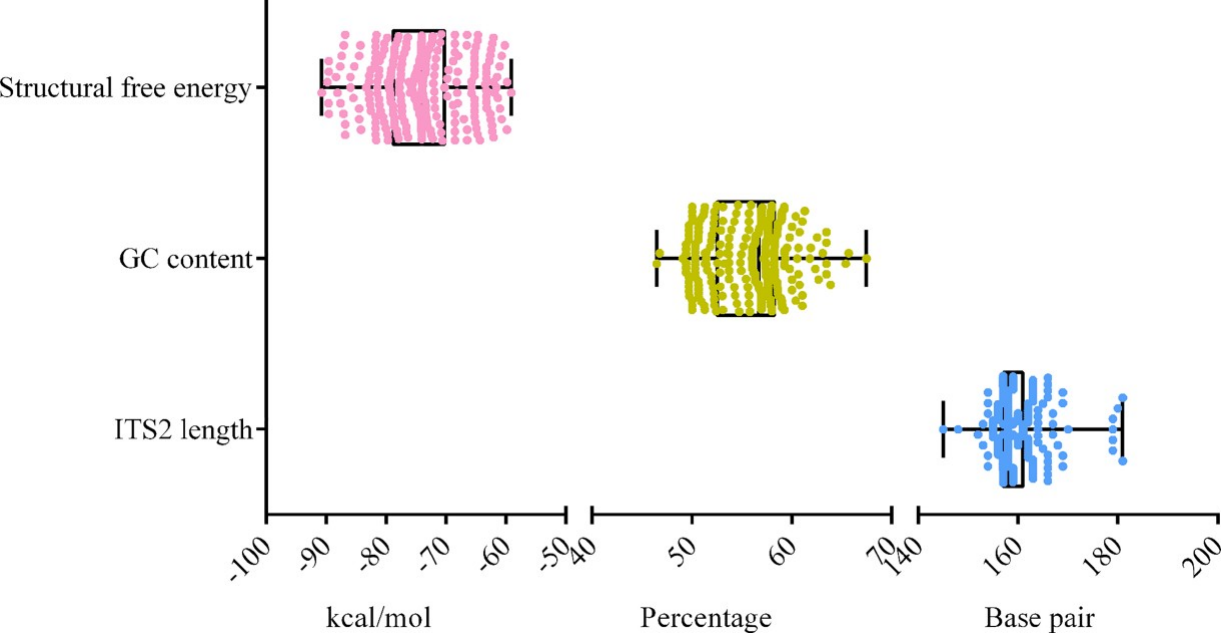
Box and whiskers plots depicting the secondary structral free energy, GC content and length of the ITS2 sequences employed in this study.

The ITS2 region was extracted from ITS sequences and they were used for phylogenetic reconstruction to evaluate the utility of this region in delimiting the fungal specis. The clade formations were similar in both ITS2 and full length of ITS region sequence tree. Hence, the closest reference sequences to our isolates were chosen for ITS2 molecular morphometric analysis. The length of the chosen ITS2 sequences of 98 query and 193 reference strains ranged from 145 bp (*Preussia minima* CBS 52450) to 181 bp (*Setosphaeria rostrata* Ae-06). The highest GC content was detected in *Phyllosticta maculate* CPC 18347 (67.46%) and the lowest in *Cochliobolus homomorphus* ATCC26651 (46.47%). The length and GC content of the ITS2 sequences analyzed in the data set are summarized in the nucleotide information table (Fig 5 and Table A in S2 File).

ITS2 secondary structures were predicted for a dataset comprising our query sequences and their phylogenetically closely related sequences. In total, secondary structures were predicted for 293 sequences and their structural minimum free energy is represented in Fig 5 and Table A in S2 File. The core structure of the ITS2 region has a central bulge with 3–4 helices radiating from it. These were designated as helices I-III or I-IV from 5’ to 3’ direction.

In this study, five distinct types of secondary structures were observed. In type 1, three helices protruded from the central bulge and the fourth helix does not occur. This type was predominantly observed in members of *Colletotrichum*, *Arthroxylaria*, *Peyronellaea*, *Harknessia*, *Preussia*, *Stagonosporopsis*, *Aureobasidium* and few species of *Diaporthe*, *Phomopsis* and *Xylaria*. In type 2, three helices were radiating from the central bulge and its extended subdomain. Helix I radiates from the central bulge, while helix II and III were radiating from the extended subdomain of the central bulge. Most species of *Diaporthe*, *Phomopsis*, and all the species of *Cochliobolus*, *Curvularia* and *Setosphaeria* investigated in this study possessed this type of secondary structure.

Type 3 structure shared similarities with the type 2 in possessing 3 helices radiating from the central bulge and its extended subdomain. Helix I radiates from the central bulge, while helix II and III were radiating from the extended sub domain of the central bulge. However, a small helix (2–3 canonical base pair with an apical bulge) was protruding between helix II and helix III. It was designated as helix IIa. Most species of *Alternaria* as well as *Phomopsis azadirachtae* possessed this type of secondary structure.

In type 4, four helices were observed to be radiating from the central bulge. This type of structure was observed in few species of *Neoscytalidium*, *Xylaria*, *Pseudofusicoccum* and *Colletotrichum*. In type 5, four helices were radiating from the central bulge and its subdomains. Helix III and IV were radiating from the central bulge and the helix I and II were radiating from the extended subdomain. Members of *Guignardia*, *Phyllosticta* and *Neofusicoccum* possessed this type of secondary structures.

**Fig 6:**
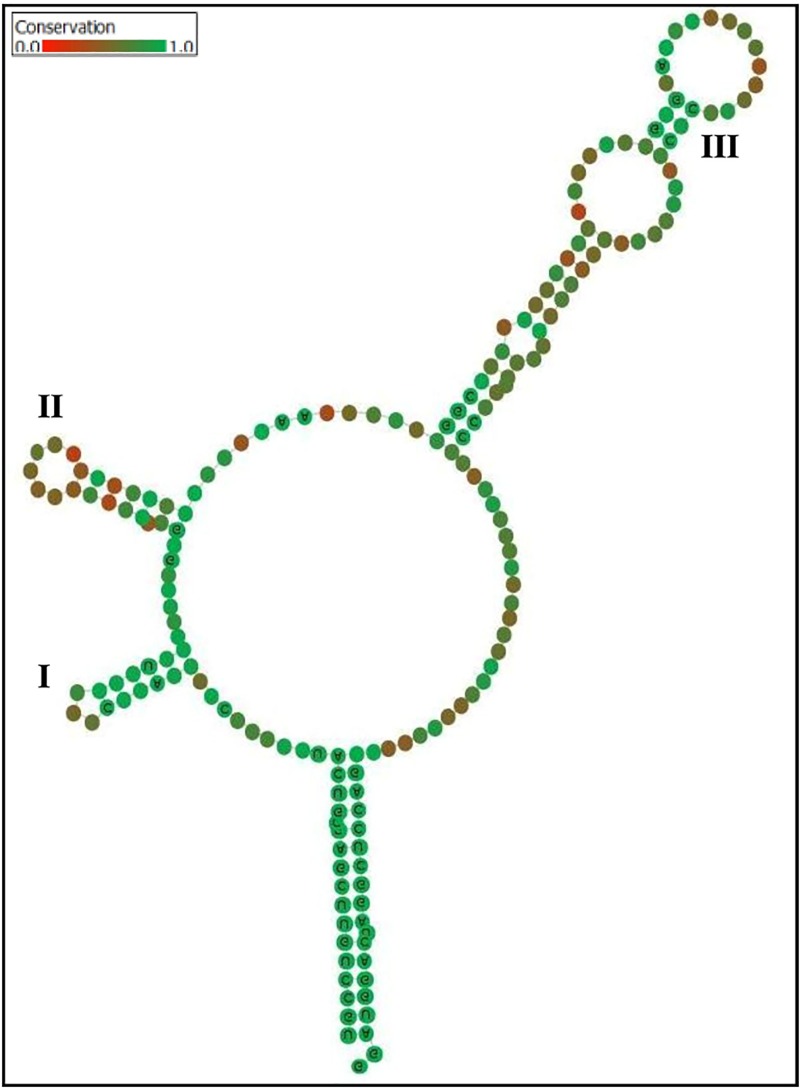
Consensus secondary structure of all internal transcribed spacer 2 (ITS2) sequences employed in molecular morphometric analysis. Helices were numbered I–III from 5’ to 3’ direction. Sequence conservation was indicated with colours from red (not conserved) to green (>51%; conserved).

The general consensus structure for all the 293 sequences modeled in this study possessed a three-helical pattern (Fig 6). Helix IV was highly variable and did not appear in several genera and henceforth it did not appear in the consensus structure. Helix III was the longest and contains a UGGC motif at its base. Exceptionally, in the members of Botryosphaeriales, Dothideales and *Diaporthe mayteni* a variant motif, CGGC was observed. Genus-wise consensus structures were also modeled and depicted in Fig L in S1 File. The regions of conservations and the intraspecies variations observed were depicted in color scale (red to green for variation to conservation). The sequence variations had implications in the structural variations, however, the common core of the predicted structures was concordant with the eukaryotic general rule of ITS2 secondary structure.

### Molecular morphometrics based species delimitation

Structural features, CBCs, substitutions and INDELs were investigated to delineate the species boundaries. CBCs were observed between the taxonomically characterized reference and query sequences, however, CBCs analyzed between the query sequences alone were presented for clarity (Tables A–C in S3 File). The presence of CBCs always indicated the distinctiveness of the species, thereby serving as a species delimiter as proclaimed in earlier reports. In this study, out of the 98 morphotypes, a minimum of 25 species were recognized based on CBC. More than one species were recognized in *Colletotrichum* (3 spp.), *Diaporthe* (3 spp.) and *Phomopsis* (3 spp.) and one species was recognized from each of the other genera reported in this study. Also, lack of CBCs was common among most of the species. Interestingly, CBCs were not found among both closely related and distinctly related species. But nucleotide variations were observed in the form of substitutions and INDELs. Henceforth, in addition to CBC, absolute structural identities were considered to delineate the morphotypes.

**Fig 7:**
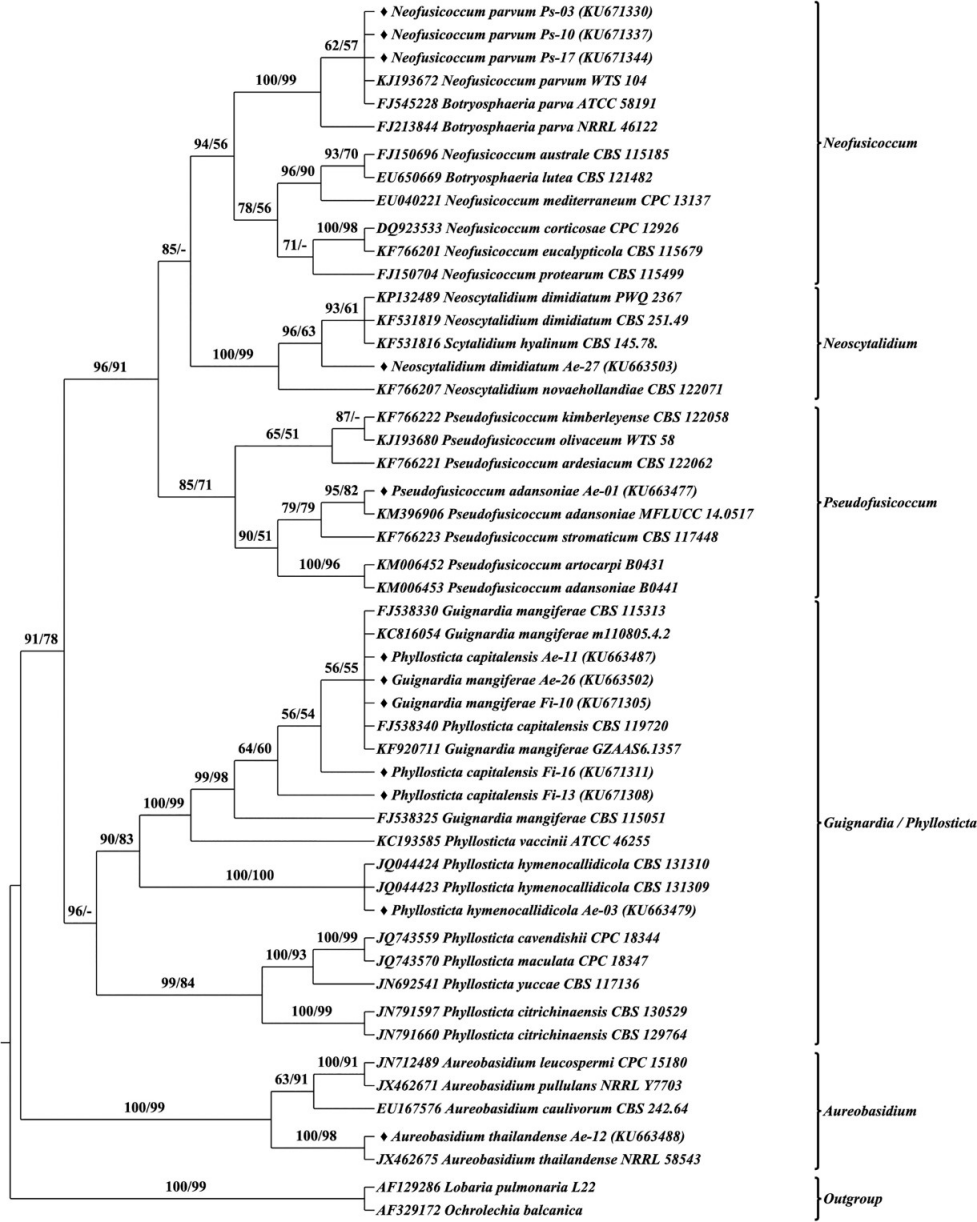
Internal transcribed spacer 2 sequence–secondary structure based phylogenetic tree of Botryosphaeriales and Dothideales query and reference sequences. The query sequences are represented by the symbol “♦”. Bootstrap (BP) values ≥50 were represented in the tree. Maximum Likelihood tree was presented with branch support values as BP of ML tree / BP of NJ tree.

**Fig 8:**
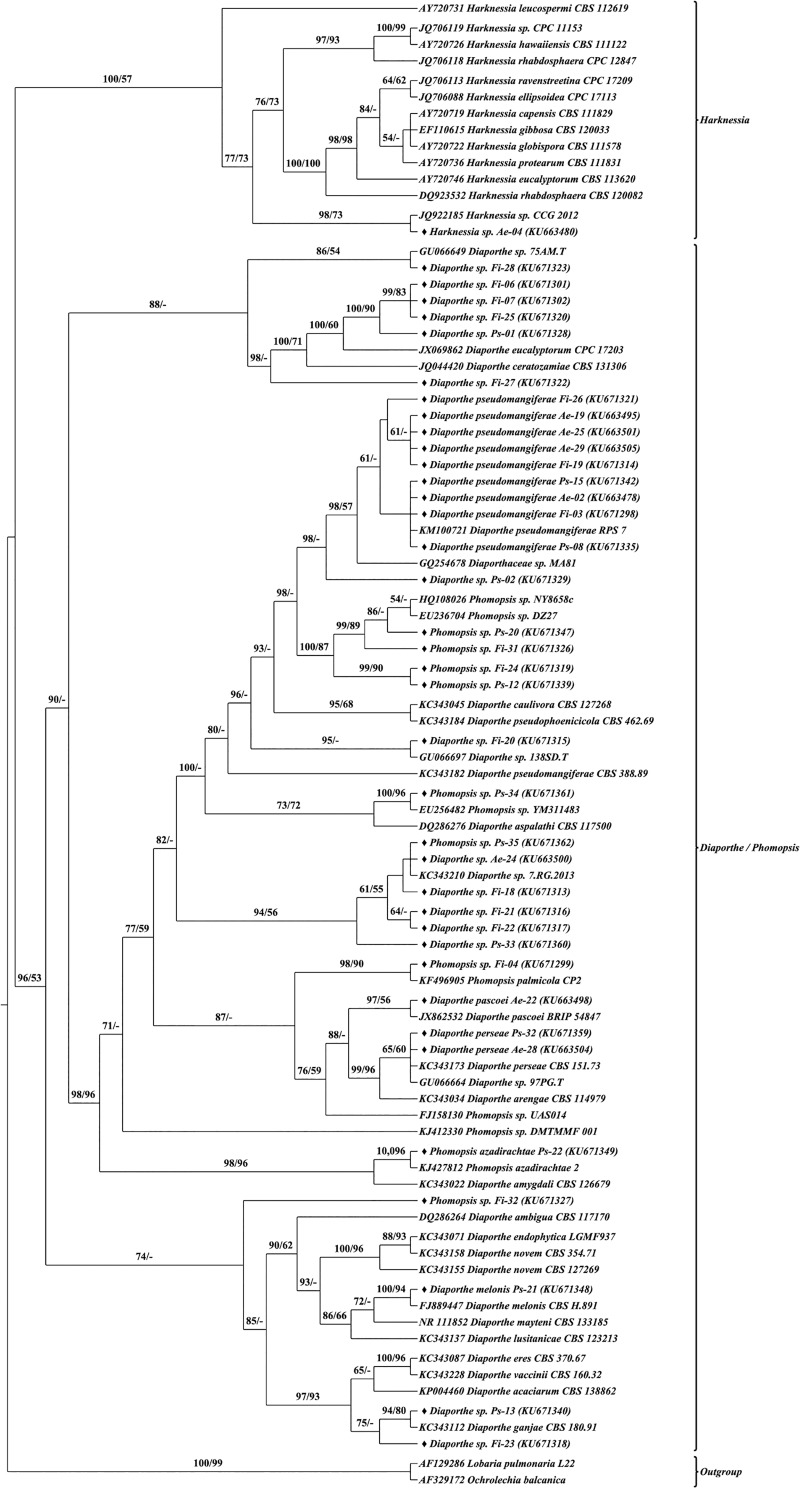
Internal transcribed spacer 2 sequence–secondary structure based phylogenetic tree of Diaporthales query and reference sequences. The query sequences are represented by the symbol “♦”. Bootstrap (BP) values ≥50 were represented in the tree. Maximum Likelihood tree was presented with branch support values as BP of ML tree / BP of NJ tree.

**Fig 9:**
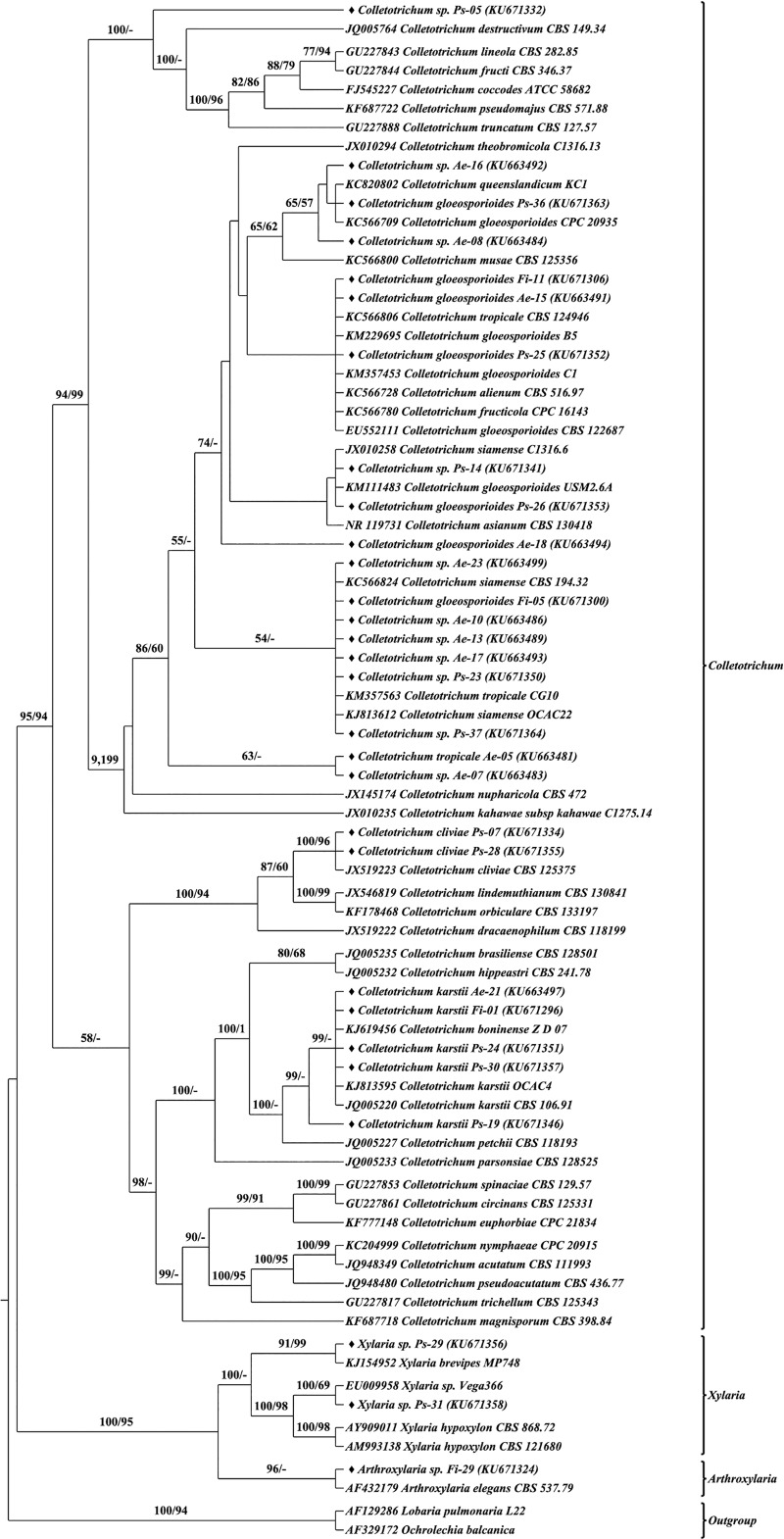
Internal transcribed spacer 2 sequence–secondary structure based phylogenetic tree of Glomerellales and Xylariales query and reference sequences. The query sequences are represented by the symbol “♦”. Bootstrap (BP) values ≥50 were represented in the tree. Maximum Likelihood tree was presented with branch support values as BP of ML tree / BP of NJ tree.

**Fig 10:**
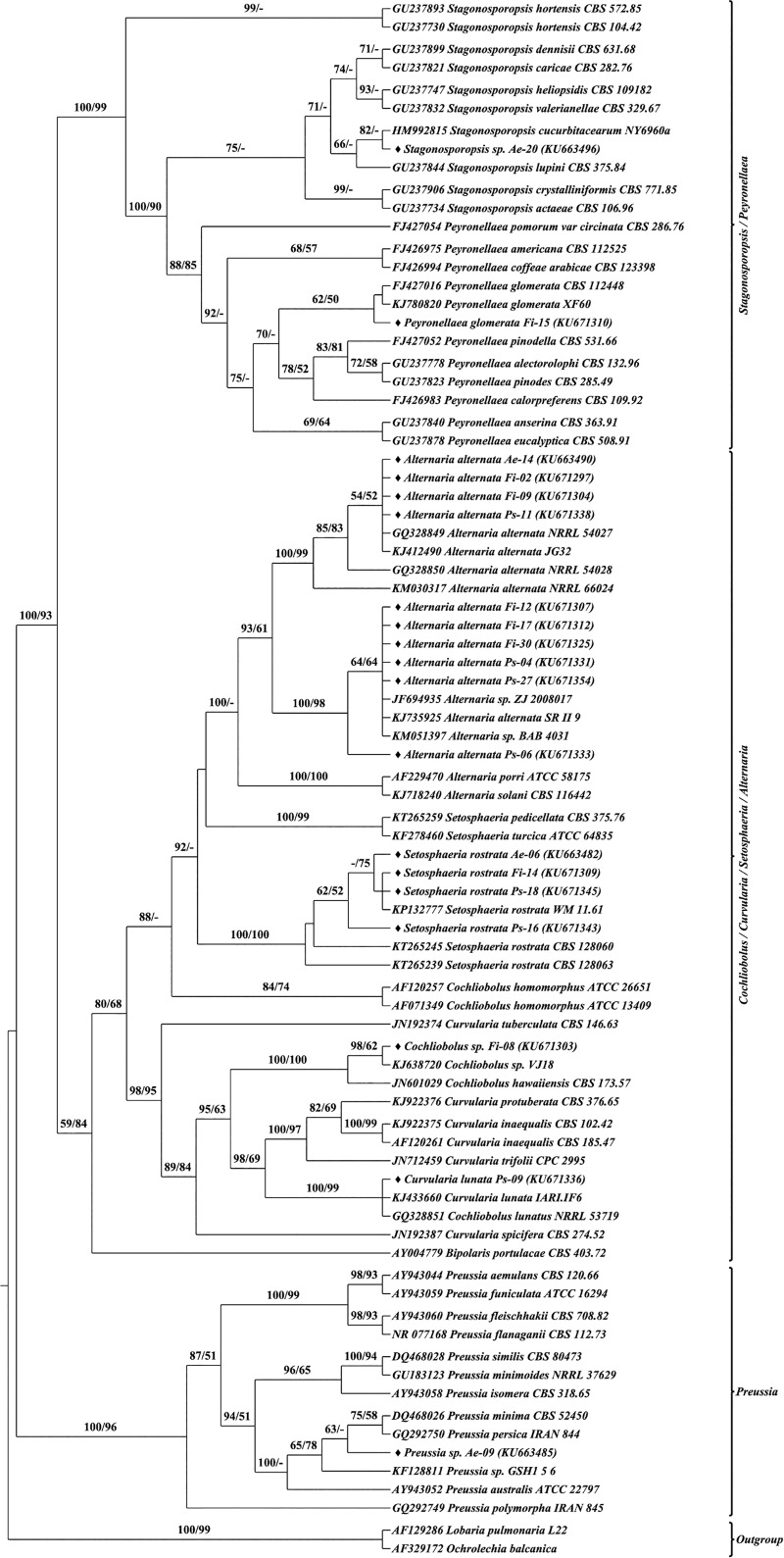
Internal transcribed spacer 2 sequence–secondary structure based phylogenetic tree of Pleosporales query and reference sequences. The query sequences are represented by the symbol “♦”. Bootstrap (BP) values ≥50 were represented in the tree. Maximum Likelihood tree was presented with branch support values as BP of ML tree / BP of NJ tree.

Sequence secondary structure based phylogenetic analysis was performed using Maximum likelihood and Neighbour-joining methods. Both methods generated similar tree topologies, therefore only the Maximum likelihood tree was presented with bootstrap values from both methods Figs 7–10. Single letter amino acid codes of nuclear ribosomal ITS2 sequence—secondary structure alignment information were submitted in TreeBASE under the accession number of 23593 (http://purl.org/phylo/treebase/phylows/study/TB2:S23593). Based on the molecular morphometric analysis, the 57 morphotypes were delimited to species level. The remaining 41 morphotypes were not delineated due to the lack CBCs and/or absolute structural identity with reference strains; hence they were designated in distinct groups (type-01, type-02, etc.) and considered as distinct taxonomical entities for diversity studies. Consequently, 21, 20 and 23 species were recognized from *A*. *elaeagnoidea*, *F*. *inermis*, and *P*. *serratifolia* respectively. For instance, *Phomopsis* sp. Ps-43 and *Harknessia* sp. Ae-04 rendered CBC against all the reference and query sequences of the respective genus and also lacked absolute structural identity. Therefore, they were not distinguished at the species level. These observations indicate that they may represent new species and warrants further investigation. After naming, the sequences were submitted in GenBank under the accession numbers KU663477 to KU663505 (29 morphotypes of *A*. *elaeagnoidea*), KU671296 to KU671327 (32 morphotypes of *F*. *inermis*) and KU671328 to KU671364 (37 morphotypes of *P*. *serratifolia*).

### Diversity of FEF in three Magnoliopsida plants

**Fig 11:**
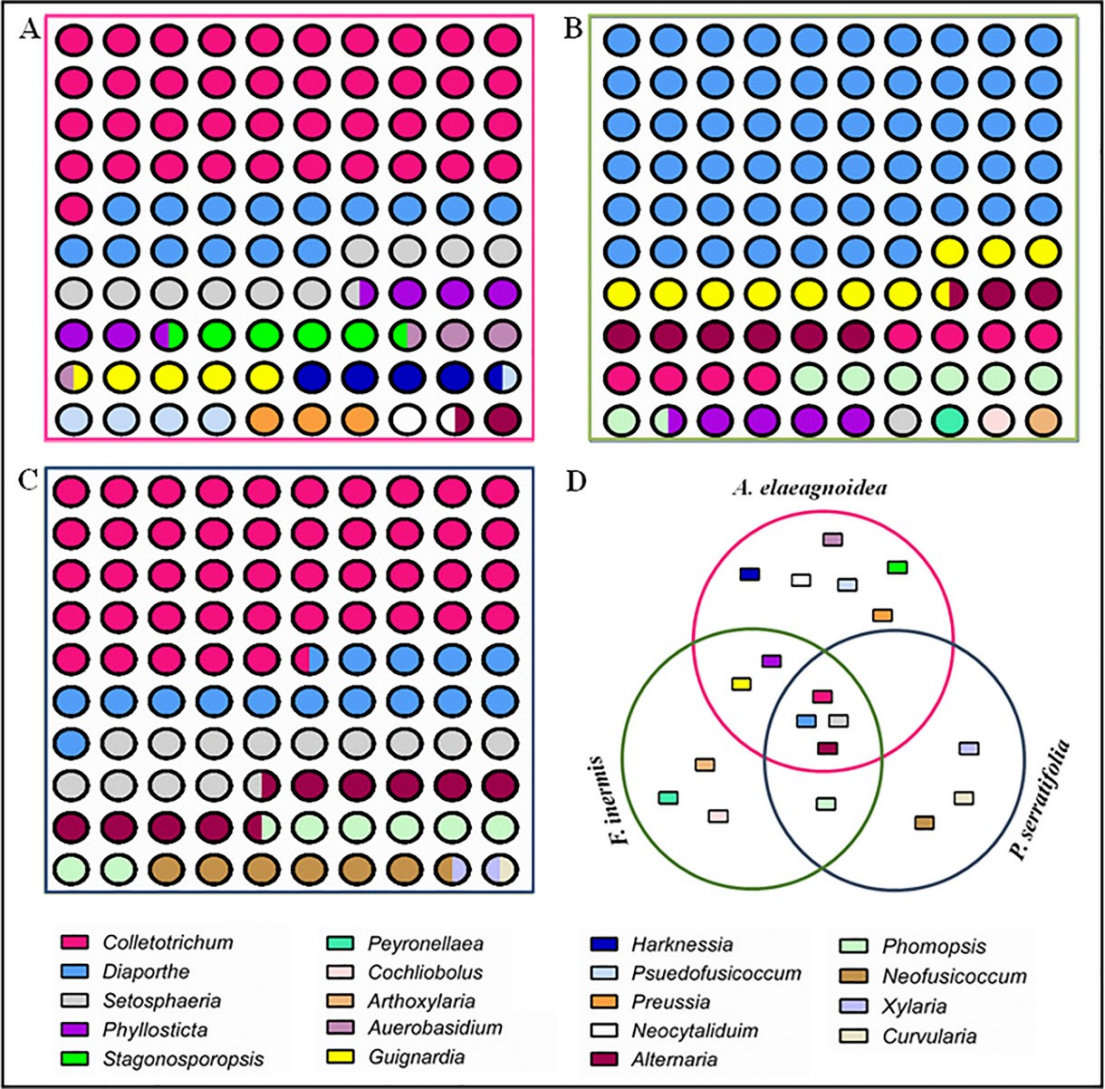
Relative percentage occurrence of different foliar endophytic fungal taxa isolated from **(A)** *A*. *elaeagniodea*, **(B)** *F*. *inermis* and **(C)** *P*. *serratifolia* and **(D)** Illustration of the host specificity.

At the genus level, diversity was higher in *A*. *elaeagnoidea* (12 genera), although the number of isolates recorded in it accounts for only 16.8% (29 morphotypes) of the total isolates. In case of *F*. *inermis* and *P*. *serratifolia*, 10 and 8 genera respectively were observed although the number of isolates recorded in them accounted for 26.8% (32 morphotypes) and 56.4% (37 morphotypes) respectively. Of the 19 genera recovered, *Setosphaeria*, *Alternaria*, *Colletotrichum* and *Diaporthe* were found in all plants. Members of *Guignardia* and *Phyllosticta* were found in both *A*. *elaeagnoidea* and *F*. *inermis*, while *Phomopsis* were distributed in both *F*. *inermis* and *P*. *serratifolia*. Interestingly, *Aureobasidium*, *Harknessia*, *Pseudofusicoccum*, *Stagonosporopsis*, *Preussia* and *Neoscytalidium* were found only in *A*. *elaeagnoidea*. Similarly, *Arthroxylaria*, *Peyronellae*, and *Cochliobolus* were found only in *F*. *inermis*. Likewise, *Xylaria*, *Neofusicoccum* and *Curvularia* were found only in *P*. *serratifolia*. The relative frequencies of these endophytes are shown in Fig 11 to express the diversity of FEF in the respective host plants.

**Fig 12:**
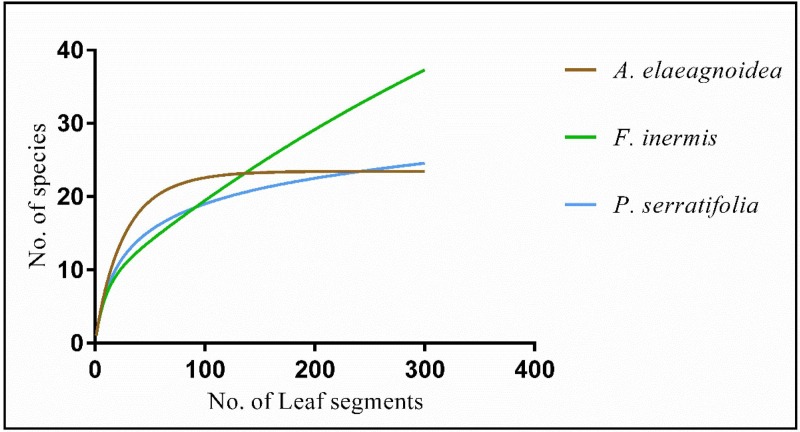
Species accumulation curves of foliar endophytic fungi recovered from Magnoliopsida plants.

Species accumulation curves and diversity indices of the FEF assemblages from the three plants were also assessed. The species accumulation curve did not reach an asymptote in *F*. *inermis* but it was approaching the asymptote in both *A*. *elaeagnoidea* and *P*. *serratifolia* (Fig 12). Estimated Shannon, Chao1, ACE, Simpson Inverse and Alpha indices are summarized in Table 2. Chao 1 and ACE predictions, revealed that the sampling effort was slightly less massive than would have been needed to account for the species richness of *A*. *elaeagnoidea* and *P*. *serratifolia*, while greater inadequacy was observed in sampling *F*. *inermis*. Shannon, alpha and Simpson inverse index were higher in *A*. *elaeagnoidea* than the other two plants investigated. The similarities of species assemblages across the three plants were described using Chao shared, Jaccard, Chao-Jaccard and Bray-Curtis indices (Table 3). The highest species similarity was observed between *A*. *elaeagnoidea* and *P*. *serratifolia*.

**Table 2:** Species richness (ACE and Chao 1), evenness (Simpson Inverse and Alpha) and Shannon’s diversity (H’) indices of *A*. *elaeagniodea*, *F*. *inermis*, and *P*. *serratifolia*.

Hosts	No. of isolates	No. of Morphotypes	No. of species	ACE	Chao 1	Shannon’s diversity index (H’)	Simpson Inverse	Alpha
*A*. *elaeagniodea*	66	29	21	22.64	22.64	2.79	12.81	10.63
*F*. *inermis*	105	32	20	47.24	47.24	2.41	8.68	7.33
*P*. *serratifolia*	221	37	23	26.32	26.32	2.62	10.56	6.46

**Table 3:** Shared species indices of *A*. *elaeagniodea*, *F*. *inermis* and *P*. *serratifolia*.

1^st^ sample	2^nd^ sample	Shared species	Chao shared	Jaccard	Chao-Jaccard	Bray-Curtis
*A*. *elaeagniodea*	*F*. *inermis*	6	12.04	0.171	0.328	0.199
*A*. *elaeagniodea*	*P*. *serratifolia*	8	8.14	0.222	0.420	0.237
*F*. *inermis*	*P*. *serratifolia*	6	7.00	0.162	0.282	0.184

**Fig 13:**
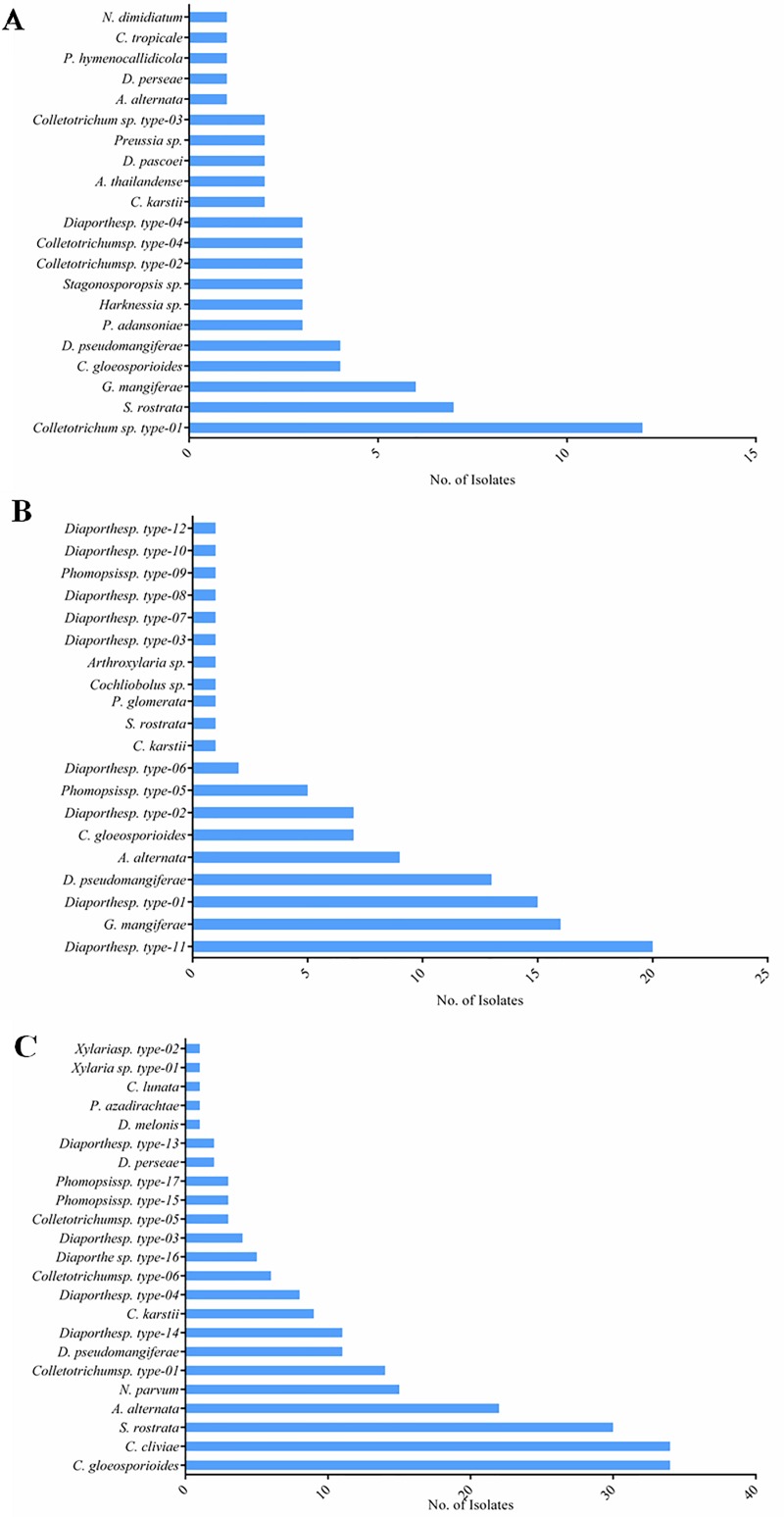
Ranked species graphs of foliar endophytic fungal assemblages in **(A)** *A*. *elaeagniodea*, **(B)** *F*. *inermis* and **(C)** *P*. *serratifolia*.

In *A*. *elaeagnoidea*, *Colletotrichum* sp. type-01, *S*. *rostrata*, *G*. *mangiferae*, *C*. *gloeosporioides* and *D*. *pseudomangiferae* were the dominant species. Similarly, in *F*. *inermis*, *Diaporthe* sp. type-11, *G*. *mangiferae*, *Diaporthe* sp. type-01, *D*. *pseudomangiferae*, *A*. *alternata*, *C*. *gloeosporioides* were the dominantly observed species. In *P*. *serratifolia*, *C*. *gloeosporioides*, *C*. *cliviae*, *S*. *rostrata*, *A*. *alternata*, *N*. *parvum*, *Colletotrichum* sp. type-01. *C*. *gloeosporioides* was predominantly recovered from all the three plants. Species distribution patterns varied greatly among the three plants investigated (Fig 13). Based on this study, it is evident that *A*. *elaeagnoidea*, *F*. *inermis*, and *P*. *serratifolia* host diverse foliar endophytic fungal species.

## Discussion

In the past four decades, numerous investigations have reported the FEF population to be abundant and multifarious in most plants [1,3,59–61]. The stomatal openings in the foliages offer passage to the entry of fungal propagules and diverse fungal lineages have been observed to colonise the extensive surface area of the foliar cover [62]. FEF have been touted as the reservoir of valuable metabolites and their bioprospecting could meet the pharmaceutical demands in a cost-effective, easily accessible and reproducible way [12,15,63]. Also, in several cases the medicinal property of compounds derived from these FEF resembles that of their host plants; hence they could potentially serve as an alternative source of the host metabolites [64,65].

The biotechnological exploitation of FEF necessitates the exploration and documentation of these organisms in the spatiotemporal regime [66]. Based upon this rationale, in the present investigation, leaves from three different Magnoliopsida plant (*A*. *elaeagnoidea*, *F*. *inermis*, and *P*. *serratifolia*) leaves were collected from the ABS botanical garden, one of the biodiversity rich site in Salem, TN and were investigated, to document the associated endophytic mycoflora. Diverse plant communities in the area influence the FEF diversity and several novel host specific species could be recovered from such hyper diverse regions [67]. Host specificity, tissue specificity, and various environmental factors (nutrient availability, rainfall, and interaction with external microbiomes) have been shown to impact the FEF diversity [59,68–72]. Hence this geographical location featuring greater biodiversity was chosen for the present investigation. Although all three plants have been traditionally used for medicinal purposes and the potential of their bioactive compounds has been reported in many studies [73–77], their FEF assemblages were unexplored and hence they were chosen for the present investigation.

A total of 66, 105 and 221 isolates were obtained from the *A*. *elaeagnoidea*, *F*. *inermis*, and *P*. *serratifolia* respectively. Also, higher numbers of isolates were recorded from the midrib region than from the lamina region, which is congruent with the previous reports [78,79]. The isolates from respective hosts were grouped into distinct morphotypes (29, 32 and 37 from Ae, Fi, and Ps respectively). Arnold et al. [80] reported 418 morphospecies representing 347 genetically distinct taxa from healthy leaves of *Heisteria concinna* and *Ouratea lucens*. The present study concurs with the earlier reports that all higher plants hosts endophytic microbes and the FEF composition varies among plants [1,67,81].

Representative isolates of each morphotype from respective hosts were identified based on the morphological and molecular morphometric analysis. As documented in erstwhile reports [82–86], most of our isolates were non-sporulating and exhibited pleomorphic morphological characters. The morphological descriptions have been marred with errors owing to the subjectivity and the documentation of new cryptic fungi with overlapping and pleomorphic features has blurred the species delineation [18]. We thus chose to use molecular and molecular morphometric analysis to delimit the representative FEF morphotype strains. Nonetheless, morphotyping has been useful to bin redundant cultures; removing them eases the study without adversely affecting the species richness estimates. Hence morphotyping have been employed with utmost caution in this study.

ITS has been accepted as the primary and standard barcoding marker of fungi, and it has been widely used to document the fungal diversity [22,87,88]. An explosive growth of the fungal sequences in public databases [80,89,90] is presently being observed owing to the technical advancement in sequencing and the relative ease of identification employing them. Despite the non-subjective nature (contrary to the morphological identification), phylogenetic utility and relative ease, molecular identification based on ITS markers is greatly impaired due to accumulation of erroneously named and unidentified ITS sequences in the public databases [15,25,26,86]. Based on these rationales, sequences of taxonomically well-resolved type strains or sequences of strains cataloged in culture collection center were employed as reference sequence set to avoid misnaming of the isolates.

The phylogenetic relationships of our isolates with the reference sequence set were assessed through ML and BI analysis. The tree topologies were almost congruent between these methods. Based on these analyses we could confirm that all the morphotypes belong to the phylum Ascomycota. Arnold et al. [1] and many other researchers have noted that all their isolates belonged to the phylum Ascomycota and that they were primarily distributed within the three classes, they were Dothideomycetes, Sordariomycetes, and Eurotiomycetes [70,91]. Likewise, in this study all the isolates were found to belong to the classes of Dothideomycetes and Sordariomycetes. A total of 19 genera were observed among the isolates recovered from the three plants explored in this study. The fungal genera identified in this present study have been erstwhile recorded as FEF [3,6,55,70,92–97] and as pathogens [3,98] in various host plants. A few genera were also reported as epiphytes [62]. Likewise, *Neofusicoccum* was earlier reported in angiosperms predominantly and occasionally in gymnosperms [95]. Based on the reports documented in guild database, assignments of functional attributes to the documented taxa were made possible through FUNGuild analysis. Guild assignments to fungal isolates, highlights their potential lifestyle transitions and their ecological relevance to the hosts and ecosystem at large [57]. The FUNGuild revealed that the endophytic fungal isolates recovered in this study were predominantly belonging to the symbiotrophs—pathotrophs and least in symbiotrophs—saprotrophs trophic guilds. Such plant-microbial interactions are context dependent, depending on the host plant, diversity, population dynamics of microbes, biotic and abiotic environment [99]. Based on the present study and earlier reports we concur that FEF is most abundant and multifarious among hosts.

In addition to the morphologically informative characters and molecular identification, nowadays researchers employ the ITS2 molecular morphometrics to minimize the error in species delimitation [36,37,40]. Molecular morphometrics is the third dimension of fungal species delimitation, which is also extensively used in the delimitation of other eukaryotic species. Several recent studies have proved that ITS2 molecular morphometric analysis could be effectively used to resolve eukaryotic organisms at the genus and species level. Ma et al. [33] reported that the application of secondary structure information provides a realistic picture of relationships within *Heterodera*. Chen et al. [100] and Han et al. [34] demonstrated the potential use of the ITS2 region as a standard DNA barcode to identify medicinal plants and their closely related species. In fungi, such studies are relatively sparse. Yan et al. [34] reported the ITS2 sequence-secondary structure based analysis performed better than the multi-marker based phylogeny in identifying endophytic *Pseudocercospora* fungi on poplars. Ahvenniemi et al. [101] suggested recognizing anastomosis groups of *Rhizoctonia solani* as distinct species based on the presence of CBCs among them. They also presented credible evidences for CBCs as a reliable species distinguisher (owing to genetic incompatibility) in cryptic species where sequence heterogeneity is common. Based on these rationales, ITS2 molecular morphometrics was employed in this study to delimit the FEF. The secondary structure of the ITS2 regions was predicted using the widely reported Mfold program with default conditions as described in erstwhile reports [33,102,103].

The ITS2 structure of the individual isolates consists three to four helices arranged along a central loop with the third as the longest [104–107]. Several studies have also noted that the number of helices in the ITS2 secondary structure may vary; helix IV, is absent in many groups of eukaryotes, however, helix II and III are recognizable in all [106,108]. Likewise, in the present study also we found the three helical patterns in most of the individuals and in the consensus structure of the whole dataset because helix IV was not recognized in several taxa. Sequence variations led to the differences in the folding pattern as a result of which five distinct patterns were recognized in this study. However the helical patterns of the structures observed were similar to those described for fungi, plant-parasitic nematodes and many other eukaryotes [33,103,109,110]. The ITS2 sequence-secondary structure based phylogenetic tree was highly congruent with the ITS2 sequence and the full length of ITS rDNA sequence based phylogenetic tree topology at the species level.

Besides the phylogeny, the CBC concept was also used as one of the molecular aspect to delineate the species in eukaryotes. Taxa differing by one or more CBCs were proved to be sexually incompatible [111–113]. Muller et al. [37] re-examined the hypothesis and statistically proved the CBCs as a potential predictor of the minimum number of species. A total of 25 species were delineated based on the CBCs recorded, all of which resolved well in the phylogeny. No CBCs could be observed between numerous species combinations. In the absence of CBCs, several nucleotide variations such as INDELs and substitution were observed. These variations could have possibly led to the non-canonical base pairing or structural incongruence among the comparisons, which may account for the lack of CBCs. Also, species-specific variations were observed in several cases, however, further investigations in this aspect are necessary to validate their utility in delineation. Therefore in the present study, the isolates were named based on absolute identity with the ITS2 secondary structure of reference strains. Isolates with non-absolute identity were delimited only up to genus level to avoid contributing to the erroneous entries in the public databases. Consequently, 41 morphotypes were resolved only at the genus level due to the lack of reference isolates with absolute structural identity. Interestingly, *Harknessia* sp. Ae-04 and *Phomopsis* sp. Ps-34 rendered CBC against all the sequences included in this study and also lacked absolute structural identity. Therefore, they were not distinguished at the species level. These observations indicate that they may represent new species, such that they warrant further investigation. Embracing molecular morphometrics along with the morphological and sequence analysis could provide a potential resolution to resolve the inherent problems in fungal identification.

Despite our study limited to the exploration of FEF diversity, interesting observations were made, such that they warrant further investigations. For instance, several isolates of *Colletotrichum gloeosporioides* complex did not yield CBCs among them but never failed to yield CBCs and resolve distinctly in phylogenetic trees when compared to the isolates from the *C*. *boninense* complex. These results shows that the ITS2 sequence-secondary structure based phylogeny corroborated well with CBC results and were congruent with earlier reports [26,114]. Sequence variations and inconsistencies in describing intraspecies has resulted in numerous new species with overlapping features and this has confounded the resolution of several *Colletotrichum* spp.; same has been the case in several other genera (*Diaporthe*, *Phomopsis*, etc.) [98,115]. Detailed investigations incorporating large datasets are needed to investigate and resolve these intricacies.

The colonization frequency and endophytic infection rate of FEF observed in the three Magnoliopsida plants were congruent with earlier reports [6,93,116]. In this study, relatively higher CF and EIR were observed in *P*. *serratifolia*. The larger surface of its leaves for inoculum capture could be one of the reasons for higher CF and EIR. RPO of *Colletotrichum* and *Diaporthe* were higher in all the three plants investigated and several reports have documented the broad host range of these genera [91,94]. *Colletotrichum* was observed as the most dominant genus in *A*. *elaeagnoidea* and *P*. *serratifolia*. Likewise, *Diaporthe* was the most dominant genus in *F*. *inermis*. As reported earlier [6,59,117], several host-specific and common genera (occur in more than one host) were also observed in this study.

The species accumulation curve for *F*. *inermis* did not reach an asymptote and such has been the case in several earlier reports [80,118]. The reason for it may be attributed to the hyperdiversity of fungal assemblage, their dynamic changes depending upon the environmental conditions and the insufficient sampling effort [118]. In *A*. *elaeagnoidea* and *P*. *serratifolia*, non-parametric estimates of species richness, Chao1, and ACE predicts that the species accumulation curves were approaching the asymptote. These non-parametric estimators of species richness have been widely accepted for predicting the minimum number of species in the assemblages [119,120]. The indices such as Shannon, Simpson, and Alpha accounted for the diversity of assemblages and have been part of most diversity studies [59,81,121]. The diversity indices were higher in *A*. *elaeagnoidea* than the other two plants investigated despite the fact that it accounted for only 16.8% (29 morphotypes) of the total isolates. Hence, the FEF assemblage of *A*. *elaeagnoidea* was ascertained to be more diverse than the assemblages of the other two plants investigated. Similarities between the species assemblages were demonstrated by the Chao shared, Jaccard, Chao-Jaccard and Bray-Curtis indices. Estimates of these indices concurred that the species composition of *A*. *elaeagnoidea* and *P*. *serratifolia* had higher similarity. Several biotic and abiotic factors could have contributed to the above observation. Host specificity was also observed among our isolates, and they contribute to the dissimilarity in the FEF assemblages in hosts.

In summary, 392 FEF isolates recovered from *A*. *elaeagnoidea*, *F*. *inermis* and *P*. *serratifolia* were grouped into 98 morphotypes based on the morphological characteristics. The representative morphotype strains obtained from these Magnoliopsida plants were delimited based on the ITS2 molecular morphometric analysis. Among the isolates, five species widely occurred in all three plants but several depicted host specificity. Based on these results we conclude that the host plants harbour diverse fungal endophytes. In addition to the exploration of fungal species diversity in these plants, in this study, the ITS2 molecular morphometric analysis was extensively used to delineate the FEF. Its potential in resolving fungal taxa has been demonstrated. Based on our observations, we hypothesize that incorporating the nucleotide variations (INDELs and substitution) along with the CBC concept could potentially increase the robustness of the molecular morphometrics to distinguish closely related species. However, detail investigation in this aspect is necessary to reveal their utility in molecular morphometrics study.

## Supporting information

S1 File Culture morphology (Fig A) of the morphotypes recognized; Internal transcribed spacer sequence based phylogenetic tree (Figs B–J) of the query and reference sequences; Relative abundance of FEF isolates distributed among the trophic guilds (Fig K) as deduced by analysis with FUNGuild and Consensus secondary structure (Fig L) of the foliar endophytic fungal genera.
(PDF)

S2 File Nucleotide base composition and structural free energy of sequences used in the molecular morphometric analysis. (PDF)

S3 File Summary of compensatory base changes detected among the morphotypes recovered in this study. (PDF)
