# Supplementary material for: Internal transcribed spacer 2 (ITS2) molecular morphometric analysis based species delimitation of foliar endophytic fungi from Aglaia elaeagnoidea, Flacourtia inermis and Premna serratifolia
Source: PLoS One. 2019 Apr 9;14(4):e0215024. doi: 10.1371/journal.pone.0215024 (PMC6456209; doi:10.1371/journal.pone.0215024)
Supplement: S1 File — Culture morphology (Fig A) of the morphotypes recognized; Internal transcribed spacer sequence based phylogenetic tree (Figs B–J) of the query and reference sequences; Relative abundance of FEF isolates distributed among the trophic guilds (Fig K) as deduced by analysis with FUNGuild and Consensus secondary structure (Fig L) of the foliar endophytic fungal genera. (PDF) [file pone.0215024.s001.pdf]

Supplementary File 1

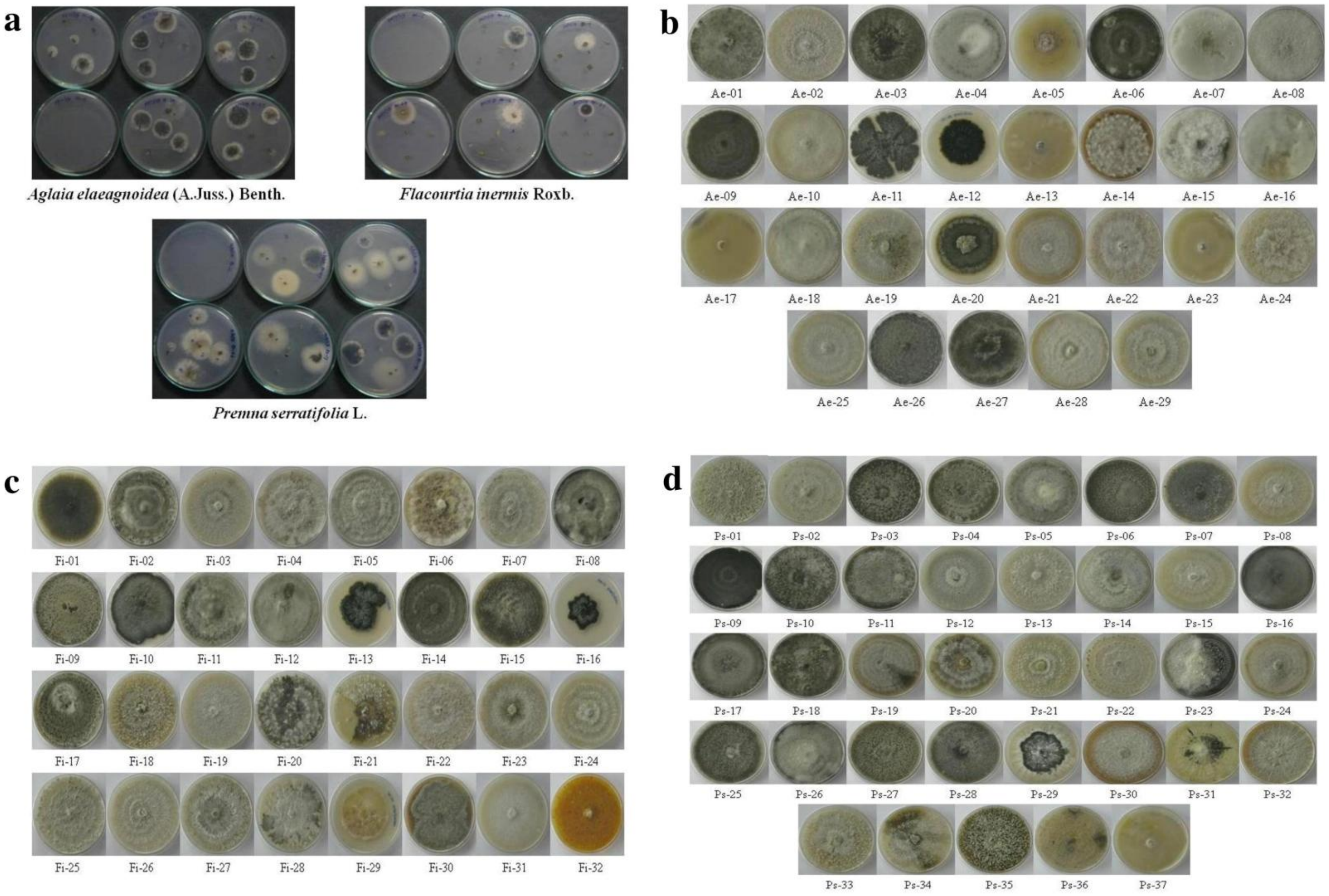

Fig A. Representative isolates of morphotypes recognized. (a) Emergence of foliar endophytic fungi from surface sterilized explants on potato dextrose agar plates. Culture morphology of the different morphotypes associated with the Magnoliopsida plants: (b) *A. elaeagniodea*, (c) *F. inermis* and (d) *P. serratifolia*.

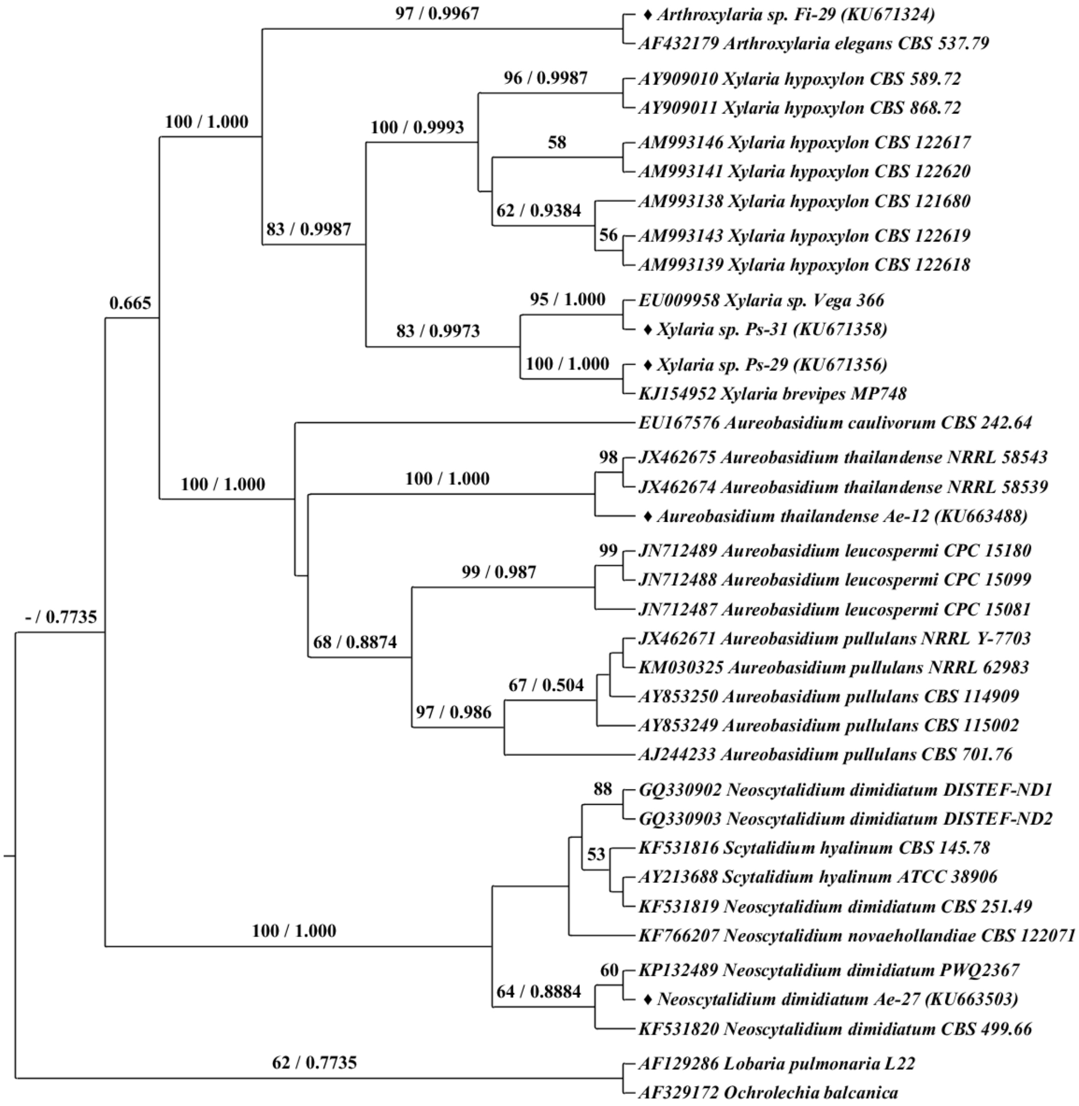

Fig B. ITS sequence based phylogenetic tree of *Arthroxylaria*, *Xylaria*, *Aureobasidium*, and *Neoscytalidium* query and reference sequences. *Lobaria pulmonaria* L22 and *Ochrolechia balcanica* sequences were used as an outgroup. The query sequences were represented by the symbol "♦". Bootstrap (BP) values ≥50 and posterior probability (PP) values ≥0.5 were represented above the branches of ML tree (BP/PP). The accession number of reference sequences was also given

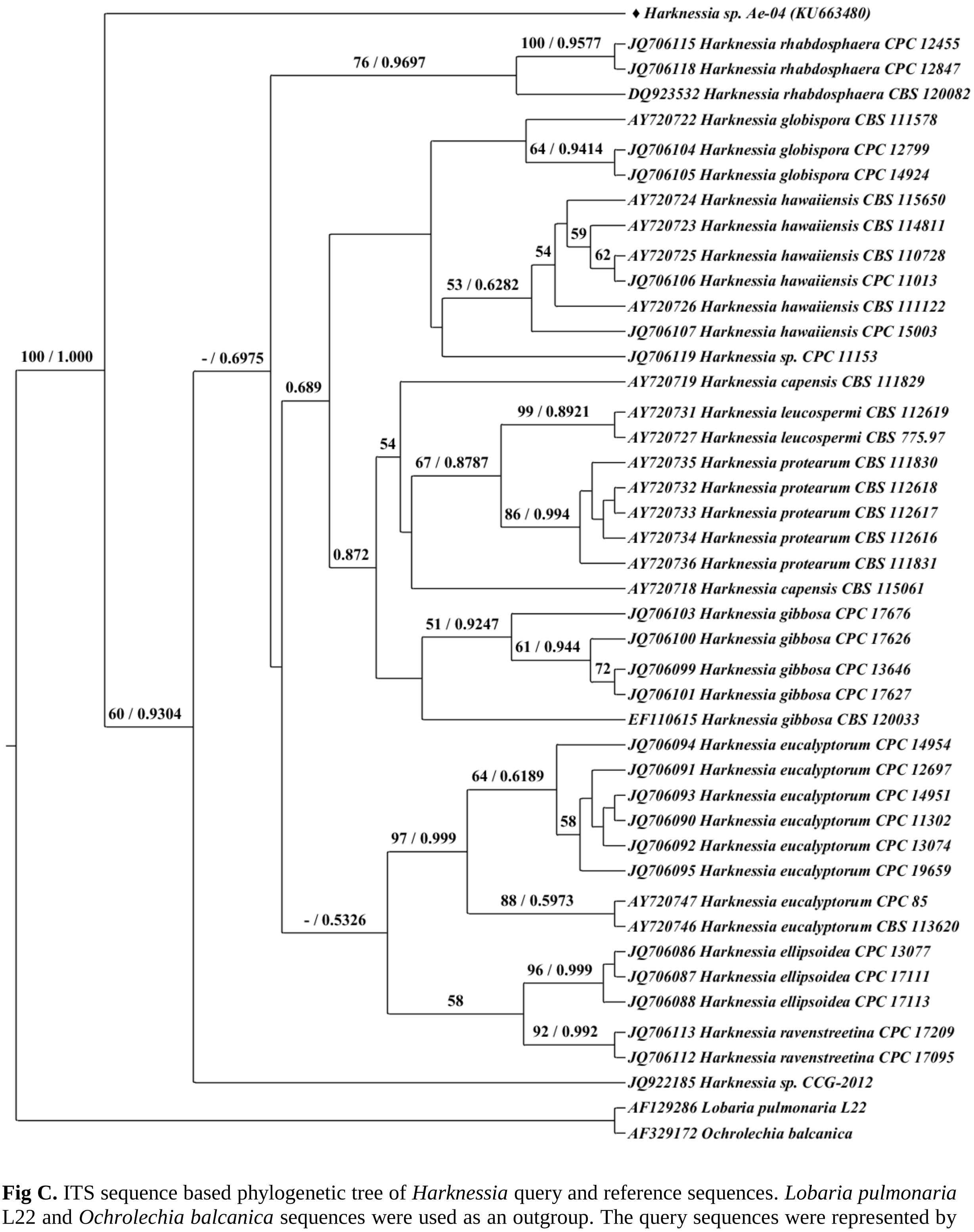

Fig C. ITS sequence based phylogenetic tree of *Harknessia* query and reference sequences. *Lobaria pulmonaria* L22 and *Ochrolechia balcanica* sequences were used as an outgroup. The query sequences were represented by the symbol "♦". Bootstrap (BP) values ≥50 and posterior probability (PP) values ≥0.5 were represented above the branches of ML tree (BP/PP). The accession number of reference sequences was also given

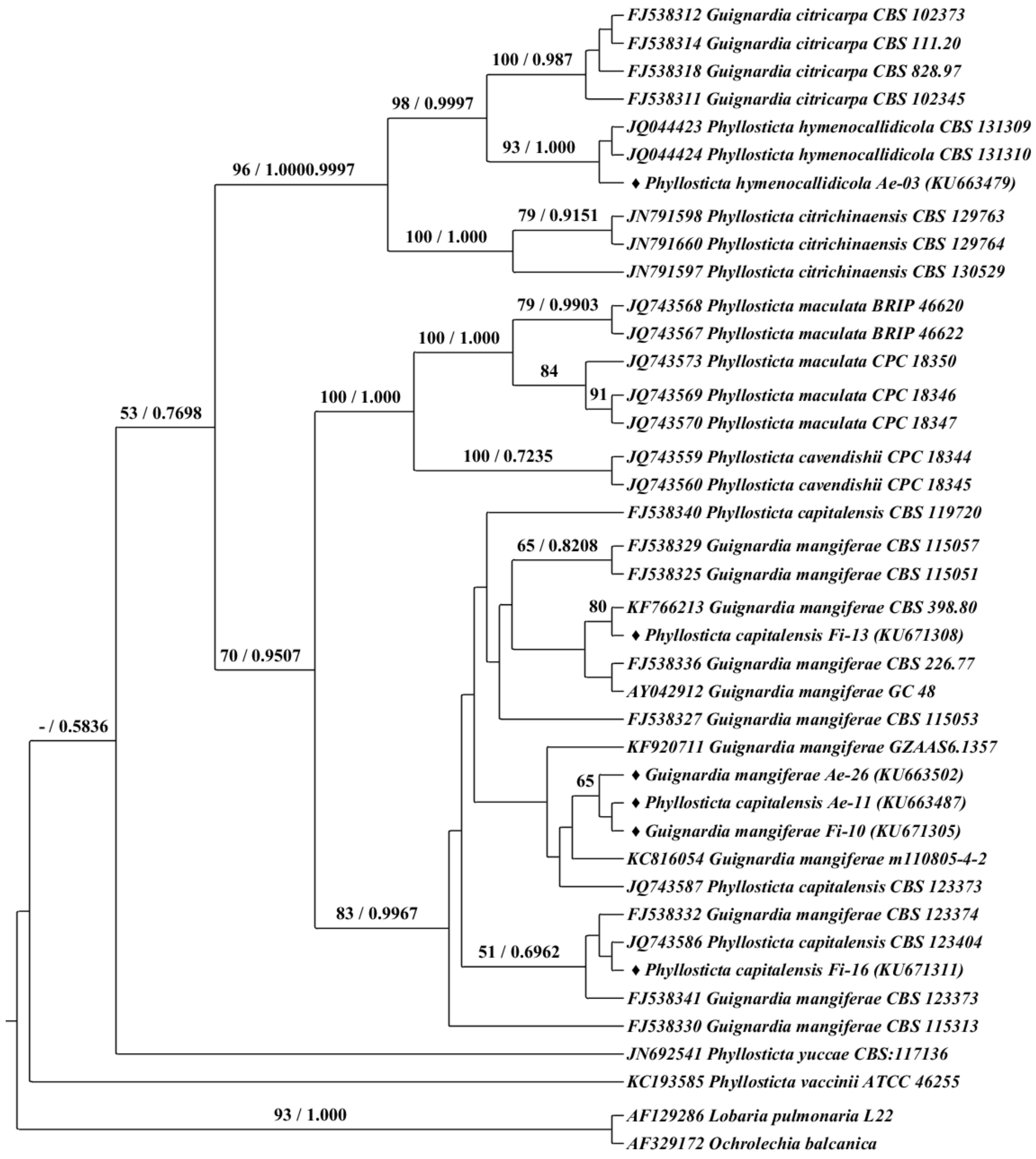

Fig D. ITS sequence based phylogenetic tree of *Guignardia* and *Phyllosticta* query and reference sequences. *Lobaria pulmonaria* L22 and *Ochrolechia balcanica* sequences were used as an outgroup. The query sequences were represented by the symbol "♦". Bootstrap (BP) values ≥50 and posterior probability (PP) values ≥0.5 were represented above the branches of ML tree (BP/PP). The accession number of reference sequences was also given

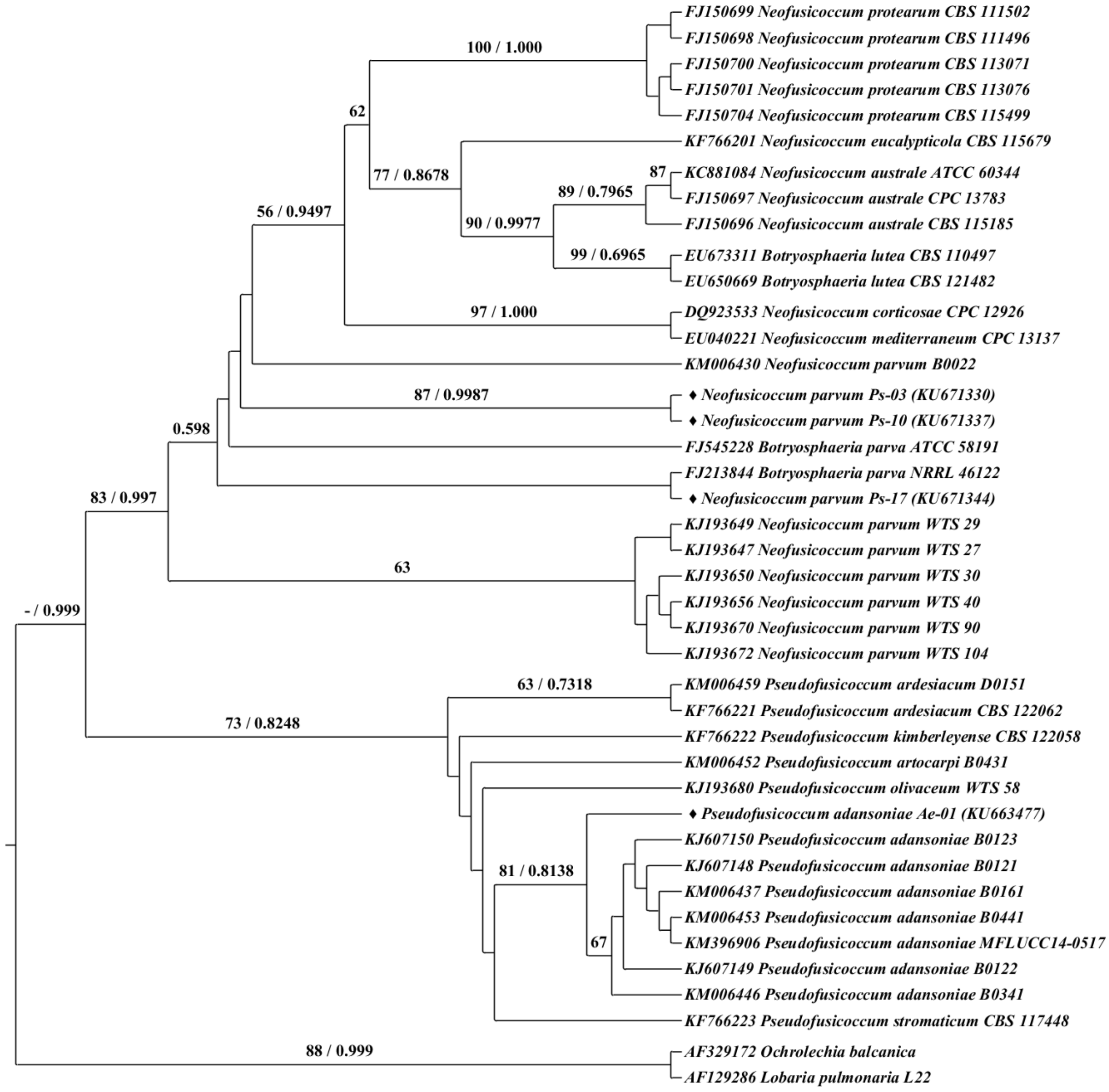

Fig E. ITS sequence based phylogenetic tree of *Neofusicoccum* and *Pseudofusicoccum* query and reference sequences. *Lobaria pulmonaria* L22 and *Ochrolechia balcanica* sequences were used as an outgroup. The query sequences were represented by the symbol "♦". Bootstrap (BP) values ≥50 and posterior probability (PP) values ≥0.5 were represented above the branches of ML tree (BP/PP). The accession number of reference sequences was also given

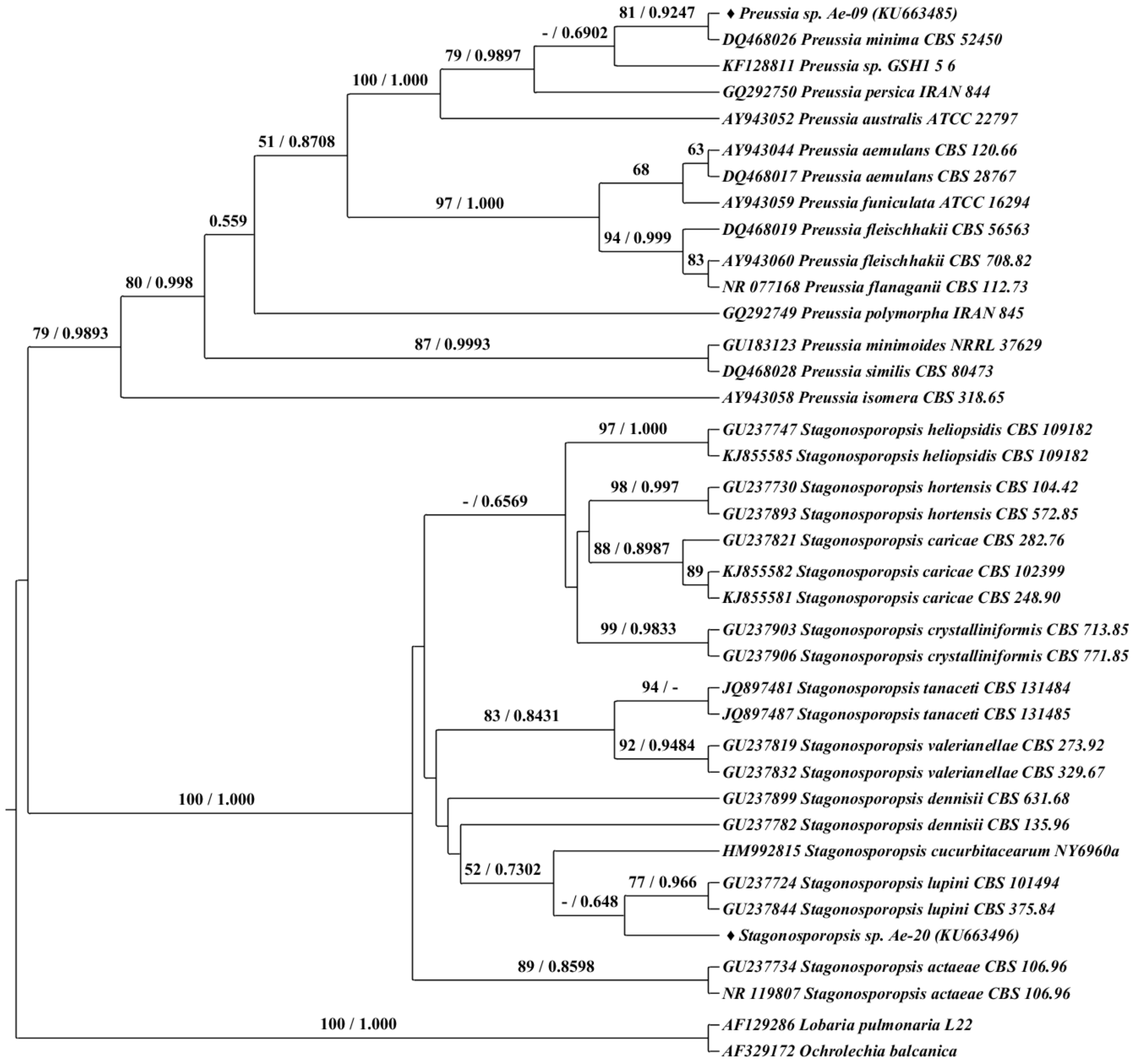

Fig F. ITS sequence based phylogenetic tree of *Preussia* and *Stagonosporopsis* query and reference sequences. *Lobaria pulmonaria* L22 and *Ochrolechia balcanica* sequences were used as an outgroup. The query sequences were represented by the symbol "♦". Bootstrap (BP) values ≥50 and posterior probability (PP) values ≥0.5 were represented above the branches of ML tree (BP/PP). The accession number of reference sequences was also given

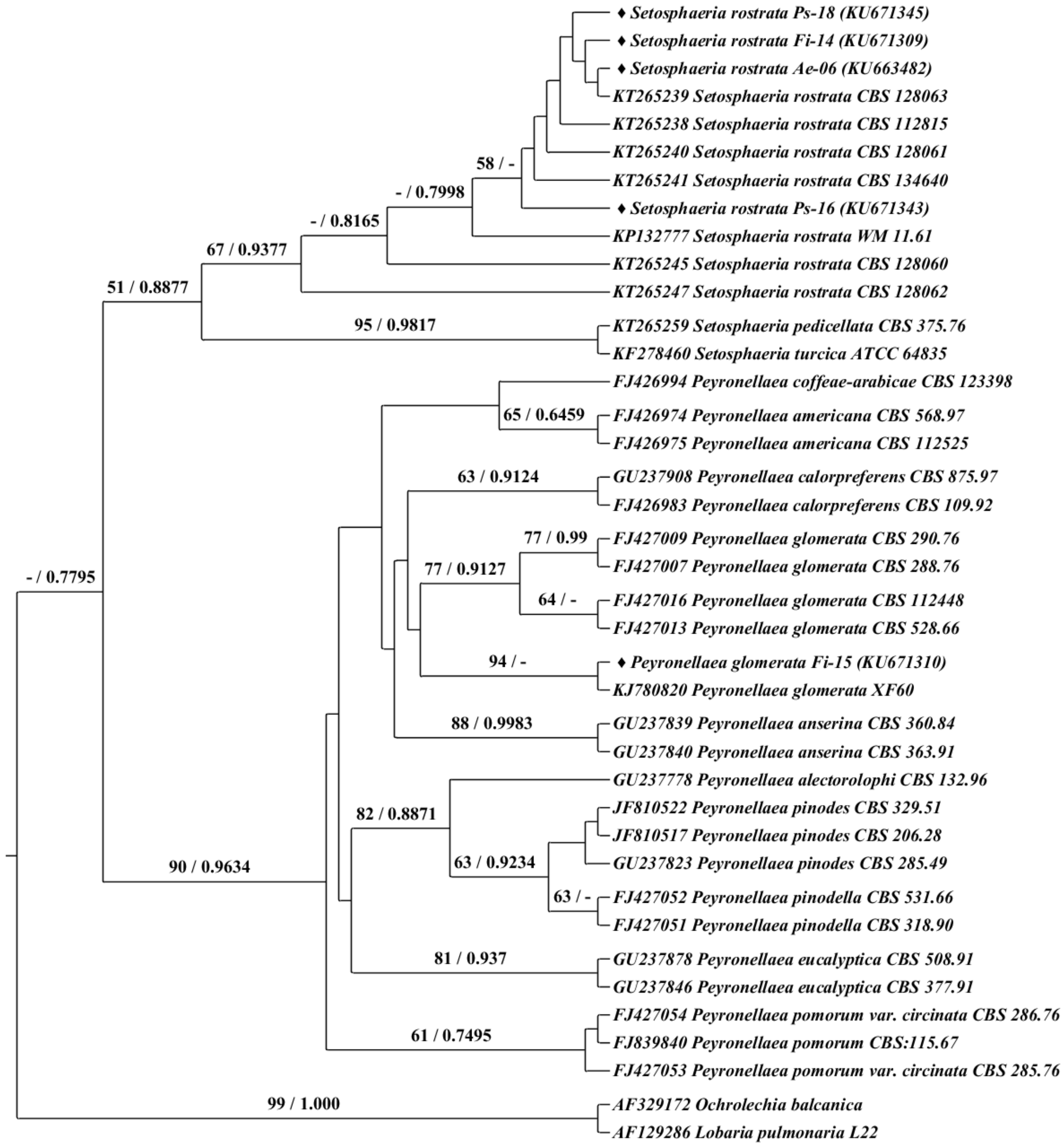

Fig G. ITS sequence based phylogenetic tree of *Setosphaeria* and *Peyronellaea* query and reference sequences. *Lobaria pulmonaria* L22 and *Ochrolechia balcanica* sequences were used as an outgroup. The query sequences were represented by the symbol "♦". Bootstrap (BP) values ≥50 and posterior probability (PP) values ≥0.5 were represented above the branches of ML tree (BP/PP). The accession number of reference sequences was also given

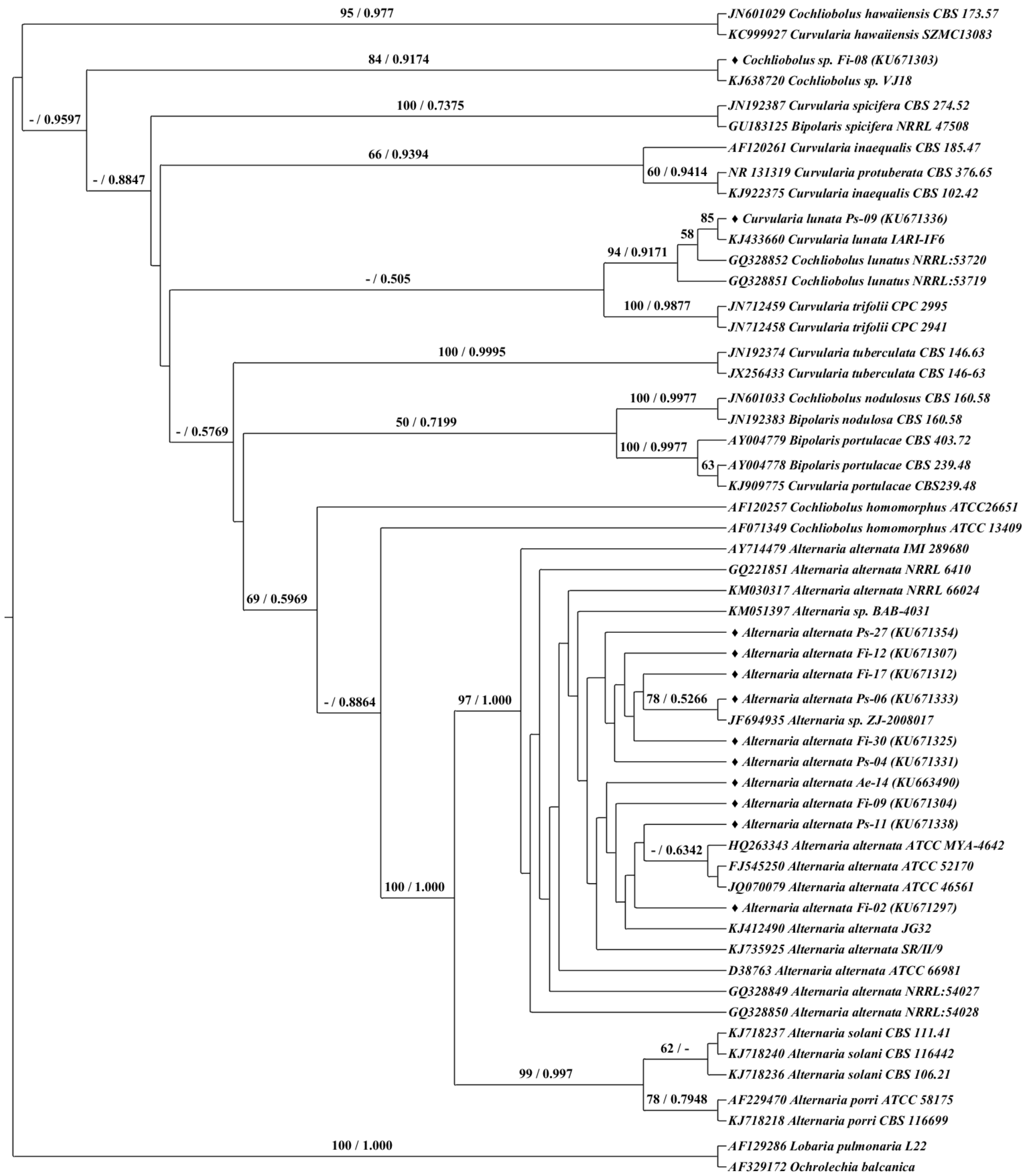

Fig H. ITS sequence based phylogenetic tree of *Curvularia, Cochliobolus* and *Alternaria* query and reference sequences. *Lobaria pulmonaria* L22 and *Ochrolechia balcanica* sequences were used as an outgroup. The query sequences were represented by the symbol "♦". Bootstrap (BP) values ≥50 and posterior probability (PP) values ≥0.5 were represented above the branches of ML tree (BP/PP). The accession number of reference sequences was also given

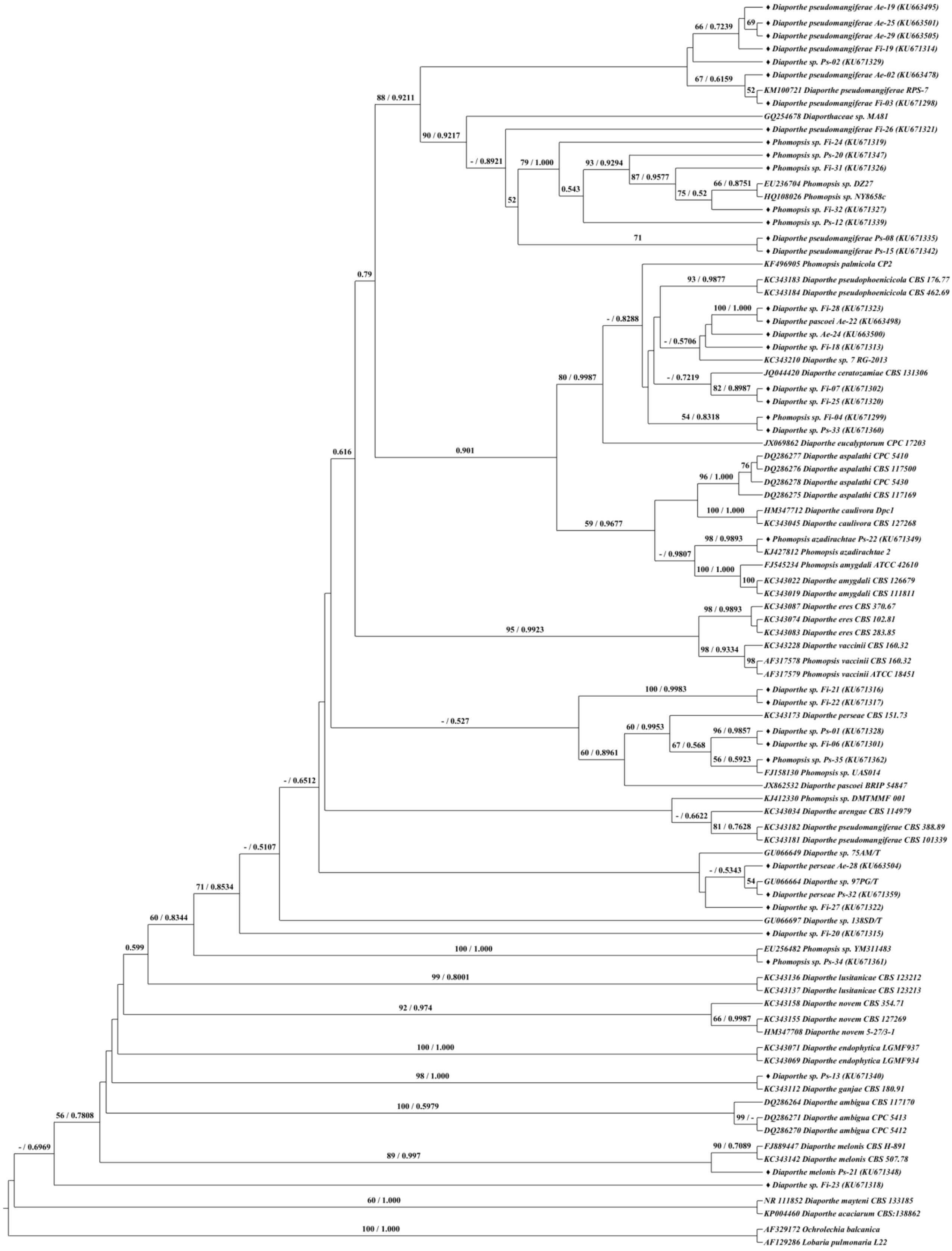

Fig I. ITS sequence based phylogenetic tree of *Diaporthe* and *Phomopsis* query and reference sequences. *Lobaria pulmonaria* L22 and *Ochrolechia balcanica* sequences were used as an outgroup. The query sequences were represented by the symbol "♦". Bootstrap (BP) values ≥50 and posterior probability (PP) values ≥0.5 were represented above the branches of ML tree (BP/PP). The accession number of reference sequences was also given

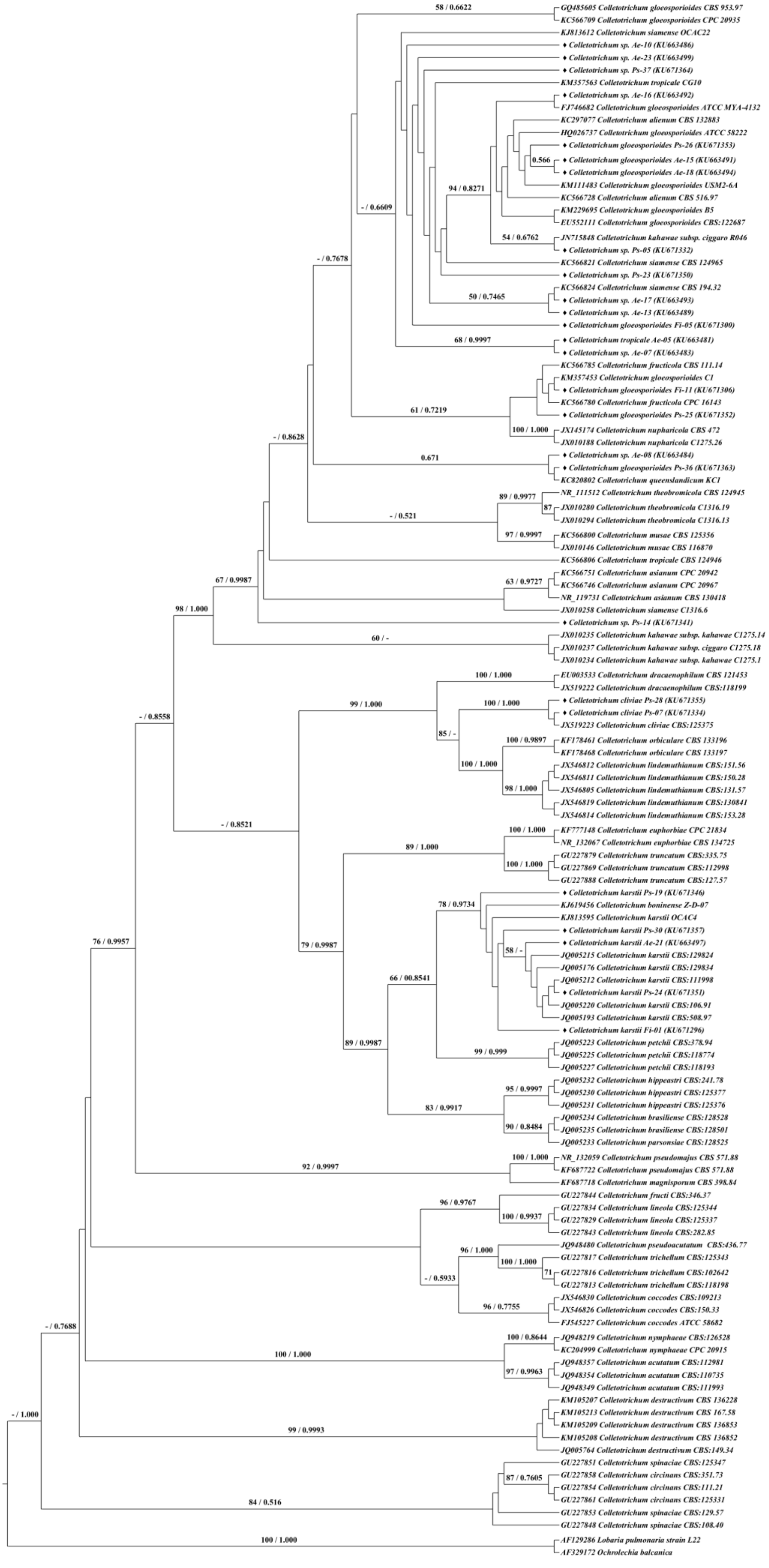

Fig J. ITS sequence based phylogenetic tree of *Colletotrichum* query and reference sequences. *Lobaria pulmonaria* L22 and *Ochrolechia balcanica* sequences were used as an outgroup. The query sequences were represented by the symbol "♦". Bootstrap (BP) values ≥50 and posterior probability (PP) values ≥0.5 were represented above the branches of ML tree (BP/PP). The accession number of reference sequences was also given

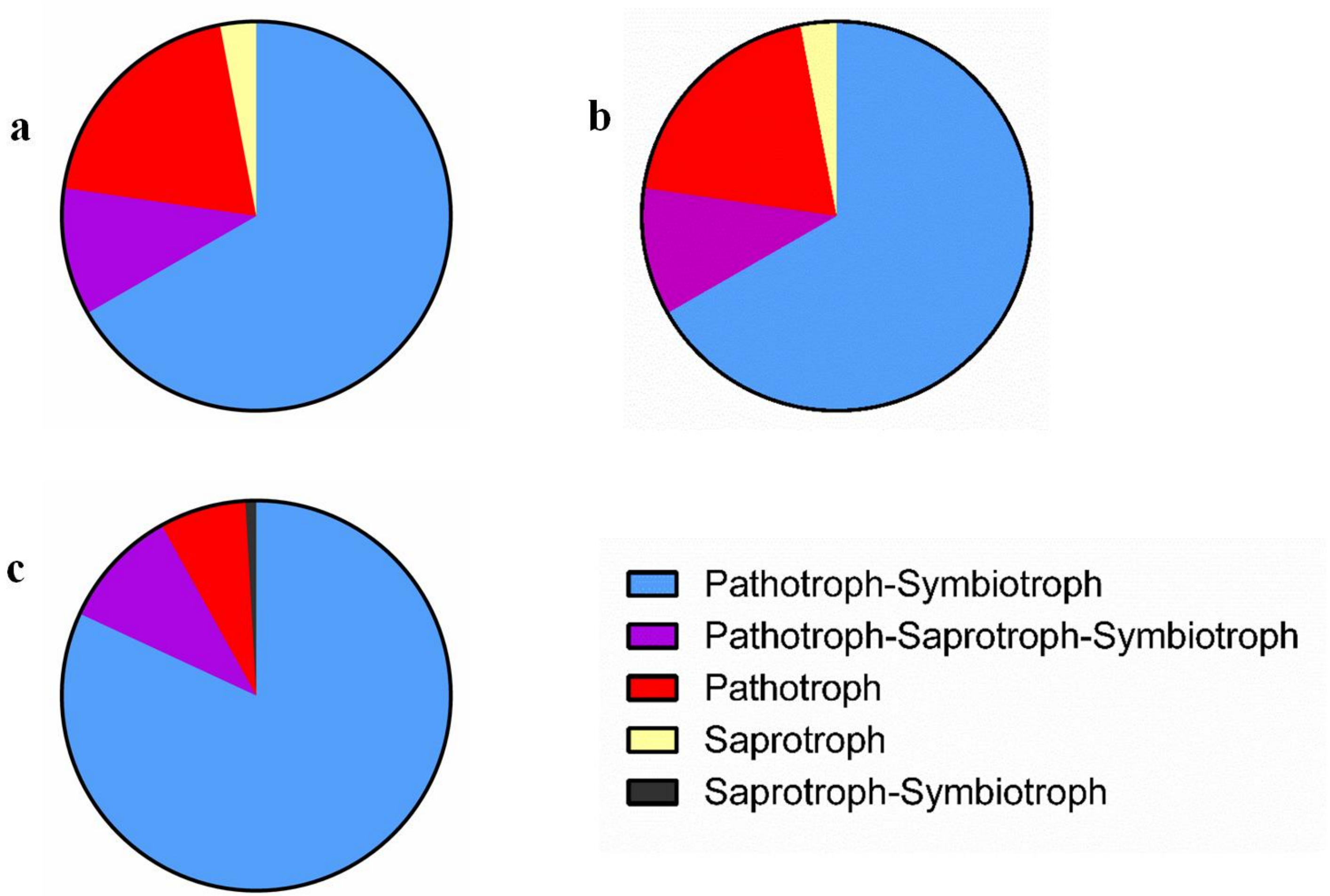

Fig K. Relative abundance of FEF isolates distributed among the trophic guilds as deduced by analysis with FUNGuild: (a) *A. elaeagniodea*, (b) *F. inermis* and (c) *P. serratifolia*.

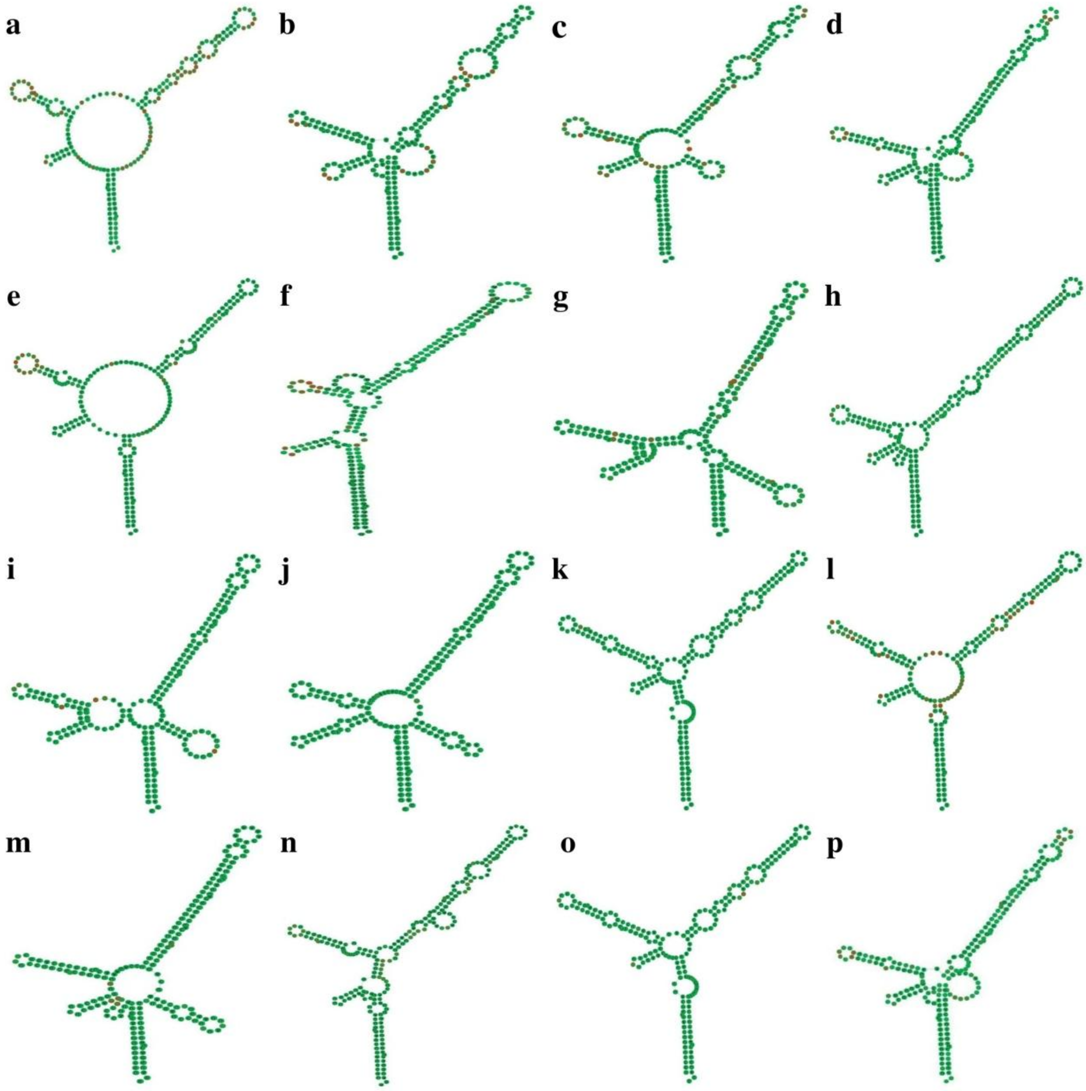

Fig L. Consensus secondary structure of the FEF genera: **(a)** *Alternaria*, **(b)** *Arthroxylaria*, **(c)** *Aureobasidium*, **(d)** *Colletotrichum*, **(e)** *Curvularia* and *Cochliobolus*, **(f)** *Diaporthe* and *Phomopsis*, **(g)** *Guignardia* and *Phyllosticta*, **(h)** *Harknessia*, **(i)** *Neofusicoccum*,**(j)** *Neoscytalidium*, **(k)** *Peyronellaea*, **(l)** *Preussia*, **(m)** *Pseudofusicoccum*, **(N)** *Setosphaeria*, **(O)** *Stagonosporopsis* and **(P)** *Xylaria*.
