# Supplementary material for: Internal transcribed spacer 2 (ITS2) molecular morphometric analysis based species delimitation of foliar endophytic fungi from Aglaia elaeagnoidea, Flacourtia inermis and Premna serratifolia
Source: PLoS One. 2019 Apr 9;14(4):e0215024. doi: 10.1371/journal.pone.0215024 (PMC6456209; doi:10.1371/journal.pone.0215024)
Supplement: S2 File — (PDF) [file pone.0215024.s002.pdf]

# Supplementary File 2

## Table A. Nucleotide base composition and structural free energy of sequences used in the molecular morphometric analysis

| Organism | Total Length | ITS2 region | ITS2 length | A's | C's | G's | U/T's | %GC | Free Energy (kcal/mol) |
|---|---|---|---|---|---|---|---|---|---|
| AF129286 *Lobaria pulmonaria* L22 | 540 | 351-502 | 152 | 29 | 47 | 45 | 31 | 60.53 | -64.66 |
| AF329172 *Ochrolechia balcanica* | 541 | 343-495 | 153 | 24 | 50 | 48 | 31 | 64.47 | -72.09 |

| | | | | | | | | | |
|---|---|---|---|---|---|---|---|---|---|
| ***Guignardia/Phyllosticta*** | | | | | | | | | |
| *Phyllosticta hymenocallidicola* Ae-03 (KU663479) | 568 | 373-538 | 166 | 31 | 47 | 51 | 37 | 59.04 | -84.50 |
| *Phyllosticta capitalensis* Ae-11 (KU663487) | 601 | 402-564 | 163 | 30 | 48 | 47 | 38 | 58.28 | -81.78 |
| *Guignardia mangiferae* Ae-26 (KU663502) | 613 | 407-569 | 163 | 30 | 48 | 47 | 38 | 58.28 | -81.78 |
| *Guignardia mangiferae* Fi-10 (KU671305) | 615 | 404-566 | 163 | 30 | 48 | 47 | 38 | 58.28 | -81.78 |
| *Phyllosticta capitalensis* Fi-13 (KU671308) | 595 | 407-568 | 162 | 30 | 48 | 47 | 37 | 58.64 | -82.58 |
| *Phyllosticta capitalensis* Fi-16 (KU671311) | 601 | 405-567 | 163 | 30 | 48 | 47 | 38 | 58.28 | -75.78 |
| FJ538340 *Phyllosticta capitalensis* CBS 119720 | 618 | 432-594 | 163 | 30 | 48 | 47 | 38 | 58.28 | -81.78 |
| JN791660 *Phyllosticta citrichinaensis* CBS 129764 | 1205 | 984-1147 | 164 | 32 | 45 | 48 | 39 | 56.71 | -81.46 |
| JN791597 *Phyllosticta citrichinaensis* CBS 130529 | 1205 | 984-1147 | 164 | 32 | 46 | 48 | 38 | 57.32 | -77.76 |
| JQ743559 *Phyllosticta cavendishii* CPC 18344 | 634 | 427-595 | 169 | 29 | 59 | 52 | 29 | 65.68 | -90.73 |
| JQ044424 *Phyllosticta hymenocallidicola* CBS 131310 | 656 | 444-609 | 166 | 31 | 47 | 51 | 37 | 59.04 | -84.50 |
| FJ538330 *Guignardia mangiferae* CBS 115313 | 618 | 432-594 | 163 | 30 | 48 | 47 | 38 | 58.28 | -81.78 |
| FJ538325 *Guignardia mangiferae* CBS 115051 | 618 | 432-594 | 163 | 30 | 49 | 47 | 37 | 58.90 | -81.78 |
| JQ743570 *Phyllosticta maculata* CPC 18347 | 634 | 427-595 | 169 | 29 | 62 | 52 | 26 | 67.46 | -89.63 |
| KC193585 *Phyllosticta vaccinii* ATCC 46255 | 664 | 449-614 | 166 | 29 | 53 | 51 | 33 | 62.65 | -88.34 |
| JN692541 *Phyllosticta yuccae* CBS:117136 | 563 | 378-540 | 163 | 30 | 48 | 47 | 38 | 58.28 | -78.43 |
| KC816054 *Guignardia mangiferae* m110805-4-2 | 639 | 419-581 | 163 | 30 | 48 | 47 | 38 | 58.28 | -81.78 |
| JQ044423 *Phyllosticta hymenocallidicola* CBS 131309 | 636 | 423-588 | 166 | 31 | 47 | 51 | 37 | 59.04 | -84.50 |
| KF920711 *Guignardia mangiferae* GZAAS6.1357 | 758 | 558-720 | 163 | 30 | 48 | 47 | 38 | 58.28 | -81.78 |
| | | | **164.31±2.08** | | | | | **59.42±2.77** | **-82.76±3.74** |

| | | | | | | | | | |
|---|---|---|---|---|---|---|---|---|---|
| ***Neofusicoccum*** | | | | | | | | | |
| *Neofusicoccum parvum* Ps-03 (KU671330) | 557 | 365-520 | 156 | 30 | 51 | 41 | 34 | 58.97 | -74.43 |
| *Neofusicoccum parvum* Ps-10 (KU671337) | 535 | 341-496 | 156 | 30 | 51 | 41 | 34 | 58.97 | -74.43 |
| *Neofusicoccum parvum* Ps-17 (KU671344) | 547 | 350-505 | 156 | 30 | 51 | 41 | 34 | 58.97 | -74.43 |
| FJ150696 *Neofusicoccum australe* CBS 115185 | 704 | 477-631 | 155 | 27 | 50 | 43 | 35 | 60.00 | -76.97 |
| DQ923533 *Neofusicoccum corticosae* CPC 12926 | 1753 | 375-529 | 155 | 29 | 52 | 41 | 33 | 60.00 | -79.47 |
| KF766201 *Neofusicoccum eucalypticola* CBS 115679 | 586 | 374-528 | 155 | 27 | 47 | 42 | 39 | 57.42 | -77.37 |
| EU650669 *Botryosphaeria lutea* CBS 121482 | 579 | 367-521 | 155 | 27 | 49 | 43 | 36 | 59.35 | -77.57 |
| EU040221 *Neofusicoccum mediterraneum* CPC 13137 | 1551 | 477-631 | 155 | 27 | 51 | 43 | 34 | 60.65 | -77.37 |
| KJ193672 *Neofusicoccum parvum* WTS 104 | 572 | 368-523 | 156 | 30 | 51 | 41 | 34 | 58.97 | -74.43 |
| FJ545228 *Botryosphaeria parva* ATCC 58191 | 554 | 353-508 | 156 | 30 | 51 | 41 | 34 | 58.97 | -74.43 |
| FJ213844 *Botryosphaeria parva* NRRL 46122 | 999 | 294-449 | 156 | 30 | 50 | 41 | 35 | 58.33 | -74.43 |
| FJ150704 *Neofusicoccum protearum* CBS 115499 | 704 | 477-631 | 155 | 27 | 51 | 43 | 34 | 60.65 | -79.26 |
| | | | **155.50±0.52** | | | | | **59.27±0.93** | **-76.22±2.00** |
| ***Pseudofusicoccum*** | | | | | | | | | |
| *Pseudofusicoccum adansoniae* Ae-01 (KU663477) | 488 | 298-454 | 157 | 29 | 49 | 44 | 35 | 59.24 | -89.59 |
| KF766221 *Pseudofusicoccum ardesiacum* CBS 122062 | 592 | 378-534 | 157 | 30 | 50 | 43 | 34 | 59.24 | -85.98 |
| KM006452 *Pseudofusicoccum artocarpi* B0431 | 530 | 370-530 | 157 | 30 | 49 | 43 | 35 | 58.60 | -70.25 |
| KM006453 *Pseudofusicoccum adansoniae* B0441 | 538 | 378-538 | 157 | 29 | 49 | 44 | 35 | 59.24 | -73.48 |
| KF766222 *Pseudofusicoccum kimberleyense* CBS 122058 | 592 | 378-534 | 157 | 30 | 49 | 43 | 35 | 58.60 | -85.98 |
| KJ193680 *Pseudofusicoccum olivaceum* WTS 58 | 574 | 371-525 | 155 | 30 | 47 | 42 | 36 | 57.42 | -84.22 |
| KF766223 *Pseudofusicoccum stromaticum* CBS 117448 | 592 | 378-534 | 157 | 28 | 50 | 45 | 34 | 60.51 | -89.59 |
| KM396906 *Pseudofusicoccum adansoniae* MFLUCC14-0517 | 613 | 352-508 | 157 | 29 | 49 | 44 | 35 | 59.24 | -89.59 |
| | | | **156.75±0.70** | | | | | **59.01±0.87** | **-83.59±7.55** |
| ***Neoscytalidium*** | | | | | | | | | |
| *Neoscytalidium dimidiatum* Ae-27 (KU663503) | 484 | 290-445 | 156 | 26 | 54 | 45 | 31 | 63.46 | -84.32 |
| KF531819 *Neoscytalidium dimidiatum* CBS 251.49 | 579 | 366-521 | 156 | 26 | 54 | 45 | 31 | 63.46 | -86.82 |
| KF531816 *Scytalidium hyalinum* CBS 145.78 | 579 | 366-521 | 156 | 26 | 54 | 45 | 31 | 63.46 | -86.82 |
| KF766207 *Neoscytalidium novaehollandiae* CBS 122071 | 588 | 375-530 | 156 | 24 | 56 | 46 | 30 | 65.38 | -88.12 |

| | | | | | | | | | |
|---|---|---|---|---|---|---|---|---|---|
| KP132489 *Neoscytalidium dimidiatum* PWQ2367 | 570 | 365-520 | 156 | 26 | 54 | 45 | 31 | 63.46 | -86.82 |
| | | | **156.00±0.00** | | | | | **63.84±0.85** | **-86.58±1.38** |
| ***Aureobasidium*** | | | | | | | | | |
| *Aureobasidium thailandense* Ae-12 (KU663488) | 552 | 359-524 | 166 | 35 | 47 | 43 | 41 | 54.22 | -72.26 |
| EU167576 *Aureobasidium caulivorum* CBS 242.64 | 3465 | 2417-2573 | 157 | 33 | 46 | 41 | 37 | 55.41 | -74.79 |
| JN712489 *Aureobasidium leucospermi* CPC 15180 | 676 | 501-656 | 156 | 34 | 37 | 41 | 44 | 50.00 | -69.59 |
| JX462671 *Aureobasidium pullulans* NRRL Y-7703 | 1111 | 348-503 | 156 | 34 | 38 | 41 | 43 | 50.64 | -71.39 |
| JX462675 *Aureobasidium thailandense* NRRL 58543 | 1638 | 839-1004 | 166 | 35 | 46 | 44 | 41 | 54.22 | -81.19 |
| | | | **160.20±5.31** | | | | | **52.90±2.41** | **-73.84±4.51** |
| ***Harknessia*** | | | | | | | | | |
| *Harknessia* sp. Ae-04 (KU663480) | 454 | 244-412 | 169 | 43 | 41 | 38 | 47 | 46.75 | -74.85 |
| AY720719 *Harknessia capensis* CBS 111829 | 628 | 421-582 | 162 | 35 | 47 | 40 | 40 | 53.70 | -68.55 |
| AY720746 *Harknessia eucalyptorum* CBS 113620 | 621 | 414-575 | 162 | 35 | 46 | 41 | 40 | 53.70 | -68.55 |
| JQ706088 *Harknessia ellipsoidea* CPC 17113 | 715 | 525-686 | 162 | 36 | 46 | 40 | 40 | 53.08 | -68.55 |
| EF110615 *Harknessia gibbosa* CBS 120033 | 1794 | 435-596 | 162 | 35 | 47 | 40 | 40 | 53.70 | -68.55 |
| AY720722 *Harknessia globispora* CBS 111578 | 603 | 421-582 | 162 | 35 | 47 | 40 | 40 | 53.70 | -68.55 |
| AY720726 *Harknessia hawaiiensis* CBS 111122 | 628 | 421-582 | 162 | 36 | 46 | 40 | 40 | 53.08 | -64.70 |
| AY720731 *Harknessia leucospermi* CBS 112619 | 628 | 421-582 | 162 | 34 | 48 | 41 | 39 | 54.94 | -67.02 |
| AY720736 *Harknessia protearum* CBS 111831 | 626 | 418-580 | 162 | 34 | 47 | 41 | 40 | 54.32 | -68.55 |
| DQ923532 *Harknessia rhabdosphaera* CBS 120082 | 1800 | 424-585 | 162 | 36 | 45 | 40 | 41 | 52.47 | -65.09 |
| JQ706118 *Harknessia rhabdosphaera* CPC 12847 | 656 | 449-610 | 162 | 38 | 42 | 40 | 42 | 50.62 | -65.74 |
| JQ706113 *Harknessia ravenstreetina* CPC 17209 | 720 | 530-691 | 162 | 36 | 46 | 40 | 40 | 53.09 | -68.55 |
| JQ706119 *Harknessia* sp. CPC 11153 | 617 | 424-585 | 162 | 35 | 46 | 41 | 40 | 53.70 | -64.70 |
| JQ922185 *Harknessia* sp. CCG-2012 | 1113 | 372-532 | 161 | 35 | 42 | 39 | 45 | 50.31 | -73.80 |
| | | | **162.43±1.91** | | | | | **52.65±2.12** | **-68.27±3.01** |
| ***Diaporthe/Phomopsis*** | | | | | | | | | |
| *Diaporthe pseudomangiferae* Ae-02 (KU663478) | 556 | 352-510 | 159 | 34 | 49 | 43 | 33 | 57.86 | -78.65 |
| *Diaporthe pseudomangiferae* Ae-19 (KU663495) | 546 | 348-505 | 158 | 34 | 49 | 43 | 32 | 58.23 | -78.82 |
| *Diaporthe pascoei* Ae-22 (KU663498) | 544 | 347-504 | 158 | 35 | 49 | 40 | 34 | 56.33 | -77.32 |
| *Diaporthe* sp. Ae-24 (KU663500) | 553 | 347-504 | 158 | 35 | 48 | 40 | 35 | 55.70 | -75.22 |

| | | | | | | | | | |
|---|---|---|---|---|---|---|---|---|---|
| *Diaporthe pseudomangiferae* Ae-25 (KU663501) | 578 | 362-519 | 158 | 34 | 49 | 43 | 32 | 58.23 | -78.82 |
| *Diaporthe perseae* Ae-28 (KU663504) | 539 | 342-499 | 158 | 35 | 49 | 41 | 33 | 56.96 | -77.50 |
| *Diaporthe pseudomangiferae* Ae-29 (KU663505) | 574 | 362-519 | 158 | 34 | 49 | 43 | 32 | 58.23 | -78.82 |
| *Diaporthe pseudomangiferae* Fi-03 (KU671298) | 549 | 346-504 | 159 | 34 | 49 | 43 | 33 | 57.86 | -78.65 |
| *Phomopsis* sp. Fi-04 (KU671299) | 545 | 342-502 | 161 | 37 | 50 | 40 | 34 | 55.90 | -76.92 |
| *Diaporthe* sp. Fi-06 (KU671301) | 547 | 348-505 | 158 | 35 | 50 | 40 | 33 | 56.96 | -74.79 |
| *Diaporthe* sp. Fi-07 (KU671302) | 551 | 344-501 | 158 | 35 | 50 | 40 | 33 | 56.96 | -74.79 |
| *Diaporthe* sp. Fi-18 (KU671313) | 547 | 345-502 | 158 | 36 | 48 | 40 | 34 | 55.70 | -75.12 |
| *Diaporthe pseudomangiferae* Fi-19 (KU671314) | 550 | 347-504 | 158 | 34 | 49 | 43 | 32 | 58.23 | -78.82 |
| *Diaporthe* sp. Fi-20 (KU671315) | 546 | 347-504 | 158 | 35 | 47 | 43 | 33 | 56.96 | -77.90 |
| *Diaporthe* sp. Fi-21 (KU671316) | 550 | 348-505 | 158 | 34 | 48 | 41 | 35 | 56.33 | -75.22 |
| *Diaporthe* sp. Fi-22 (KU671317) | 550 | 349-506 | 158 | 34 | 48 | 41 | 35 | 56.33 | -75.22 |
| *Diaporthe* sp. Fi-23 (KU671318) | 544 | 347-503 | 157 | 34 | 46 | 45 | 32 | 57.96 | -81.15 |
| *Phomopsis* sp. Fi-24 (KU671319) | 551 | 349-511 | 163 | 36 | 51 | 41 | 35 | 56.44 | -75.02 |
| *Diaporthe* sp. Fi-25 (KU671320) | 554 | 346-503 | 158 | 35 | 50 | 40 | 33 | 56.96 | -74.79 |
| *Diaporthe pseudomangiferae* Fi-26 (KU671321) | 553 | 351-509 | 159 | 34 | 50 | 43 | 32 | 58.49 | -81.25 |
| *Diaporthe* sp. Fi-27 (KU671322) | 549 | 346-504 | 159 | 35 | 48 | 42 | 34 | 56.60 | -79.57 |
| *Diaporthe* sp. Fi-28 (KU671323) | 557 | 358-516 | 159 | 35 | 49 | 40 | 35 | 55.97 | -80.93 |
| *Phomopsis* sp. Fi-31 (KU671326) | 549 | 345-507 | 163 | 32 | 52 | 45 | 34 | 59.51 | -85.32 |
| *Phomopsis* sp. Fi-32 (KU671327) | 487 | 285-448 | 164 | 32 | 52 | 44 | 36 | 58.54 | -87.69 |
| *Diaporthe* sp. Ps-01 (KU671328) | 546 | 353-508 | 156 | 35 | 50 | 39 | 32 | 57.05 | -73.15 |
| *Diaporthe* sp. Ps-02 (KU671329) | 548 | 351-509 | 159 | 34 | 49 | 43 | 33 | 57.86 | -79.82 |
| *Diaporthe pseudomangiferae* Ps-08 (KU671335) | 571 | 363-521 | 159 | 34 | 49 | 43 | 33 | 57.86 | -78.65 |
| *Phomopsis* sp. Ps-12 (KU671339) | 555 | 350-511 | 162 | 35 | 52 | 41 | 34 | 57.41 | -81.12 |
| *Diaporthe* sp. Ps-13 (KU671340) | 544 | 342-500 | 159 | 34 | 48 | 45 | 32 | 58.49 | -87.15 |
| *Diaporthe pseudomangiferae* Ps-15 (KU671342) | 551 | 349-507 | 159 | 34 | 49 | 43 | 33 | 57.86 | -78.65 |
| *Phomopsis* sp. Ps-20 (KU671347) | 550 | 347-509 | 163 | 32 | 52 | 44 | 35 | 58.90 | -82.65 |
| *Diaporthe melonis* Ps-21 (KU671348) | 545 | 347-505 | 159 | 31 | 51 | 45 | 32 | 60.38 | -82.85 |
| *Phomopsis azadirachtae* Ps-22 (KU671349) | 548 | 351-510 | 160 | 36 | 50 | 41 | 33 | 56.88 | -73.89 |
| *Diaporthe perseae* Ps-32 (KU671359) | 576 | 360-517 | 158 | 35 | 49 | 41 | 33 | 56.96 | -77.50 |
| *Diaporthe* sp. Ps-33 (KU671360) | 542 | 344-500 | 157 | 35 | 48 | 40 | 34 | 56.05 | -76.02 |

| | | | | | | | | | |
|---|---|---|---|---|---|---|---|---|---|
| *Phomopsis* sp. Ps-34 (KU671361) | 543 | 342-498 | 157 | 32 | 48 | 44 | 33 | 58.60 | -87.60 |
| *Phomopsis* sp. Ps-35 (KU671362) | 555 | 348-505 | 158 | 35 | 48 | 40 | 35 | 55.70 | -75.22 |
| KC343034 *Diaporthe arengae* CBS 114979 | 571 | 371-528 | 158 | 35 | 50 | 41 | 32 | 57.59 | -81.90 |
| KC343022 *Diaporthe amygdali* CBS 126679 | 583 | 376-540 | 165 | 38 | 47 | 46 | 34 | 56.36 | -81.19 |
| DQ286264 *Diaporthe ambigua* CBS 117170 | 524 | 342-500 | 159 | 33 | 49 | 46 | 31 | 59.75 | -87.45 |
| DQ286276 *Diaporthe aspalathi* CBS 117500 | 522 | 342-498 | 157 | 31 | 50 | 47 | 29 | 61.78 | -89.80 |
| KP004460 *Diaporthe acaciarum* CBS 138862 | 555 | 362-520 | 159 | 32 | 49 | 44 | 34 | 58.49 | -82.55 |
| KC343045 *Diaporthe caulivora* CBS 127268 | 571 | 371-528 | 158 | 34 | 48 | 43 | 33 | 57.59 | -76.66 |
| JQ044420 *Diaporthe ceratozamiae* CBS 131306 | 682 | 476-633 | 158 | 35 | 48 | 41 | 34 | 56.33 | -76.39 |
| KC343087 *Diaporthe eres* CBS 370.67 | 578 | 374-535 | 162 | 35 | 53 | 43 | 31 | 59.26 | -80.05 |
| KC343071 *Diaporthe endophytica* LGMF937 | 569 | 370-526 | 157 | 34 | 47 | 44 | 32 | 57.96 | -81.45 |
| JX069862 *Diaporthe eucalyptorum* CPC 17203 | 674 | 471-628 | 158 | 35 | 49 | 43 | 31 | 58.23 | -80.68 |
| KC343112 *Diaporthe ganjae* CBS 180.91 | 568 | 367-525 | 159 | 34 | 50 | 45 | 30 | 59.75 | -85.45 |
| KC343137 *Diaporthe lusitanicae* CBS 123213 | 570 | 371-527 | 157 | 34 | 48 | 44 | 31 | 58.60 | -79.75 |
| FJ889447 *Diaporthe melonis* CBS H-891 | 540 | 344-502 | 159 | 31 | 51 | 45 | 32 | 60.38 | -82.85 |
| NR_111852 *Diaporthe mayteni* CBS 133185 | 565 | 369-522 | 154 | 27 | 52 | 44 | 31 | 62.34 | -81.67 |
| KC343155 *Diaporthe novem* CBS 127269 | 569 | 370-526 | 157 | 34 | 47 | 43 | 33 | 57.32 | -78.15 |
| KC343158 *Diaporthe novem* CBS 354.71 | 569 | 370-526 | 157 | 34 | 47 | 44 | 32 | 57.96 | -81.45 |
| KC343184 *Diaporthe pseudophoenicicola* CBS 462.69 | 571 | 371-528 | 158 | 34 | 49 | 40 | 35 | 56.33 | -75.26 |
| KC343182 *Diaporthe pseudomangiferae* CBS 388.89 | 571 | 371-528 | 158 | 35 | 48 | 41 | 34 | 56.33 | -83.32 |
| KC343173 *Diaporthe perseae* CBS 151.73 | 571 | 371-528 | 158 | 35 | 49 | 41 | 33 | 56.96 | -77.50 |
| KC343228 *Diaporthe vaccinii* CBS 160.32 | 576 | 374-533 | 160 | 35 | 52 | 41 | 32 | 58.13 | -75.65 |
| KC343210 *Diaporthe* sp. 7 RG-2013 CBS 458.78 | 570 | 370-527 | 158 | 35 | 48 | 40 | 35 | 55.70 | -75.22 |
| EU256482 *Phomopsis* sp. YM311483 | 538 | 322-478 | 157 | 32 | 48 | 44 | 33 | 58.60 | -81.60 |
| FJ158130 *Phomopsis* sp. UAS014 | 536 | 343-502 | 160 | 36 | 50 | 40 | 34 | 56.25 | -79.12 |
| GQ254678 *Diaporthaceae* sp. MA81 | 586 | 379-537 | 159 | 33 | 49 | 44 | 33 | 58.49 | -78.15 |
| GU066649 *Diaporthe* sp. 75AM/T | 567 | 351-508 | 158 | 36 | 48 | 40 | 34 | 55.70 | -78.22 |
| GU066664 *Diaporthe* sp. 97PG/T | 576 | 360-517 | 158 | 35 | 49 | 41 | 33 | 56.96 | -77.50 |
| GU066697 *Diaporthe* sp. 138SD/T | 567 | 351-508 | 158 | 35 | 46 | 43 | 34 | 56.33 | -78.82 |
| HQ108026 *Phomopsis* sp. NY8658c | 550 | 343-504 | 162 | 32 | 52 | 44 | 34 | 59.26 | -82.82 |
| KF496905 *Phomopsis palmicola* CP2 | 541 | 342-503 | 162 | 37 | 49 | 40 | 36 | 54.94 | -77.92 |

| | | | | | | | | | |
|---|---|---|---|---|---|---|---|---|---|
| KJ427812 *Phomopsis azadirachtae* 2 | 541 | 344-503 | 160 | 36 | 50 | 41 | 33 | 56.88 | -73.89 |
| KM100721 *Diaporthe pseudomangiferae* RPS-7 | 554 | 351-509 | 159 | 34 | 49 | 43 | 33 | 57.86 | -78.65 |
| JX862532 *Diaporthe pascoei* BRIP 54847 | 703 | 508-665 | 158 | 34 | 49 | 40 | 35 | 56.33 | -77.32 |
| KJ412330 *Phomopsis* sp. DMTMMF 001 | 1656 | 443-600 | 158 | 35 | 48 | 41 | 34 | 56.33 | -76.32 |
| EU236704 *Phomopsis* sp. DZ27 | 595 | 372-533 | 162 | 32 | 52 | 44 | 34 | 59.26 | -82.82 |
| | | | **158.85±1.92** | | | | | **57.60±1.45** | **-79.30±3.71** |
| ***Colletotrichum*** | | | | | | | | | |
| *Colletotrichum tropicale* Ae-05 (KU663481) | 534 | 341-497 | 157 | 31 | 47 | 41 | 38 | 56.05 | -74.09 |
| *Colletotrichum* sp. Ae-07 (KU663483) | 474 | 296-450 | 155 | 30 | 47 | 40 | 38 | 56.13 | -72.79 |
| *Colletotrichum* sp. Ae-08 (KU663484) | 554 | 345-502 | 158 | 30 | 50 | 42 | 36 | 58.23 | -73.13 |
| *Colletotrichum* sp. Ae-10 (KU663486) | 549 | 344-500 | 157 | 31 | 47 | 41 | 38 | 56.05 | -74.09 |
| *Colletotrichum* sp. Ae-13 (KU663489) | 538 | 343-499 | 157 | 31 | 47 | 41 | 38 | 56.05 | -74.09 |
| *Colletotrichum gloeosporioides* Ae-15 (KU663491) | 534 | 344-501 | 158 | 31 | 49 | 41 | 37 | 56.96 | -74.03 |
| *Colletotrichum* sp. Ae-16 (KU663492) | 541 | 350-507 | 158 | 31 | 50 | 41 | 36 | 57.59 | -66.53 |
| *Colletotrichum* sp. Ae-17 (KU663493) | 539 | 344-500 | 157 | 31 | 47 | 41 | 38 | 56.05 | -74.09 |
| *Colletotrichum gloeosporioides* Ae-18 (KU663494) | 529 | 343-499 | 157 | 31 | 49 | 41 | 36 | 57.32 | -74.09 |
| *Colletotrichum karstii* Ae-21 (KU663497) | 557 | 361-518 | 158 | 30 | 50 | 42 | 36 | 58.23 | -74.55 |
| *Colletotrichum* sp. Ae-23 (KU663499) | 542 | 345-501 | 157 | 31 | 47 | 41 | 38 | 56.05 | -74.09 |
| *Colletotrichum karstii* Fi-01 (KU671296) | 563 | 364-521 | 158 | 30 | 49 | 42 | 37 | 57.59 | -73.43 |
| *Colletotrichum gloeosporioides* Fi-05 (KU671300) | 543 | 344-500 | 157 | 31 | 47 | 41 | 38 | 56.05 | -74.09 |
| *Colletotrichum gloeosporioides* Fi-11 (KU671306) | 543 | 344-501 | 158 | 31 | 49 | 41 | 37 | 56.96 | -74.03 |
| *Colletotrichum* sp. Ps-05 (KU671332) | 533 | 355-508 | 155 | 31 | 48 | 41 | 35 | 57.42 | -76.62 |
| *Colletotrichum cliviae* Ps-07 (KU671334) | 568 | 361-518 | 158 | 33 | 47 | 39 | 29 | 54.43 | -80.29 |
| *Colletotrichum* sp. Ps-14 (KU671341) | 538 | 341-497 | 157 | 31 | 48 | 41 | 37 | 56.69 | -74.09 |
| *Colletotrichum karstii* Ps-19 (KU671346) | 572 | 377-533 | 158 | 29 | 50 | 42 | 37 | 58.23 | -72.53 |
| *Colletotrichum* sp. Ps-23 (KU671350) | 539 | 344-500 | 157 | 31 | 47 | 41 | 38 | 56.05 | -74.09 |
| *Colletotrichum karstii* Ps-24 (KU671351) | 559 | 362-519 | 158 | 30 | 49 | 42 | 37 | 57.59 | -73.43 |
| *Colletotrichum gloeosporioides* Ps-25 (KU671352) | 540 | 344-501 | 158 | 31 | 49 | 41 | 37 | 56.96 | -74.03 |
| *Colletotrichum gloeosporioides* Ps-26 (KU671353) | 541 | 347-503 | 157 | 31 | 48 | 41 | 37 | 56.69 | -74.09 |
| *Colletotrichum cliviae* Ps-28 (KU671355) | 541 | 344-501 | 158 | 33 | 57 | 39 | 29 | 60.76 | -80.29 |
| *Colletotrichum karstii* Ps-30 (KU671357) | 563 | 361-518 | 158 | 30 | 49 | 42 | 37 | 57.59 | -73.43 |

| | | | | | | | | | |
|---|---|---|---|---|---|---|---|---|---|
| *Colletotrichum gloeosporioides* Ps-36 (KU671363) | 544 | 345-502 | 158 | 31 | 50 | 41 | 36 | 57.59 | -74.03 |
| *Colletotrichum* sp. Ps-37 (KU671364) | 539 | 341-497 | 157 | 31 | 47 | 41 | 38 | 56.05 | -74.09 |
| NR_119731 *Colletotrichum asianum* CBS 130418 | 586 | 381-537 | 157 | 31 | 49 | 41 | 36 | 57.32 | -74.09 |
| JQ948349 *Colletotrichum acutatum* CBS:111993 | 539 | 350-506 | 157 | 32 | 50 | 42 | 33 | 58.60 | -74.50 |
| KC566728 *Colletotrichum alienum* CBS 516.97 | 536 | 341-498 | 158 | 31 | 49 | 41 | 37 | 56.96 | -74.03 |
| JQ005235 *Colletotrichum brasiliense* CBS:128501 | 554 | 360-516 | 157 | 29 | 48 | 42 | 38 | 57.32 | -74.89 |
| FJ545227 *Colletotrichum coccodes* ATCC 58682 | 551 | 349-504 | 156 | 32 | 43 | 42 | 39 | 54.49 | -73.23 |
| GU227861 *Colletotrichum circinans* CBS:125331 | 515 | 342-496 | 155 | 31 | 47 | 40 | 37 | 56.13 | -70.78 |
| JX519223 *Colletotrichum cliviae* CBS:125375 | 539 | 344-501 | 158 | 33 | 57 | 39 | 29 | 60.76 | -80.29 |
| JQ005764 *Colletotrichum destructivum* CBS:149.34 | 549 | 354-511 | 158 | 33 | 49 | 42 | 34 | 57.59 | -74.87 |
| KF777148 *Colletotrichum euphorbiae* CPC 21834 | 567 | 371-529 | 159 | 30 | 52 | 41 | 36 | 58.49 | -79.33 |
| KC566780 *Colletotrichum fructicola* CPC 16143 | 536 | 341-498 | 158 | 31 | 49 | 41 | 37 | 56.96 | -74.03 |
| GU227844 *Colletotrichum fructi* CBS:346.37 | 512 | 339-493 | 155 | 30 | 42 | 43 | 40 | 54.84 | -73.28 |
| EU552111 *Colletotrichum gloeosporioides* CBS:122687 | 1539 | 473-630 | 158 | 31 | 49 | 41 | 37 | 56.96 | -74.03 |
| KC566709 *Colletotrichum gloeosporioides* CPC 20935 | 536 | 341-498 | 158 | 31 | 50 | 41 | 36 | 57.59 | -74.03 |
| JQ005232 *Colletotrichum hippeastri* CBS:241.78 | 554 | 360-516 | 157 | 29 | 49 | 42 | 37 | 57.96 | -74.89 |
| JX010235 *Colletotrichum kahawae* subsp. kahawae C1275.14 | 593 | 388-544 | 157 | 30 | 49 | 42 | 36 | 57.96 | -75.69 |
| JQ005220 *Colletotrichum karstii* CBS:106.91 | 555 | 360-517 | 158 | 30 | 49 | 42 | 37 | 57.59 | -73.43 |
| GU227843 *Colletotrichum lineola* CBS:282.85 | 515 | 341-496 | 156 | 33 | 42 | 41 | 40 | 53.21 | -73.48 |
| JX546819 *Colletotrichum lindemuthianum* CBS:130841 | 546 | 329-488 | 160 | 33 | 59 | 41 | 27 | 62.50 | -69.80 |
| JX519222 *Colletotrichum dracaenophilum* CBS:118199 | 527 | 330-489 | 160 | 37 | 54 | 42 | 27 | 60.00 | -77.82 |
| KF687718 *Colletotrichum magnisporum* CBS 398.84 | 534 | 339-496 | 158 | 28 | 46 | 44 | 40 | 56.96 | -74.23 |
| KC566800 *Colletotrichum musae* CBS 125356 | 536 | 341-498 | 158 | 32 | 49 | 41 | 36 | 56.96 | -74.03 |
| KC204999 *Colletotrichum nymphaeae* CPC 20915 | 540 | 351-507 | 157 | 32 | 49 | 41 | 35 | 57.32 | -71.60 |
| JX145174 *Colletotrichum nupharicola* CBS 472 | 554 | 360-517 | 158 | 30 | 50 | 42 | 36 | 58.23 | -76.19 |
| KF178468 *Colletotrichum orbiculare* CBS 133197 | 529 | 332-491 | 160 | 33 | 58 | 41 | 28 | 61.88 | -69.80 |
| KF687722 *Colletotrichum pseudomajus* CBS 571.88 | 519 | 326-481 | 156 | 30 | 45 | 42 | 39 | 55.77 | -71.83 |
| JQ948480 *Colletotrichum pseudoacutatum* CBS:436.77 | 543 | 354-510 | 157 | 32 | 49 | 41 | 35 | 57.32 | -73.79 |
| JQ005233 *Colletotrichum parsonsiae* CBS:128525 | 555 | 361-517 | 157 | 29 | 47 | 42 | 39 | 56.69 | -73.89 |
| JQ005227 *Colletotrichum petchii* CBS:118193 | 554 | 360-516 | 157 | 30 | 45 | 42 | 40 | 55.41 | -70.59 |

| | | | | | | | | | |
|---|---|---|---|---|---|---|---|---|---|
| JX010258 *Colletotrichum siamense* C1316.6 | 593 | 388-544 | 157 | 31 | 48 | 41 | 37 | 56.69 | -74.09 |
| KC566824 *Colletotrichum siamense* CBS 194.32 | 535 | 341-497 | 157 | 31 | 47 | 41 | 38 | 56.05 | -74.09 |
| GU227853 *Colletotrichum spinaciae* CBS:129.57 | 514 | 341-495 | 155 | 31 | 47 | 40 | 37 | 56.13 | -70.78 |
| JX010294 *Colletotrichum theobromicola* C1316.13 | 596 | 390-547 | 158 | 31 | 50 | 41 | 36 | 57.59 | -74.03 |
| KC566806 *Colletotrichum tropicale* CBS 124946 | 536 | 341-498 | 158 | 31 | 49 | 41 | 37 | 56.96 | -74.03 |
| GU227888 *Colletotrichum truncatum* CBS:127.57 | 522 | 347-503 | 157 | 31 | 42 | 42 | 42 | 53.50 | -71.93 |
| GU227817 *Colletotrichum trichellum* CBS:125343 | 520 | 345-502 | 158 | 30 | 49 | 41 | 38 | 56.96 | -72.49 |
| KC820802 *Colletotrichum queenslandicum* KC1 | 554 | 348-505 | 158 | 31 | 50 | 41 | 36 | 57.59 | -74.03 |
| KJ619456 *Colletotrichum boninense* Z-D-07 | 597 | 381-538 | 158 | 30 | 49 | 42 | 37 | 57.59 | -73.43 |
| KM229695 *Colletotrichum gloeosporioides* B5 | 575 | 360-517 | 158 | 31 | 49 | 41 | 37 | 56.96 | -74.03 |
| KM111483 *Colletotrichum gloeosporioides* USM2-6A | 553 | 349-505 | 157 | 31 | 48 | 41 | 37 | 56.69 | -74.09 |
| KM357453 *Colletotrichum gloeosporioides* C1 | 584 | 360-517 | 158 | 31 | 49 | 41 | 37 | 56.96 | -74.03 |
| KM357563 *Colletotrichum tropicale* CG10 | 545 | 346-502 | 157 | 31 | 47 | 41 | 38 | 56.05 | -74.09 |
| KJ813612 *Colletotrichum siamense* OCAC22 | 598 | 388-544 | 157 | 31 | 47 | 41 | 38 | 56.05 | -74.09 |
| KJ813595 *Colletotrichum karstii* OCAC4 | 618 | 407-564 | 158 | 30 | 49 | 42 | 37 | 57.59 | -73.43 |
| | | | **157.42±1.03** | | | | | **57.11±1.57** | **-73.96±2.20** |
| ***Xylaria*** | | | | | | | | | |
| *Xylaria* sp. Ps-31 (KU671358) | 549 | 352-509 | 158 | 30 | 43 | 37 | 48 | 50.63 | -71.94 |
| *Xylaria* sp. Ps-29 (KU671356) | 562 | 353-521 | 169 | 28 | 44 | 40 | 57 | 49.70 | -68.22 |
| AY909011 *Xylaria hypoxylon* CBS 868.72 | 510 | 344-510 | 159 | 32 | 47 | 37 | 43 | 52.83 | -59.84 |
| AM993138 *Xylaria hypoxylon* CBS121680 | 522 | 340-522 | 159 | 34 | 46 | 37 | 42 | 52.20 | -72.24 |
| EU009958 *Xylaria* sp. Vega366 | 581 | 384-541 | 158 | 30 | 42 | 37 | 49 | 50.00 | -71.94 |
| KJ154952 *Xylaria brevipes* MP748 | 594 | 381-547 | 167 | 28 | 45 | 40 | 54 | 50.90 | -68.13 |
| *Arthroxylaria* sp. Fi-29 (KU671324) | 561 | 357-516 | 160 | 36 | 45 | 35 | 44 | 50.00 | -61.08 |
| AF432179 *Arthroxylaria elegans* CBS 537.79 | 577 | 369-528 | 160 | 35 | 45 | 37 | 43 | 51.25 | -63.43 |
| | | | **161.25±4.27** | | | | | **50.94±1.11** | **-67.10±5.04** |
| ***Preussia*** | | | | | | | | | |
| *Preussia* sp. Ae-09 (KU663485) | 516 | 316-470 | 155 | 29 | 47 | 42 | 37 | 57.42 | -79.23 |
| AY943044 *Preussia aemulans* CBS 120.66 | 519 | 313-466 | 154 | 30 | 50 | 44 | 30 | 61.04 | -79.29 |
| AY943052 *Preussia australis* ATCC 22797 | 534 | 323-474 | 152 | 30 | 44 | 39 | 39 | 54.61 | -64.67 |
| AY943060 *Preussia fleischhakii* CBS 708.82 | 521 | 315-468 | 154 | 30 | 51 | 43 | 30 | 61.04 | -79.69 |

| | | | | | | | | | |
|---|---|---|---|---|---|---|---|---|---|
| NR_077168 *Preussia flanaganii* CBS 112.73 | 529 | 323-476 | 154 | 30 | 51 | 43 | 30 | 61.04 | -79.69 |
| AY943059 *Preussia funiculata* ATCC 16294 | 529 | 316-470 | 155 | 30 | 51 | 44 | 30 | 61.29 | -79.23 |
| AY943058 *Preussia isomera* CBS 318.65 | 527 | 318-474 | 157 | 27 | 53 | 46 | 31 | 63.06 | -87.09 |
| GU183123 *Preussia minimoides* NRRL 37629 | 1003 | 298-445 | 148 | 30 | 40 | 41 | 37 | 54.73 | -78.58 |
| DQ468026 *Preussia minima* CBS 52450 | 464 | 306-464 | 145 | 29 | 42 | 38 | 36 | 55.17 | -59.13 |
| GQ292750 *Preussia persica* IRAN 844 | 473 | 306-473 | 153 | 30 | 44 | 43 | 36 | 56.86 | -71.71 |
| GQ292749 *Preussia polymorpha* IRAN 845 | 529 | 318-472 | 155 | 27 | 55 | 44 | 29 | 63.87 | -77.38 |
| DQ468028 *Preussia similis* CBS 80473 | 475 | 309-475 | 153 | 31 | 45 | 40 | 37 | 55.56 | -75.38 |
| KF128811 *Preussia* sp. GSH1_5_6 | 541 | 337-490 | 154 | 28 | 45 | 43 | 38 | 57.14 | -79.48 |
| | | | **153.00±3.19** | | | | | **58.68±3.31** | **-76.20±7.27** |
| ***Setosphaeria*** | | | | | | | | | |
| *Setosphaeria rostrata* Ae-06 (KU663482) | 567 | 352-532 | 181 | 34 | 50 | 42 | 55 | 50.83 | -68.03 |
| *Setosphaeria rostrata* Fi-14 (KU671309) | 578 | 358-536 | 179 | 34 | 50 | 42 | 53 | 51.40 | -75.53 |
| *Setosphaeria rostrata* Ps-16 (KU671343) | 571 | 351-530 | 180 | 34 | 50 | 42 | 54 | 51.11 | -75.83 |
| *Setosphaeria rostrata* Ps-18 (KU671345) | 573 | 351-529 | 179 | 34 | 50 | 42 | 53 | 51.40 | -75.53 |
| KT265259 *Setosphaeria pedicellata* CBS 375.76 | 713 | 505-670 | 166 | 32 | 48 | 40 | 46 | 53.01 | -73.17 |
| KT265245 *Setosphaeria rostrata* CBS 128060 | 742 | 515-693 | 179 | 34 | 51 | 42 | 52 | 51.96 | -77.83 |
| KT265239 *Setosphaeria rostrata* CBS 128063 | 742 | 514-694 | 181 | 34 | 50 | 44 | 53 | 51.93 | -80.53 |
| KF278460 *Setosphaeria turcica* ATCC 64835 | 561 | 357-522 | 166 | 31 | 46 | 41 | 48 | 52.41 | -74.36 |
| KP132777 *Setosphaeria rostrata* WM 11.61 | 610 | 377-555 | 179 | 34 | 50 | 42 | 53 | 51.40 | -75.53 |
| | | | **176.67±6.10** | | | | | **51.72±0.68** | **-75.15±3.40** |
| ***Cochliobolus/Curvularia*** | | | | | | | | | |
| *Cochliobolus* sp. Fi-08 (KU671303) | 538 | 333-493 | 161 | 35 | 45 | 39 | 42 | 52.17 | -68.83 |
| *Curvularia lunata* Ps-09 (KU671336) | 567 | 357-519 | 163 | 34 | 42 | 40 | 47 | 50.31 | -66.46 |
| KJ922375 *Curvularia inaequalis* CBS 102.42 | 473 | 279-442 | 164 | 31 | 45 | 40 | 48 | 51.83 | -74.01 |
| AF120261 *Curvularia inaequalis* CBS 185.47 | 524 | 324-486 | 163 | 30 | 45 | 40 | 48 | 52.15 | -74.05 |
| AF120257 *Cochliobolus homomorphus* ATCC26651 | 545 | 338-507 | 170 | 34 | 39 | 40 | 57 | 46.47 | -67.93 |
| AF071349 *Cochliobolus homomorphus* ATCC 13409 | 511 | 331-511 | 166 | 33 | 45 | 40 | 48 | 51.20 | -68.04 |
| JN601029 Cochliobolus hawaiiensis CBS 173.57 | 518 | 306-468 | 163 | 36 | 46 | 40 | 41 | 52.76 | -72.13 |
| GQ328851 Cochliobolus lunatus NRRL:53719 | 1103 | 351-513 | 163 | 34 | 42 | 40 | 47 | 50.31 | -66.46 |
| KJ922376 Curvularia protuberata CBS 376.65 | 471 | 279-439 | 161 | 30 | 45 | 40 | 46 | 52.80 | -76.68 |

| | | | | | | | | | |
|---|---|---|---|---|---|---|---|---|---|
| AY004779 Bipolaris portulacae CBS 403.72 | 570 | 380-543 | 164 | 34 | 43 | 41 | 46 | 51.22 | -76.63 |
| JN192387 Curvularia spicifera CBS 274.52 | 516 | 305-467 | 163 | 34 | 45 | 39 | 45 | 51.53 | -71.83 |
| JX256433 Curvularia tuberculata CBS 146-63 | 463 | 278-444 | 167 | 33 | 44 | 40 | 50 | 50.30 | -71.01 |
| JN192374 Curvularia tuberculata CBS 146.63 | 521 | 335-502 | 168 | 33 | 44 | 40 | 51 | 50.00 | -70.97 |
| JN712459 Curvularia trifolii CPC 2995 | 621 | 377-543 | 167 | 33 | 48 | 39 | 47 | 52.10 | -72.57 |
| KJ433660 Curvularia lunata IARI-IF6 | 602 | 378-540 | 163 | 34 | 42 | 40 | 47 | 50.31 | -66.46 |
| KJ638720 Cochliobolus sp. VJ18 | 544 | 337-497 | 161 | 35 | 45 | 39 | 42 | 52.17 | -68.83 |
| | | | **164.19±2.67** | | | | | **51.10±1.55** | **-70.81±3.42** |
| ***Stagonosporopsis*** | | | | | | | | | |
| *Stagonosporopsis* sp. Ae-20 (KU663496) | 522 | 327-484 | 158 | 31 | 44 | 38 | 45 | 51.90 | -62.28 |
| GU237734 *Stagonosporopsis actaeae* CBS 106.96 | 490 | 306-464 | 159 | 31 | 46 | 38 | 44 | 52.83 | -61.48 |
| GU237906 *Stagonosporopsis crystalliniformis* CBS 771.85 | 488 | 306-462 | 157 | 31 | 45 | 38 | 43 | 52.87 | -62.78 |
| GU237821 *Stagonosporopsis caricae* CBS 282.76 | 489 | 307-463 | 157 | 32 | 45 | 37 | 43 | 52.23 | -62.08 |
| GU237899 *Stagonosporopsis dennisii* CBS 631.68 | 488 | 306-462 | 157 | 32 | 45 | 37 | 43 | 52.23 | -62.08 |
| GU237893 *Stagonosporopsis hortensis* CBS 572.85 | 489 | 306-463 | 158 | 33 | 42 | 38 | 45 | 50.63 | -59.81 |
| GU237730 *Stagonosporopsis hortensis* CBS 104.42 | 489 | 306-463 | 158 | 33 | 42 | 37 | 46 | 50.00 | -60.18 |
| GU237747 *Stagonosporopsis heliopsidis* CBS 109182 | 488 | 306-462 | 157 | 31 | 45 | 37 | 44 | 52.23 | -62.08 |
| GU237844 *Stagonosporopsis lupini* CBS 375.84 | 488 | 306-462 | 157 | 32 | 42 | 37 | 46 | 50.32 | -62.48 |
| GU237832 *Stagonosporopsis valerianellae* CBS 329.67 | 487 | 306-461 | 156 | 32 | 44 | 38 | 42 | 52.56 | -62.28 |
| HM992815 *Stagonosporopsis cucurbitacearum* NY6960a | 512 | 317-474 | 158 | 32 | 44 | 37 | 45 | 51.27 | -61.58 |
| | | | **157.45±0.82** | | | | | **51.73±1.02** | **-61.74±0.94** |
| ***Peyronellaea*** | | | | | | | | | |
| *Peyronellaea glomerata* Fi-15 (KU671310) | 506 | 314-469 | 156 | 31 | 42 | 37 | 46 | 50.64 | -61.03 |
| FJ426975 *Peyronellaea americana* CBS 112525 | 485 | 304-459 | 156 | 32 | 44 | 36 | 44 | 51.28 | -62.63 |
| GU237840 *Peyronellaea anserina* CBS 363.91 | 485 | 304-459 | 156 | 31 | 44 | 37 | 44 | 51.92 | -65.03 |
| GU237778 *Peyronellaea alectorolophi* CBS 132.96 | 485 | 304-459 | 156 | 32 | 42 | 37 | 45 | 50.64 | -61.93 |
| FJ426994 *Peyronellaea coffeae-arabicae* CBS 123398 | 485 | 304-459 | 156 | 32 | 44 | 36 | 44 | 51.28 | -62.63 |
| FJ426983 *Peyronellaea calorpreferens* CBS 109.92 | 485 | 304-459 | 156 | 32 | 42 | 37 | 45 | 50.64 | -62.33 |
| GU237878 *Peyronellaea eucalyptica* CBS 508.91 | 486 | 304-460 | 157 | 32 | 43 | 36 | 46 | 50.32 | -62.53 |
| FJ427016 *Peyronellaea glomerata* CBS 112448 | 485 | 304-459 | 156 | 32 | 42 | 36 | 46 | 50.00 | -60.73 |

| | | | | | | | | | |
|---|---|---|---|---|---|---|---|---|---|
| FJ427054 *Peyronellaea pomorum var. circinata* CBS 286.76 | 485 | 304-459 | 156 | 31 | 43 | 36 | 46 | 50.64 | -61.35 |
| GU237823 *Peyronellaea pinodes* CBS 285.49 | 485 | 304-459 | 156 | 32 | 42 | 37 | 45 | 50.64 | -64.93 |
| FJ427052 *Peyronellaea pinodella* CBS 531.66 | 484 | 304-458 | 156 | 32 | 42 | 37 | 45 | 50.64 | -64.33 |
| KJ780820 *Peyronellaea glomerata* XF60 | 541 | 328-483 | 156 | 32 | 42 | 36 | 46 | 50.00 | -60.73 |
| | | | **156.08±0.29** | | | | | **50.72±0.55** | **-62.52±1.53** |
| ***Alternaria*** | | | | | | | | | |
| *Alternaria alternata* Ae-14 (KU663490) | 533 | 341-500 | 160 | 31 | 42 | 37 | 50 | 49.38 | -63.27 |
| *Alternaria alternata* Fi-02 (KU671297) | 540 | 335-494 | 160 | 31 | 42 | 37 | 50 | 49.38 | -63.27 |
| *Alternaria alternata* Fi-09 (KU671304) | 540 | 336-495 | 160 | 31 | 42 | 37 | 50 | 49.38 | -63.27 |
| *Alternaria alternata* Fi-12 (KU671307) | 541 | 337-495 | 159 | 31 | 42 | 37 | 49 | 49.69 | -65.25 |
| *Alternaria alternata* Fi-17 (KU671312) | 534 | 334-492 | 159 | 31 | 42 | 37 | 49 | 49.69 | -65.25 |
| *Alternaria alternata* Fi-30 (KU671325) | 540 | 339-497 | 159 | 31 | 42 | 37 | 49 | 49.69 | -65.25 |
| *Alternaria alternata* Ps-04 (KU671331) | 535 | 338-496 | 159 | 31 | 42 | 37 | 49 | 49.69 | -65.25 |
| *Alternaria alternata* Ps-06 (KU671333) | 542 | 336-494 | 159 | 30 | 43 | 37 | 49 | 50.31 | -65.25 |
| *Alternaria alternata* Ps-11 (KU671338) | 536 | 336-495 | 160 | 31 | 42 | 37 | 50 | 49.38 | -63.27 |
| *Alternaria alternata* Ps-27 (KU671354) | 533 | 336-494 | 159 | 31 | 42 | 37 | 49 | 49.69 | -65.25 |
| KM030317 *Alternaria alternata* NRRL 66024 | 1059 | 311-469 | 159 | 31 | 43 | 37 | 48 | 50.31 | -65.74 |
| GQ328850 *Alternaria al*ternata NRRL:54028 | 978 | 276-436 | 161 | 31 | 42 | 37 | 51 | 49.06 | -63.20 |
| GQ328849 *Alternaria alternata* NRRL:54027 | 1057 | 303-462 | 160 | 31 | 42 | 37 | 50 | 49.38 | -63.27 |
| AF229470 *Alternaria porri* ATCC 58175 | 542 | 340-503 | 164 | 26 | 50 | 38 | 50 | 53.66 | -65.74 |
| KJ718240 *Alternaria solani* CBS 116442 | 526 | 336-500 | 165 | 26 | 51 | 38 | 50 | 53.94 | -72.27 |
| JF694935 *Alternaria* sp. ZJ-2008017 | 576 | 357-515 | 159 | 31 | 42 | 37 | 49 | 49.69 | -65.25 |
| KJ412490 *Alternaria alternata* JG32 | 559 | 353-512 | 160 | 31 | 42 | 37 | 50 | 49.38 | -63.27 |
| KJ735925 *Alternaria alternata* SR/II/9 | 605 | 363-521 | 159 | 31 | 42 | 37 | 49 | 49.69 | -65.25 |
| KM051397 *Alternaria* sp. BAB-4031 | 564 | 336-494 | 159 | 31 | 42 | 37 | 49 | 49.69 | -65.25 |
| | | | **160.00±1.70** | | | | | **50.06±1.35** | **-64.94±2.05** |
