# Supplementary material for: Internal transcribed spacer 2 (ITS2) molecular morphometric analysis based species delimitation of foliar endophytic fungi from Aglaia elaeagnoidea, Flacourtia inermis and Premna serratifolia
Source: PLoS One. 2019 Apr 9;14(4):e0215024. doi: 10.1371/journal.pone.0215024 (PMC6456209; doi:10.1371/journal.pone.0215024)
Supplement: S3 File — (PDF) [file pone.0215024.s003.pdf]

Supplementary File 3

Table A. Summary of compensatory base changes detected among the *Colletotrichum* spp. recovered in this study

		01	02	03	04	05	06	07	08	09	10	11	12	13	14	15	16	17	18	19	20	21	22	23	24	25	26	27	28
01	AF129286 *Lobaria pulmonaria* L22	0																											
02	AF329172 *Ochrolechia balcanica*	1	0																										
03	*Colletotrichum tropicale* Ae-05 (KU663481)	6	5	0																									
04	*Colletotrichum* sp. Ae-07 (KU663483)	6	5	0	0																								
05	*Colletotrichum* sp. Ae-08 (KU663484)	5	4	0	0	0																							
06	*Colletotrichum* sp. Ae-10 (KU663486)	6	5	0	0	0	0																						
07	*Colletotrichum* sp. Ae-13 (KU663489)	6	5	0	0	0	0	0																					
08	*Colletotrichum gloeosporioides* Ae-15 (KU663491)	6	5	0	0	0	0	0	0																				
09	*Colletotrichum* sp. Ae-16 (KU663492)	6	5	0	0	0	0	0	0	0																			
10	*Colletotrichum* sp. Ae-17 (KU663493)	6	5	0	0	0	0	0	0	0	0																		
11	*Colletotrichum gloeosporioides* Ae-18 (KU663494)	6	5	0	0	0	0	0	0	0	0	0																	
12	*Colletotrichum karstii* Ae-21 (KU663497)	6	5	1	1	1	1	1	1	1	1	1	0																
13	*Colletotrichum* sp. Ae-23 (KU663499)	6	5	0	0	0	0	0	0	0	0	0	1	0															
14	*Colletotrichum karstii* Fi-01 (KU671296)	6	5	1	1	1	1	1	1	1	1	1	0	1	0														
15	*Colletotrichum gloeosporioides* Fi-05 (KU671300)	6	5	0	0	0	0	0	0	0	0	0	1	0	1	0													
16	*Colletotrichum gloeosporioides* Fi-11 (KU671306)	6	5	0	0	0	0	0	0	0	0	0	1	0	1	0	0												
17	*Colletotrichum* sp. Ps-05 (KU671332)	6	5	0	0	0	0	0	0	0	0	0	1	0	1	0	0	0											
18	*Colletotrichum cliviae* Ps-07 (KU671334)	6	5	1	1	1	1	1	1	1	1	1	0	1	0	1	1	1	0										
19	*Colletotrichum* sp. Ps-14 (KU671341)	6	5	0	0	0	0	0	0	0	0	0	1	0	1	0	0	0	1	0									
20	*Colletotrichum karstii* Ps-19 (KU671346)	6	5	1	1	1	1	1	1	1	1	1	0	1	0	1	1	1	0	1	0								
21	*Colletotrichum* sp. Ps-23 (KU671350)	6	5	0	0	0	0	0	0	0	0	0	1	0	1	0	0	0	1	0	1	0							
22	*Colletotrichum karstii* Ps-24 (KU671351)	6	5	1	1	1	1	1	1	1	1	1	0	1	0	1	1	1	0	1	0	1	0						
23	*Colletotrichum gloeosporioides* Ps-25 (KU671352)	6	5	0	0	0	0	0	0	0	0	0	1	0	1	0	0	0	1	0	1	0	1	0					
24	*Colletotrichum gloeosporioides* Ps-26 (KU671353)	6	5	0	0	0	0	0	0	0	0	0	1	0	1	0	0	0	1	0	1	0	1	0	0				
25	*Colletotrichum cliviae* Ps-28 (KU671355)	6	5	1	1	1	1	1	1	1	1	1	0	1	0	1	1	1	0	1	0	1	0	1	1	0			
26	*Colletotrichum karstii* Ps-30 (KU671357)	6	5	1	1	1	1	1	1	1	1	1	0	1	0	1	1	1	0	1	0	1	0	1	1	0	0		
27	*Colletotrichum gloeosporioides* Ps-36 (KU671363)	6	5	0	0	0	0	0	0	0	0	0	1	0	1	0	0	0	1	0	1	0	1	0	0	1	1	0	
28	*Colletotrichum* sp. Ps-37 (KU671364)	6	5	0	0	0	0	0	0	0	0	0	1	0	1	0	0	0	1	0	1	0	1	0	0	1	1	0	0

Table B. Summary of compensatory base changes detected among the *Diaporthe* spp. and *Phomopsis* spp. recovered in this study

		01	02	03	04	05	06	07	08	09	10	11	12	13	14	15	16	17	18	19	20	21	22	23	24	25	26	27	28	29	30	31	32	33	34	35	36	37	38	39
01	AF129286 *Lobaria pulmonaria* L22	0																																						
02	AF329172 *Ochrolechia balcanica*	1	0																																					
03	*Diaporthe pseudomangiferae* Ae-02 (KU663478)	3	3	0																																				
04	*Diaporthe pseudomangiferae* Ae-19 (KU663495)	3	3	0	0																																			
05	*Diaporthe pascoei* Ae-22 (KU663498)	5	5	1	1	0																																		
06	*Diaporthe* sp. Ae-24 (KU663500)	4	4	1	1	0	0																																	
07	*Diaporthe pseudomangiferae* Ae-25 (KU663501)	3	3	0	0	1	1	0																																
08	*Diaporthe perseae* Ae-28 (KU663504)	5	5	1	1	0	0	1	0																															
09	*Diaporthe pseudomangiferae* Ae-29 (KU663505)	3	3	0	0	1	1	0	1	0																														
10	*Diaporthe pseudomangiferae* Fi-03 (KU671298)	3	3	0	0	1	1	0	1	0	0																													
11	*Phomopsis* sp. Fi-04 (KU671299)	4	4	1	1	0	0	1	0	1	1	0																												
12	*Diaporthe* sp. Fi-06 (KU671301)	4	4	1	1	0	0	1	0	1	1	0	0																											
13	*Diaporthe* sp. Fi-07 (KU671302)	4	4	1	1	0	0	1	0	1	1	0	0	0																										
14	*Diaporthe* sp. Fi-18 (KU671313)	4	4	1	1	0	0	1	0	1	1	0	0	0	0																									
15	*Diaporthe pseudomangiferae* Fi-19 (KU671314)	3	3	0	0	1	1	0	1	0	0	1	1	1	1	0																								
16	*Diaporthe* sp. Fi-20 (KU671315)	3	3	0	0	1	1	0	1	0	0	1	1	1	1	0	0																							
17	*Diaporthe* sp. Fi-21 (KU671316)	4	4	1	1	0	0	1	0	1	1	0	0	0	0	1	1	0																						
18	*Diaporthe* sp. Fi-22 (KU671317)	4	4	1	1	0	0	1	0	1	1	0	0	0	0	1	1	0	0																					
19	*Diaporthe* sp. Fi-23 (KU671318)	4	4	1	1	0	0	1	0	1	1	0	0	0	0	1	1	0	0	0																				
20	*Phomopsis* sp. Fi-24 (KU671319)	3	3	0	0	0	0	0	0	0	0	0	0	0	0	0	0	0	0	0	0																			
21	*Diaporthe* sp. Fi-25 (KU671320)	4	4	1	1	0	0	1	0	1	1	0	0	0	0	1	1	0	0	0	0	0																		
22	*Diaporthe pseudomangiferae* Fi-26 (KU671321)	3	3	0	0	2	2	0	2	0	0	2	2	2	2	0	0	2	2	1	0	2	0																	
23	*Diaporthe* sp. Fi-27 (KU671322)	4	4	1	1	0	0	1	0	1	1	0	0	0	0	1	1	0	0	0	0	0	2	0																
24	*Diaporthe* sp. Fi-28 (KU671323)	5	5	1	1	0	0	1	0	1	1	0	0	0	0	1	1	0	0	0	0	0	2	0	0															
25	*Phomopsis* sp. Fi-31 (KU671326)	3	3	0	0	0	0	0	0	0	0	0	0	0	0	0	0	0	0	0	1	0	0	0	0	0														
26	*Phomopsis* sp. Fi-32 (KU671327)	3	3	0	0	0	0	0	0	0	0	0	0	0	0	0	0	0	0	0	1	0	0	0	0	0	0													
27	*Diaporthe* sp. Ps-01 (KU671328)	4	4	1	1	0	0	1	0	1	1	0	0	0	0	1	1	0	0	0	0	0	2	0	0	0	0	0												
28	*Diaporthe* sp. Ps-02 (KU671329)	3	3	0	0	1	1	0	1	0	0	1	1	1	1	0	0	1	1	1	0	1	0	1	1	0	0	1	0											
29	*Diaporthe pseudomangiferae* Ps-08 (KU671335)	3	3	0	0	1	1	0	1	0	0	1	1	1	1	0	0	1	1	1	0	1	0	1	1	0	0	1	0	0										
30	*Phomopsis* sp. Ps-12 (KU671339)	3	3	0	0	0	0	0	0	0	0	0	0	0	0	0	0	0	0	0	0	0	0	0	0	1	1	0	0	0	0									
31	*Diaporthe* sp. Ps-13 (KU671340)	5	5	1	1	1	1	1	1	1	1	1	1	1	1	1	1	1	1	1	2	1	1	1	1	2	2	1	1	1	2	0								
32	*Diaporthe pseudomangiferae* Ps-15 (KU671342)	3	3	0	0	1	1	0	1	0	0	1	1	1	1	0	0	1	1	1	0	1	0	1	1	0	0	1	0	0	0	1	0							
33	*Phomopsis* sp. Ps-20 (KU671347)	3	3	0	0	0	0	0	0	0	0	0	0	0	0	0	0	0	0	0	1	0	0	0	0	0	0	0	0	0	1	2	0	0						
34	*Diaporthe melonis* Ps-21 (KU671348)	4	4	0	0	1	1	0	1	0	0	1	1	1	1	0	0	1	1	1	0	1	0	1	1	0	0	1	0	0	0	1	0	0	0					
35	*Phomopsis azadirachtae* Ps-22 (KU671349)	3	3	0	0	0	0	0	0	0	0	0	0	0	0	0	0	0	0	0	0	0	0	0	0	0	0	0	0	0	0	2	0	0	0	0				
36	*Diaporthe perseae* Ps-32 (KU671359)	5	5	1	1	0	0	1	0	1	1	0	0	0	0	1	1	0	0	0	0	0	2	0	0	0	0	0	1	1	0	1	1	0	1	0	0			
37	*Diaporthe* sp. Ps-33 (KU671360)	4	4	1	1	0	0	1	0	1	1	0	0	0	0	1	1	0	0	0	0	0	2	0	0	0	0	0	1	1	0	1	1	0	1	0	0	0		
38	*Phomopsis* sp. Ps-34 (KU671361)	4	4	1	1	2	2	1	2	1	1	2	2	2	2	1	1	2	2	2	1	2	1	2	2	1	1	2	1	1	1	2	1	1	1	1	2	2	0	
39	*Phomopsis* sp. Ps-35 (KU671362)	4	4	1	1	0	0	1	0	1	1	0	0	0	0	1	1	0	0	0	0	0	2	0	0	0	0	0	1	1	0	1	1	0	1	0	0	0	2	0

Table C. Summary of compensatory base changes detected among the genera except for *Colletotrichum*, *Diaporthe*, and *Phomopsis*, recovered in this study

		01	02	03	04	05	06	07	08	09	10	11	12	13	14	15	16	17	18	19	20	21	22	23	24	25	26	27	28	29	30	31	32	33	34	35	36	37
01	AF129286 *Lobaria pulmonaria* L22	0																																				
02	AF329172 *Ochrolechia balcanica*	1	0																																			
03	*Setosphaeria rostrata* Ae-06 (KU663482)	0	0	0																																		
04	*Setosphaeria rostrata* Fi-14 (KU671309)	0	0	0	0																																	
05	*Setosphaeria rostrata* Ps-16 (KU671343)	0	0	0	0	0																																
06	*Setosphaeria rostrata* Ps-18 (KU671345)	0	0	0	0	0	0																															
07	*Cochliobolus* sp. Fi-08 (KU671303)	1	1	2	2	2	2	0																														
08	*Curvularia lunata* Ps-09 (KU671336)	1	1	5	5	5	5	2	0																													
09	*Phyllosticta hymenocallidicola* Ae-03 (KU663479)	2	1	0	0	0	0	2	2	0																												
10	*Phyllosticta capitalensis* Ae-11 (KU663487)	1	3	0	0	0	0	3	3	1	0																											
11	*Guignardia mangiferae* Ae-26 (KU663502)	1	3	0	0	0	0	3	3	1	0	0																										
12	*Guignardia mangiferae* Fi-10 (KU671305)	1	3	0	0	0	0	3	3	1	0	0	0																									
13	*Phyllosticta capitalensis* Fi-13 (KU671308)	1	3	0	0	0	0	3	3	1	0	0	0	0																								
14	*Phyllosticta capitalensis* Fi-16 (KU671311)	1	3	0	0	0	0	3	3	1	0	0	0	0	0																							
15	*Neofusicoccum parvum* Ps-03 (KU671330)	2	2	0	0	0	0	3	3	0	1	1	1	1	1	0																						
16	*Neofusicoccum parvum* Ps-10 (KU671337)	2	2	0	0	0	0	3	3	0	1	1	1	1	1	0	0																					
17	*Neofusicoccum parvum* Ps-17 (KU671344)	2	2	0	0	0	0	3	3	0	1	1	1	1	1	0	0	0																				
18	*Stagonosporopsis* sp. Ae-20 (KU663496)	2	3	0	0	0	0	2	2	1	3	3	3	3	3	3	3	3	0																			
19	*Pseudofusicoccum adansoniae* Ae-01 (KU663477)	3	2	0	0	0	0	3	3	0	1	1	1	1	1	0	0	0	3	0																		
20	*Peyronellaea glomerata* Fi-15 (KU671310)	2	3	0	0	0	0	2	2	1	3	3	3	3	3	3	3	3	0	3	0																	
21	*Neoscytalidium dimidiatum* Ae-27 (KU663503)	3	2	0	0	0	0	3	3	1	2	2	2	2	2	1	1	1	3	1	3	0																
22	*Harknessia* sp. Ae-04 (KU663480)	6	7	1	1	1	1	3	3	6	9	9	9	9	9	7	7	7	3	6	3	7	0															
23	*Aureobasidium thailandense* Ae-12 (KU663488)	2	2	0	0	0	0	0	0	2	2	2	2	2	2	2	2	2	2	2	2	2	4	0														
24	*Xylaria* sp. Ps-31 (KU671358)	3	2	5	5	5	5	3	4	4	3	3	3	3	3	3	3	3	2	2	2	3	2	2	0													
25	*Xylaria* sp. Ps-29 (KU671356)	3	2	5	5	5	5	3	3	4	3	3	3	3	3	3	3	3	2	2	2	3	2	2	0	0												
26	*Arthroxylaria* sp. Fi-29 (KU671324)	4	3	1	1	1	1	1	1	4	4	4	4	4	4	4	4	4	1	3	1	4	4	2	1	1	0											
27	*Preussia* sp. Ae-09 (KU663485)	0	0	1	1	1	1	0	3	1	1	1	1	1	1	1	1	1	1	1	1	1	2	1	4	4	3	0										
28	*Alternaria alternata* Ae-14 (KU663490)	1	1	2	2	2	2	1	2	1	2	2	2	2	2	2	2	2	1	2	1	2	3	0	1	1	1	1	0									
29	*Alternaria alternata* Fi-02 (KU671297)	1	1	2	2	2	2	1	2	1	2	2	2	2	2	2	2	2	1	2	1	2	3	0	1	1	1	1	0	0								
30	*Alternaria alternata* Fi-09 (KU671304)	1	1	2	2	2	2	1	2	1	2	2	2	2	2	2	2	2	1	2	1	2	3	0	1	1	1	1	0	0	0							
31	*Alternaria alternata* Fi-12 (KU671307)	1	1	2	2	2	2	1	2	2	3	3	3	3	3	3	3	3	1	3	1	3	3	1	1	1	1	1	0	0	0	0						
32	*Alternaria alternata* Fi-17 (KU671312)	1	1	2	2	2	2	1	2	2	3	3	3	3	3	3	3	3	1	3	1	3	3	1	1	1	1	1	0	0	0	0	0					
33	*Alternaria alternata* Fi-30 (KU671325)	1	1	2	2	2	2	1	2	2	3	3	3	3	3	3	3	3	1	3	1	3	3	1	1	1	1	1	0	0	0	0	0	0				
34	*Alternaria alternata* Ps-04 (KU671331)	1	1	2	2	2	2	1	2	2	3	3	3	3	3	3	3	3	1	3	1	3	3	1	1	1	1	1	0	0	0	0	0	0	0			
35	*Alternaria alternata* Ps-06 (KU671333)	1	1	2	2	2	2	1	2	2	3	3	3	3	3	3	3	3	1	3	1	3	3	1	1	1	1	1	0	0	0	0	0	0	0	0		
36	*Alternaria alternata* Ps-11 (KU671338)	1	1	2	2	2	2	1	2	1	2	2	2	2	2	2	2	2	1	2	1	2	3	0	1	1	1	1	0	0	0	0	0	0	0	0	0	
37	*Alternaria alternata* Ps-27 (KU671354)	1	1	2	2	2	2	1	2	2	3	3	3	3	3	3	3	3	1	3	1	3	3	1	1	1	1	1	0	0	0	0	0	0	0	0	0	0
